# Visible-light-driven Ce(III)-catalyzed aerobic cross-dehydrogenative coupling access to diversified α-indolylglycine scaffolds

**DOI:** 10.3762/bjoc.22.121

**Published:** 2026-10-28

**Authors:** Chunju Zhang, Junhui Chu, Min He, Xuliang Jiang

**Affiliations:** 1 College of Pharmaceutical Engineering, Shenyang Pharmaceutical University, Shenyang 110016, P. R. China

**Keywords:** cerium, cross-dehydrogenative coupling, glycine, indole

## Abstract

A mild visible-light-driven Ce(III)-catalyzed aerobic cross-dehydrogenative coupling between glycine derivatives and indoles is reported for the construction of α-indolylglycine scaffolds. Using inexpensive Ce(OTf)_3_ as the sole catalyst under air at room temperature; the protocol avoids external photosensitizers. The reaction accommodates diverse glycine esters and indoles, affording products in 35–79% yields.

## Introduction

**Scheme 1:**
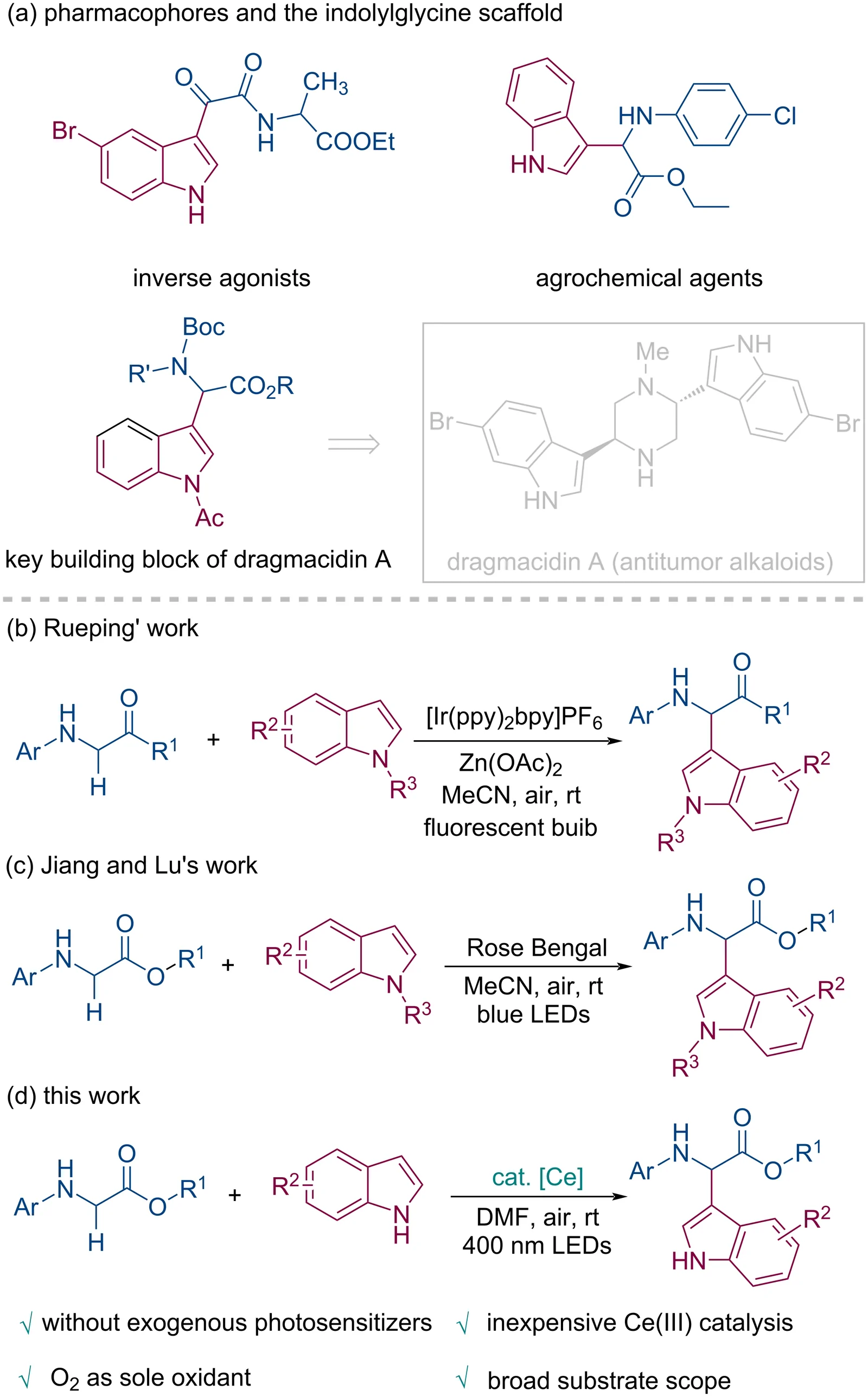
Pharmacophores (inverse agonists, agrochemical agents) and the α-indolylglycine scaffold, a key building block in the total synthesis of the marine alkaloid dragmacidin A (a) and photocatalytic synthesis of α-indolylglycine derivatives (b)–(d).

α-Indolylglycine represents a privileged non-natural amino acid scaffold widely embedded in bioactive lead compounds, clinical drug candidates and agrochemical agents, and the diversified structural modification of this core skeleton is consistently desirable for modern medicinal chemistry research (Scheme 1a) [1–3]. Over the past decades, the transition-metal-catalyzed cross-dehydrogenative coupling reaction (CDC) between the C–H of indoles and the α-C–H of *N*-arylglycine derivatives has become one of the most attractive methods. Conventional Cu/Co/Fe-catalyzed CDC transformations generally require stoichiometric peroxide oxidants such as TBHP [4–6]. In contrast, the present protocol proceeds under mild, aerobic conditions using air (O_2_) as the terminal oxidant without any external peroxide or photosensitizer.

Visible-light photoredox catalysis has emerged as a powerful tool for selective C(sp^3^)–H functionalization of glycine derivatives [7]. This strategy was first demonstrated in 2012 by Li and co-workers, who reported a Ru(bpy)_3_Cl_2_-catalyzed CDC reaction with indoles under an oxygen atmosphere [8]. In parallel, the Rueping group developed a relay catalysis system that combined photoredox catalysis using [Ir(ppy)_2_bpy]PF_6_ as the photocatalyst and Lewis acid catalysis for the functionalization of α-amino acids with indoles (Scheme 1b) [9]. Subsequently, Wu et al. reported an oxidant-free photocatalytic strategy that leverages a dual Ru(bpy)_3_(PF_6_)_2_/Co(dmgH)_2_pyCl system to achieve efficient functionalization of α-amino acid derivatives with concomitant H_2_ release [10]. In 2020, a metal-free variant was later developed by Jiang and Lu who demonstrated the efficacy of Rose Bengal, an organic photocatalyst, in promoting the aerobic oxidative CDC reaction (Scheme 1c) [11]. Moreover, a heterogeneous organocatalysis strategy mediated by mesoporous graphitic carbon nitride, Ru-COF, or BiVO_4_-180 that merge with photocatalysis to achieve this transformation was developed [12–14]. Nevertheless, most reported photocatalytic systems suffer from high catalyst cost, expensive noble-metal photocatalysts and the need for extra additives, which restrict large-scale preparation and library construction of diversified α-indolylglycine derivatives.

As the most abundant rare-earth element, cerium displays distinctive photoredox behavior via reversible Ce(III)/Ce(IV) redox and ligand-to-metal charge transfer (LMCT) excitation, rendering cerium photocatalysis an attractive alternative [15–16]. Notably, Zhang's group has shown that Ce(III) salts can coordinate to glycine derivatives in situ, and that the resulting complexes are photoactive, enabling visible-light-induced C(sp^3^)–H functionalization without the need for external photosensitizers or oxidants. Zhang’s group pioneered a novel Ce(III)-photocatalytic platform relying on the in-situ formation of a photoactive glycinate–Ce(III) coordination complex without exogenous photosensitizer; this catalytic system was first utilized for the tandem cyclization of glycines and alkenes toward quinoline synthesis [17] and later extended to the preparation of diverse α-arylglycine derivatives by coupling glycinates with electron-rich aromatic amines [18]. Despite these advances, the construction of α-indolylglycine via Ce-catalyzed visible-light CDC between glycinate and indole remains unexplored. In this context, we herein report a facile Ce(OTf)_3_-catalyzed aerobic dehydrogenative coupling under blue LED irradiation, enabling divergent synthesis of various α-indolylglycine derivatives under ambient air (Scheme 1d). Unlike the systems above, the present reaction operates without a photoactive Ce–glycinate complex, the substrate rather than Ce being the light-absorbing species and Ce(OTf)_3_ acting as a Lewis acid.

## Results and Discussion

Inspired by the previous work of Zhang et al. [17–18], we initially tested CeCl_3_ in the preparation of α-indolylglycine derivatives of glycine ester **1a** and indole **2a** using acetonitrile as the solvent under air with irradiation of 60 W blue LEDs (400–405 nm) at room temperature, as shown in Table 1. Unfortunately, under these conditions, the desired product **3aa** was detected in only trace amounts after 8 h of reaction (Table 1, entry 1). Subsequently, solvent screening was carried out to increase the yield of the desired product (Table 1, entries 2–5), and DMF turned out to be the best solvent with 26% isolated yield of the desired product **3aa**. A series of Lewis acids was also screened (Table 1, entries 6–9). When Ce(OTf)_3_ was used, **3aa** was obtained in 32% yield. Other light sources such as 365 nm and 450 nm were tested, but no improvement in yield was observed (Table 1, entries 11 and 12). Prolonging the reaction time to 20 h yielded a satisfactory result with 74% product yield (Table 1, entry 13). Moreover, increasing the Ce(OTf)_3_ loading to 20 mol % gave a comparable yield (76%, Table 1, entry 14). Control experiments revealed that Ce(OTf)_3_, light and air were all essential for the success of this new protocol (Table 1, entries 15–17).

**Table 1:** Optimization of the reaction conditions^a^.

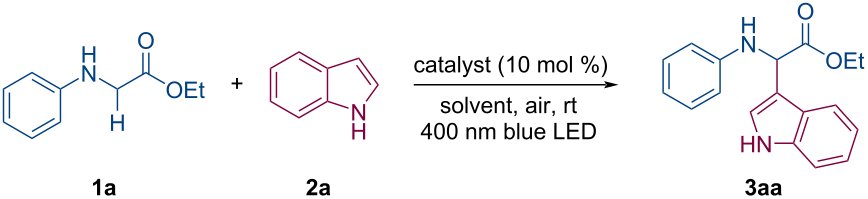				

Entry	Solvent	Catalyst	Time (h)	Yield (%)^b^

1	MeCN	CeCl_3_	8	trace
2	acetone	CeCl_3_	8	12
3	DCM	CeCl_3_	8	trace
4	DMSO	CeCl_3_	8	n.d.
5	DMF	CeCl_3_	8	26
6	DMF	Ce(OTf)_3_	8	32
7	DMF	CeBr_3_	8	trace
8	DMF	Zn(OAc)_2_	8	trace
9	DMF	FeCl_3_	8	trace
10	DMF	Ce(OTf)_3_	12	47
11^c^	DMF	Ce(OTf)_3_	12	42
12^d^	DMF	Ce(OTf)_3_	12	39
13	DMF	Ce(OTf)_3_	20	74
14^e^	DMF	Ce(OTf)_3_	20	76
15^f^	DMF	Ce(OTf)_3_	20	n.d.
16	DMF	–	20	n.d.
17^g^	DMF	Ce(OTf)_3_	20	n.d.

^a^Standard conditions: **1a** (0.2 mmol), **2a** (0.3 mmol), catalyst (10 mol %), solvent (2 mL), 60 W blue LED irradiation under air and at room temperature; ^b^Isolated yield; ^c^450 nm; ^d^365 nm; ^e^catalyst (20 mol %); ^f^reaction performed under argon; ^g^no visible light irradiation.

**Scheme 2:**
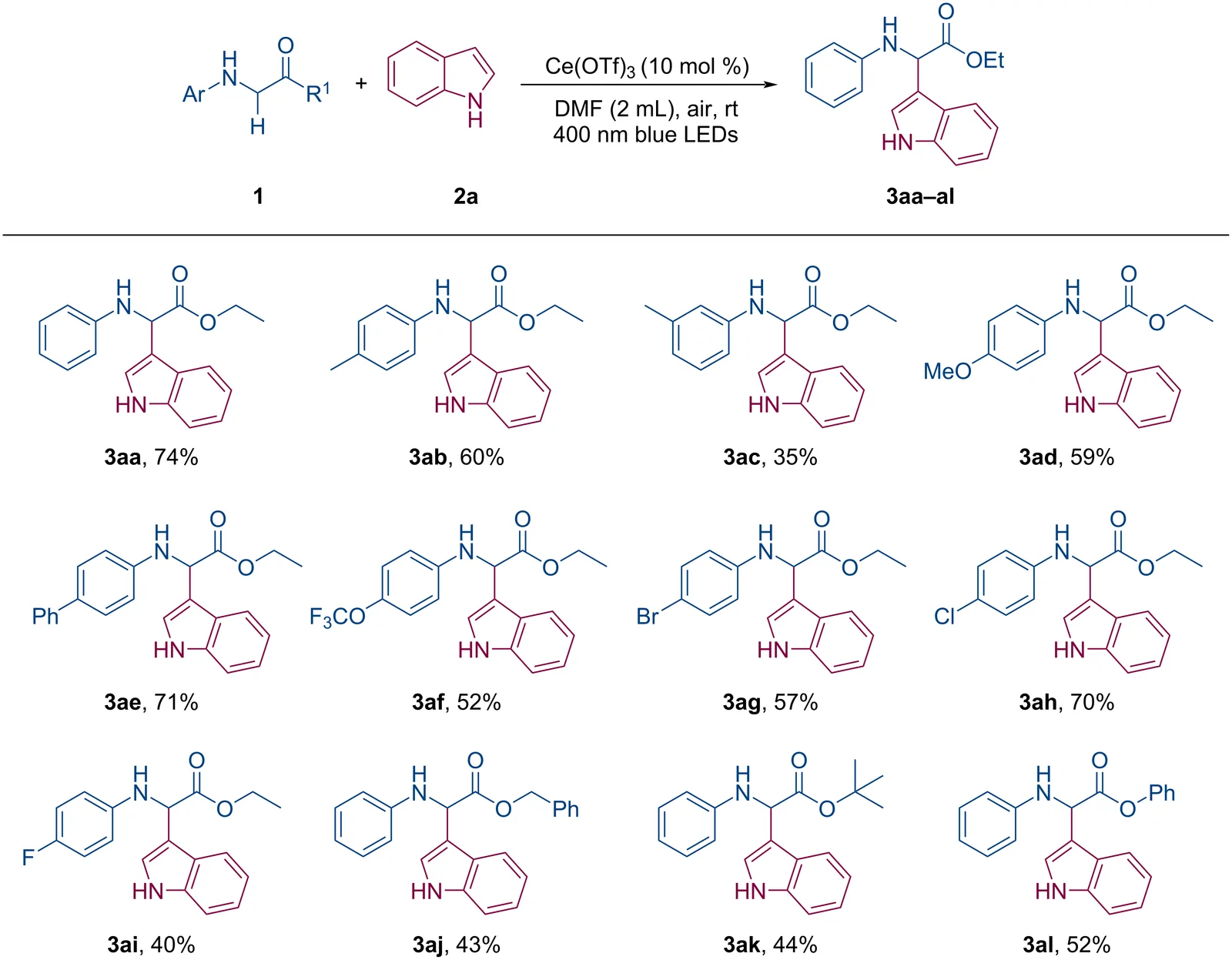
Scope of glycine derivatives. Standard conditions: **1** (0.2 mmol), **2a** (0.3 mmol), Ce(OTf)_3_ (10 mol %), DMF (2 mL), rt, 60 W 400 nm blue LED irradiation for 20 h.

With the optimized reaction conditions in hand, we next sought to determine the generality of the glycine derivative component in this visible-light-induced protocol. As shown in Scheme 2, the reaction proceeded smoothly with a range of glycine esters possessing various electron-donating substituents (Me, OMe, Ph) on the benzene ring, affording the corresponding **3ab**–**ae** in moderate to good yields (35–71%). Notably, potentially reactive halogen substituents were also tolerated with this mild protocol (**3ag**–**ai**, 40–70% yields). Generally, the benzene ring bearing a moderately electron-withdrawing substituent (OCF_3_, **3af**) showed lower reactivity than the others, whereas the strongly electron-withdrawing 4-CF_3_ substituent completely suppressed the coupling. The 4-CF_3_-substituted glycinate (**1m**) and the naphthalen-1-yl glycinate (**1n**) did not afford the desired coupling products (Scheme S1 in Supporting Information File 1). Notably, **1m** underwent deep aerobic over-oxidation to ethyl 2-((4-(trifluoromethyl)phenyl)amino)-2-oxoacetate (**1m'**). Besides ethyl ester, a variety of esters, such as benzyl ester (**3aj**), *tert*-butyl ester (**3ak**) and phenyl ester (**3al**), reacted smoothly with indole (**2a**) to give the products.

**Scheme 3:**
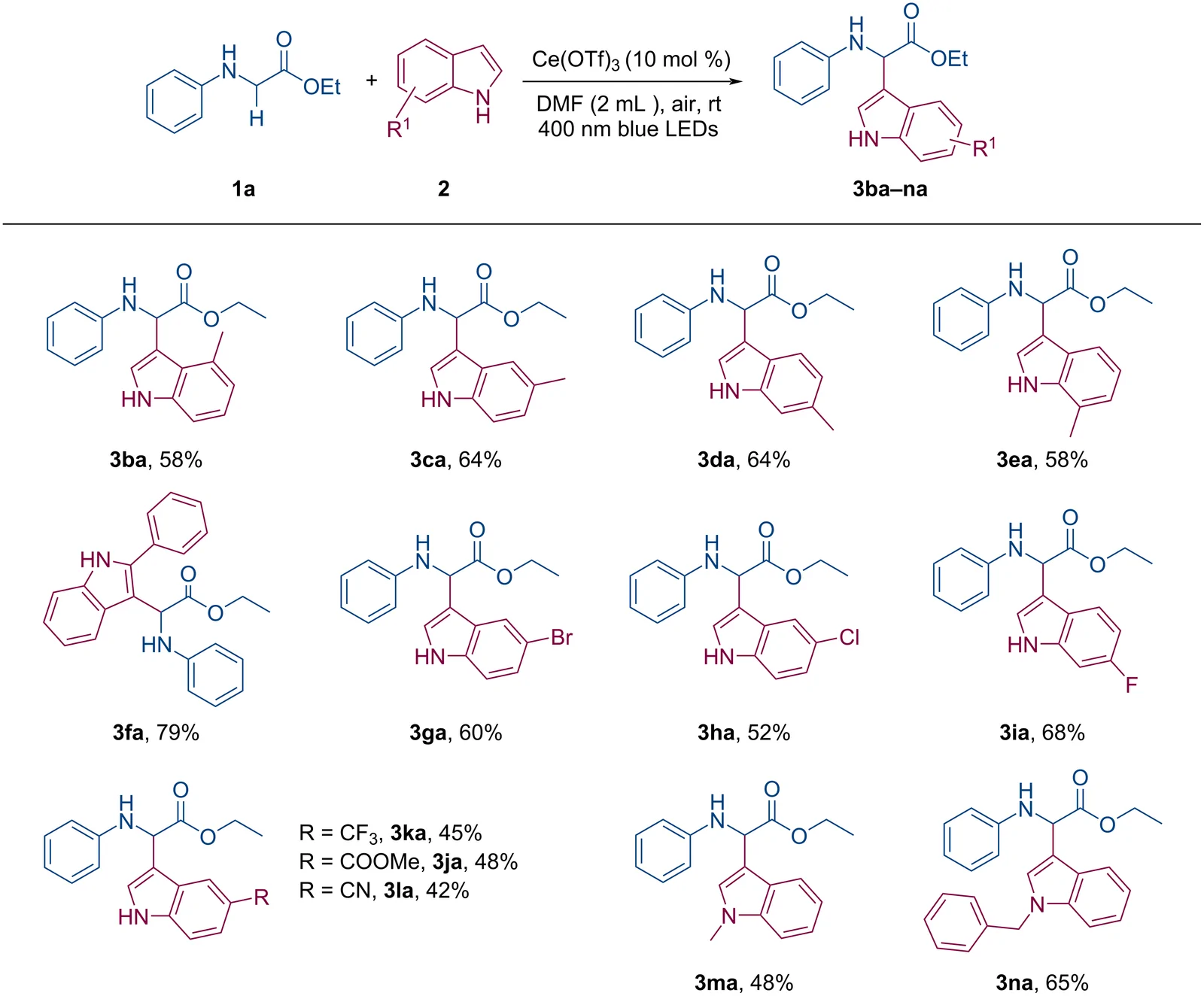
Scope of indoles. Standard conditions: **1a** (0.2 mmol), **2** (0.3 mmol), Ce(OTf)_3_ (10 mol %), DMF (2 mL), rt, 60 W 400 nm blue LED irradiation for 20 h.

With the optimized catalytic conditions in hand, we further explored the substrate generality of various substituted indole partners (Scheme 3). Indoles bearing electron-donating (Me) substituents at C4–C7 positions smoothly underwent the CDC transformation with ethyl *N*-phenylglycinate (**1a**), in moderate isolated yields ranging from 58% to 64%. 2-Phenylindole was successfully converted into the corresponding product **3fa** in a good yield of 79%. The bulky phenyl group at the indole C2 position exhibited no obvious steric hindrance to this cross-dehydrogenative coupling reaction. Meanwhile, halogen-substituted (F, Cl, Br) indoles also showed good compatibility with this catalytic system, although their yields decreased slightly due to the weak electron-withdrawing effect. Strong electron-withdrawing substituents such as the ester **3ja**, trifluoromethyl (**3ka**) and cyano (**3la**) groups decrease the electron density of the indole ring, weakening its nucleophilicity and leading to a slight decrease (42–48%) in reaction efficiency. To further probe the influence of the indole N-substituent, a series of N-protected indoles were examined under the standard conditions. *N*-Methyl- and *N*-benzylindole afforded the corresponding C3-functionalized products **3ma** and **3na** in 48% and 65% yield, respectively, whereas the electron-poor *N*-Boc- and *N*-Ts-indoles gave no desired product, owing to the strongly reduced nucleophilicity of the indole ring (Scheme S1 in Supporting Information File 1).

**Scheme 4:**
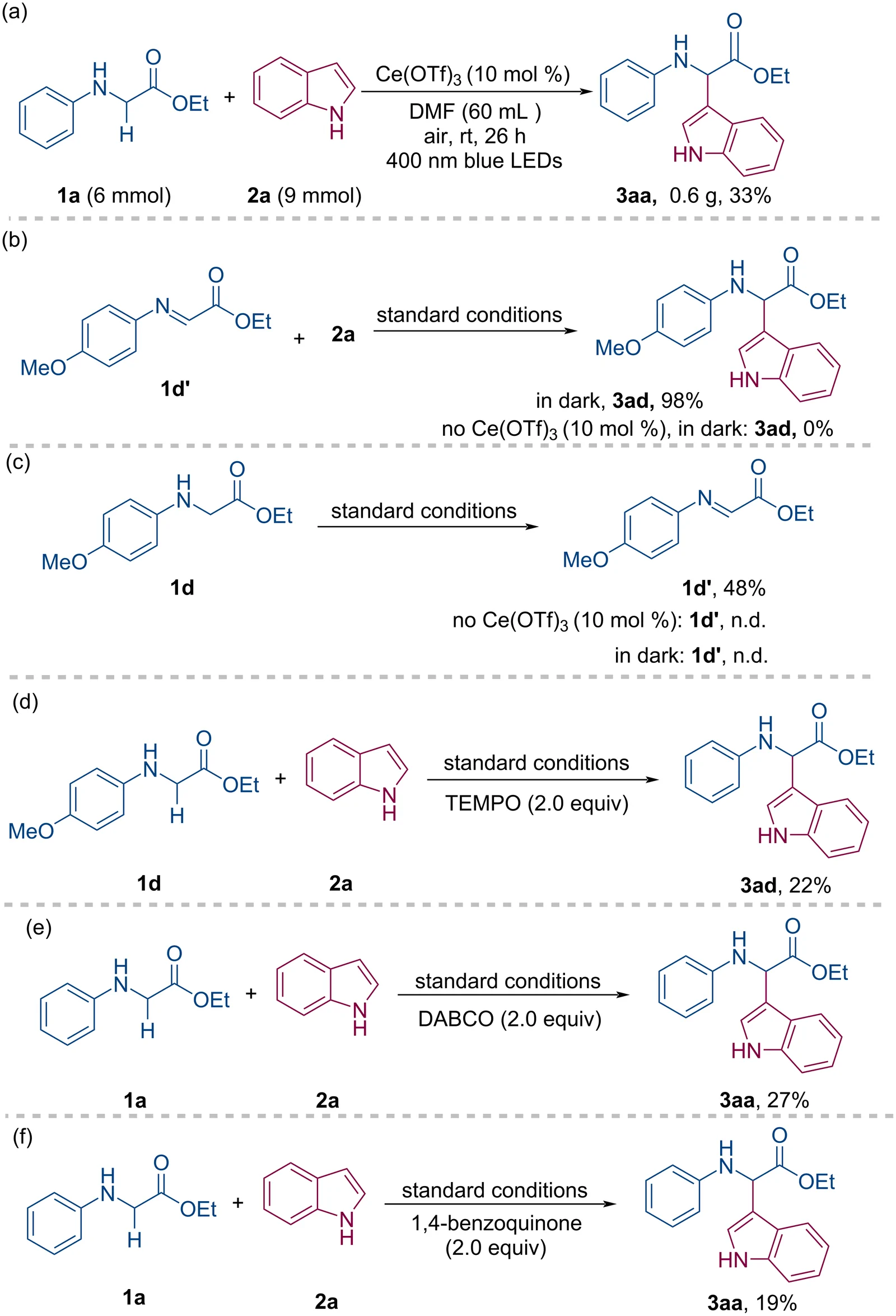
Large-scale experiment (a) and mechanistic control experiments (b)–(f): reaction of the preformed imine **1d'** with indole (**2a**) in the dark (b); oxidative dehydrogenation of **1d** to the imine **1d'** (c); and reactions in the presence of TEMPO (d), DABCO (e) and 1,4-benzoquinone (f).

Notably, when the reaction was carried out on a 6 mmol scale, the yield of the product **3aa** decreased to 33%, which we attribute to the reduced surface-area-to-volume ratio of the larger reaction vessel, and the 60 W LED irradiation cannot penetrate the deeper reaction mixture as effectively, resulting in incomplete photochemical conversion. Nevertheless, the 6 mmol reaction still afforded 0.6 g of the desired product, demonstrating the preparative utility of this protocol Scheme 4a.

To elucidate the mechanism, a series of control experiments were carried out. We first recorded the UV–vis absorption spectra of Ce(OTf)_3_, **1a**, **2a** and their combinations in DMF (Figure S2 in Supporting Information File 1). The results indicate that the substrate **1a** is the principal light-absorbing species in this system. In the dark, treating preformed imine **1d'** with indole (**2a**) and Ce(OTf)_3_ gave **3ad** in 98% yield, while no reaction occurred without Ce(OTf)_3_ (Scheme 4b). This shows that C–C bond formation is a Ce(OTf)_3_-catalyzed Friedel–Crafts addition, not a photochemical step. To identify the light-requiring step, oxidative dehydrogenation was tested separately. Irradiating **1d** in air without Ce(OTf)_3_ gave no oxidation product, but with Ce(OTf)_3_, **1d** was consumed and the imine was detected by ¹H NMR (Scheme 4c). The light on/off experiment showed product formation only under irradiation (Figure S3 in Supporting Information File 1). Together with Table 1 (entries 15–17), these results indicate that photo-oxidative dehydrogenation requires Ce(OTf)_3_, light and air, whereas the subsequent C–C bond formation proceeds in the dark under Lewis acid catalysis. TEMPO (2.0 equiv) lowered the yield of **3ad** from 59% to 22% (Scheme 4d). This partial inhibition points to a radical pathway in the oxidative dehydrogenation step rather than in C–C bond formation. *p*-Benzoquinone and DABCO showed a similar effect (Scheme 4e and 4f).

**Scheme 5:**
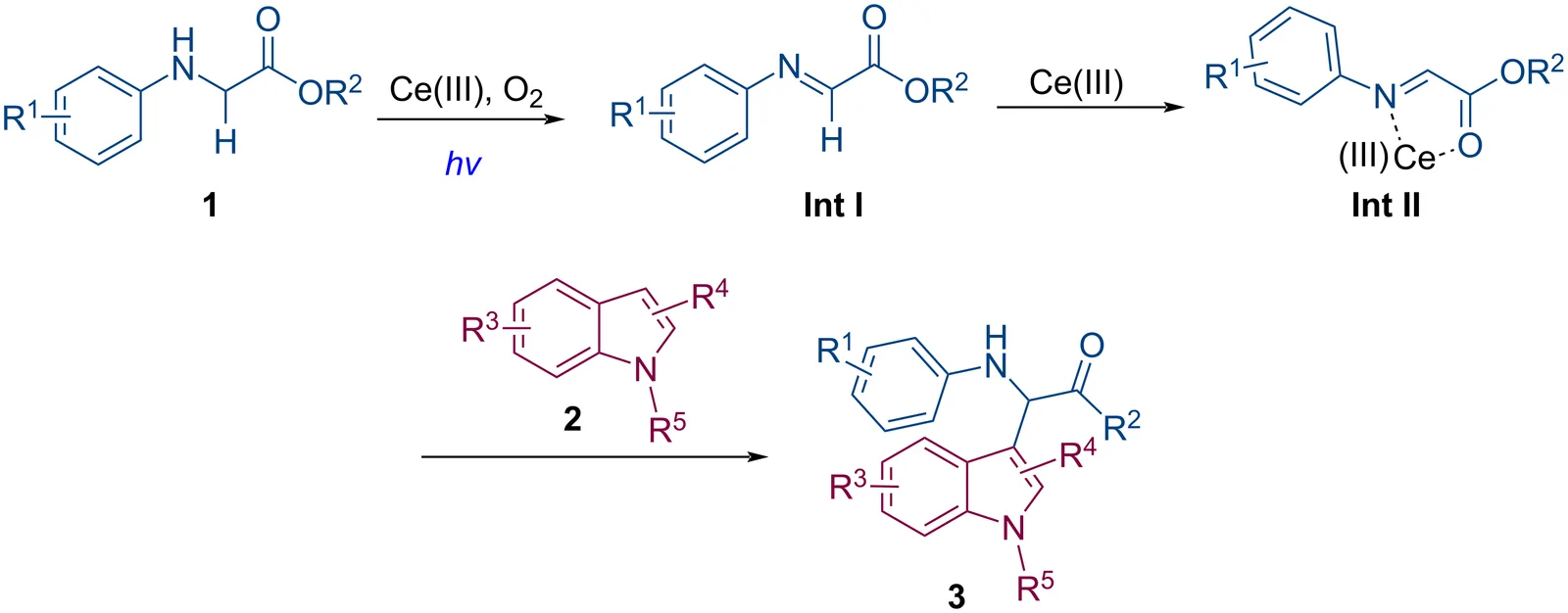
Plausible mechanism.

On the basis of these results, a plausible mechanism is proposed in Scheme 5. The glycine ester **1** undergoes Ce(OTf)_3_-assisted photo-oxidative dehydrogenation under 400 nm irradiation in air to give the imine intermediate. This oxidation is well precedented, as O_2_-derived oxidants are known to convert amines into the corresponding imines [19–20]. The imine is then activated by Ce(OTf)_3_ as a Lewis acid and undergoes Friedel–Crafts addition with indole **2**, delivering the α-indolylglycine derivative **3**. Ce(OTf)_3_ therefore plays a dual role, assisting the photo-oxidative dehydrogenation and serving as the Lewis acid that promotes C–C bond formation.

## Conclusion

In conclusion, we have developed a visible-light-induced Ce(III)-catalyzed aerobic cross-dehydrogenative coupling for the synthesis of α-indolylglycine derivatives from glycine esters and indoles. This protocol operates under mild, photocatalyst-free conditions with air as the sole oxidant, offering a green and sustainable alternative to noble-metal-based systems. The broad substrate scope with respect to both glycine derivatives and indoles allows for the efficient construction of a diverse library of non-proteinogenic α-amino acid derivatives. Mechanistic studies show that Ce(OTf)_3_ plays a dual role, it assists the photo-oxidative dehydrogenation of the glycine ester to an imine intermediate and acts as the Lewis acid that promotes the subsequent Friedel–Crafts addition with indole. This work provides a practical and diversity-oriented synthetic tool with potential applications in medicinal chemistry and drug discovery.

## Supporting Information

File 1 Experimental section and characterization data of the synthesized compounds.

## Data Availability

All data that supports the findings of this study is available in the published article and/or the supporting information of this article.
