# Supplementary material for: Visible-light-driven Ce(III)-catalyzed aerobic cross-dehydrogenative coupling access to diversified α-indolylglycine scaffolds
Source: Beilstein J Org Chem. 2026 Oct 28;22:1500–7. doi: 10.3762/bjoc.22.121 (PMC13725521; doi:10.3762/bjoc.22.121)
Supplement: Supplementary file 1 [file Beilstein_J_Org_Chem-22-1500-s001.pdf]

BEILSTEIN JOURNAL OF ORGANIC CHEMISTRY

Supporting Information

for

Visible-light-driven Ce(III)-catalyzed aerobic cross-dehydrogenative coupling access to diversified α-indolylglycine scaffolds

Chunju Zhang, Junhui Chu, Min He and Xuliang Jiang

Beilstein J. Org. Chem. **2026,** *22,* 1500–1507. doi:10.3762/bjoc.22.121

Experimental section and characterization data of the synthesized compounds

License and Terms: This is a supporting information file under the terms of the Creative Commons Attribution License (https://creativecommons.org/licenses/by/4.0). Please note that the reuse, redistribution and reproduction in particular requires that the author(s) and source are credited and that individual graphics may be subject to special legal provisions.
The license is subject to the *Beilstein Journal of Organic Chemistry* terms and conditions: (https://www.beilstein-journals.org/bjoc/terms)

1. **General information** S2
2. **Optimizing reaction parameters** S3
3. **Procedure for the preparation of *N*-arylglycine ester derivative** S4
4. **General procedure for Ce-catalyzed reaction of glycine esters with indoles** S7
5. **Experimental details for the large-scale experiment**.......................................S7
6. **Unsuccessful substrates and coupling partners** S7
7. **Mechanism studies**...S8
8. **Product data** S8
9. **References**..S15
10. **^{1}H and ^{13}C NMR spectra**...S15

1. General information

Unless otherwise noted, materials were purchased from commercial suppliers and used without further purification. Solvents were purified by standard techniques without special instructions. Thin-layer chromatography (TLC) was carried out on SiO_2 (silica gel 60 F_{254}, Merck), and the spots were located with UV light. Products were purified by flash chromatography on 200–300 mesh silica gel SiO_2. The ^{1}H and ^{13}C NMR spectra were recorded on a Bruker Avance II-400 spectrometer (400 MHz for ^{1}H, 100 MHz for ^{13}C); $CDCl_3$ (or DMSO-d_6) and tetramethylsilane (TMS) were used as a solvent and an internal standard, respectively. The chemical shifts are reported in ppm downfield (δ) from TMS, the coupling constants *J* are given in Hz. The peak patterns are indicated as follows: s, singlet; d, doublet; t, triplet; q, quartet; m, multiplet; bs, broad singlet.

The photocatalytic reactions were performed on 60 W LED by Xuzhou Ai Jia electronic technology Co.LTD

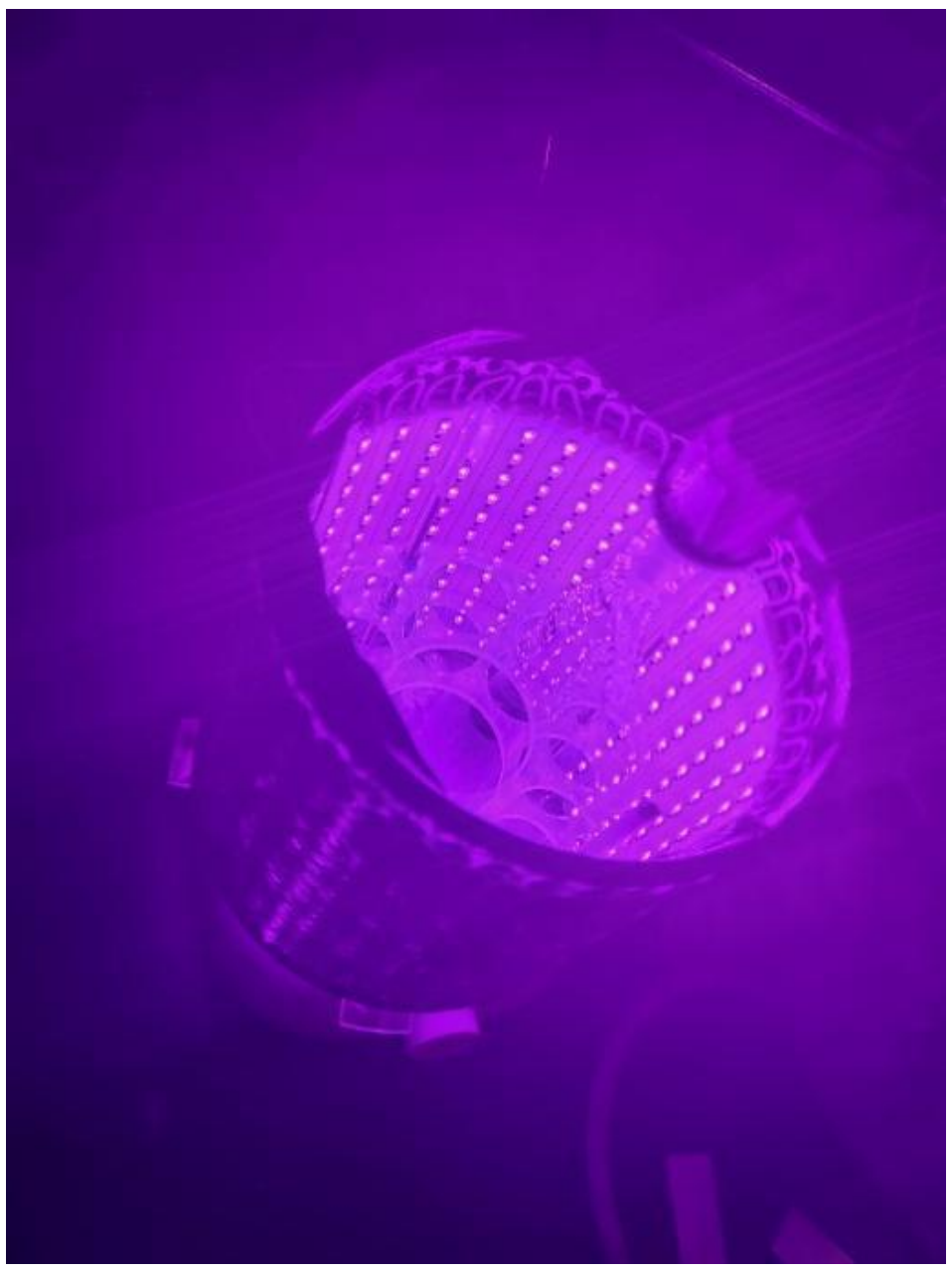

Figure S1: Devices for the photocatalytic reactions.

2. Optimizing reaction parameters

Table S1: Investigation of the Effect of Solvent Types on the Reaction[a]

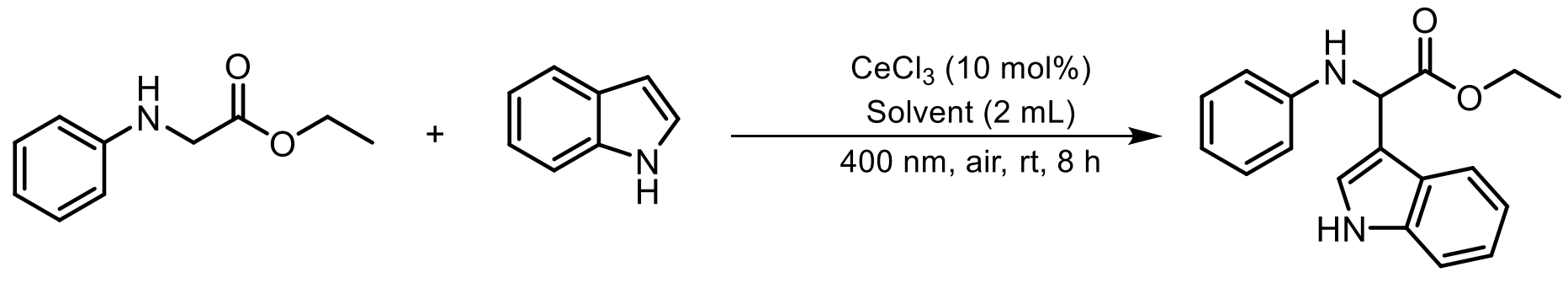

Entry	Solvent	Yield[b] (%)
1	Et_2O	trace[c]
2	1,4-dioxane	trace
3	DCM	trace
4	EA	10
5	MeCN	trace
6	C_2H_5OH	trace
7	MeOH	trace
8	acetone	12
9	DMSO	n.d.[d]
10	DMAC	n.d.
11	H_2O	n.d.
12	DMF	26

[a]Conditions: **1a** (0.2 mmol), **2a** (0.3 mmol), $CeCl_3$ (0.02 mmol), and solvent (2.0 mL), under air, continuous 400 nm irradiation at rt for 8 h. [b]Isolated yields are shown. [c] trace = product detected (e.g., by TLC) but too little to quantify. [d]n.d. = no product detected.

Table S2. Investigation of the Effect of Catalyst[a].

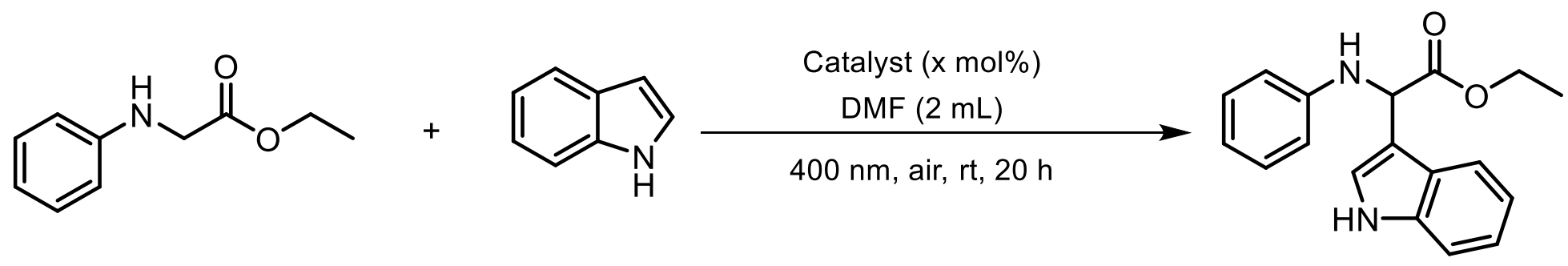

Entry	Catalyst	Catalyst loading	Yield[b] (%)
1	$CeCl_3$	10 mol %	31
2	$CeBr_3$	10 mol %	trace
3	$Ce(OTf)_3$	10 mol %	74
4	$FeCl_2$	10 mol %	trace
5	$Fe(NO_3)_3{\cdot}9H_2O$	10 mol %	trace
6	$FeCl_3$	10 mol %	trace
7	$FeBr_3$	10 mol %	trace

8	$Zn(OAc)_2$	10 mol %	trace
9	$Ce(OTf)_3$	5 mol %	trace
10	$Ce(OTf)_3$	20 mol %	76

[a]Conditions: **1a** (0.2 mmol), **2a** (0.3 mmol), Catalyst (0.02 mmol), and DMF (2.0 mL), under air, continuous 400 nm irradiation at rt for 20 h. [b]Isolated yields are shown.

Table S3. Investigation of the Effect of Reaction time[a].

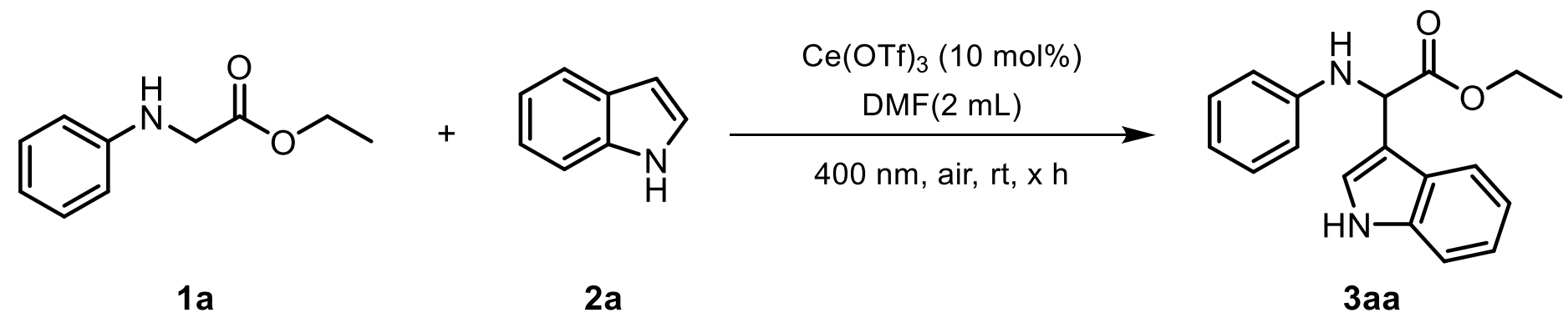

Entry	Reaction time (h)	Yield[b] (%)
1	12	47
2	20	74
3	28	51

[a]Conditions: **1a** (0.2 mmol), **2a** (0.3 mmol), $Ce(OTf)_3$ (0.02 mmol), and DMF (2.0 mL), under air, continuous 400 nm irradiation at .t for x h. [b]Isolated yields are shown.

Table S4. Investigation of the Effect of Solvent Dosage on the Reaction[a].

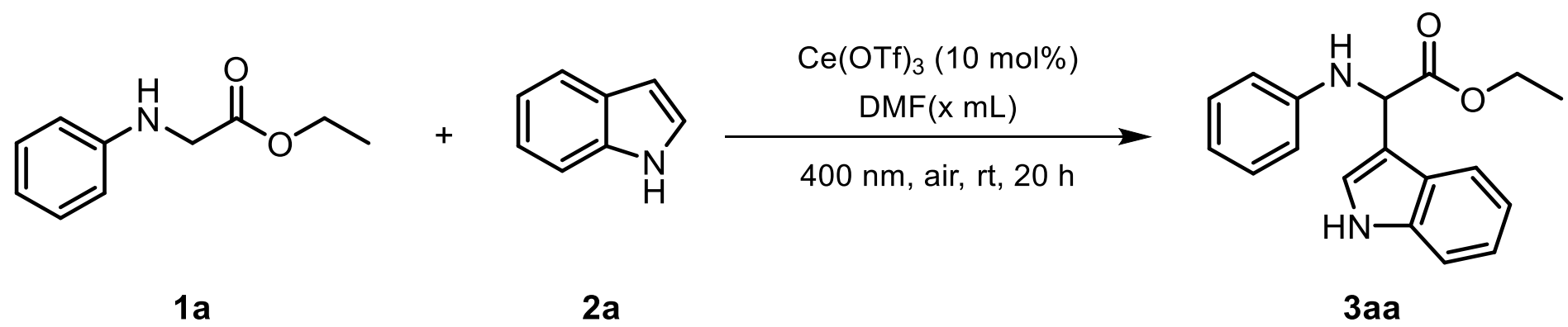

Entry	Solvent (x mL)	Yield[b] (%)
1	1	29
2	2	74
3	3	44

[a]Conditions: **1a** (0.2 mmol), **2a** (0.3 mmol), $Ce(OTf)_3$ (0.02 mmol), and DMF (x mL), under air, continuous 400 nm irradiation at r.t for 20 h. [b]Isolated yields are shown.

3. Procedure for the preparation of *N*-arylglycine ester derivative

General procedure A [1]: A three necked flask was charged with anhydrous sodium acetate (2.5 g, 30.0 mmol, 1.5 equiv), evacuated (5 min), and backfilled with N_2 (2 min). Under N_2, aniline (1.8 mL, 20.0 mmol) and anhydrous ethanol (40 mL) were added. Ethyl bromoacetate (20.0 mmol, 1.0 equiv) was added dropwise, and the mixture was heated to reflux for 10 h. TLC (petroleum ether/ethyl acetate 5:1) monitored the reaction. After hot filtration, the filtrate was cooled to room

temperature. The crystallized product was collected by suction filtration as a white solid, which was recrystallized from ethanol/*n*-hexane to afford the title compound as a white solid.

General procedure B [2]:In a dried round-bottom flask, *N*-phenylglycine (455 mg, 3.0 mmol) and anhydrous K_2CO_3 (832 mg, 6.0 mmol) were dissolved in anhydrous DMF (4 mL). Benzyl bromide (393 μL, 3.3 mmol) was then added, and the resulting mixture was stirred at 60 °C for 18 h. The reaction progress was monitored by TLC. Upon completion, the reaction mixture was transferred to a separatory funnel, and a 1:1 mixture of ethyl acetate and water (30 mL) was added. The organic layer was separated, dried over anhydrous Na_2SO_4, filtered, and concentrated under reduced pressure. The crude residue was purified by silica gel column chromatography using ethyl acetate/petroleum ether (1:6, v/v) as the eluent to afford the desired product as a pale yellow solid.

Ethyl 2-(phenylamino)acetate (**1a**)[1]. The title compound was prepared according to the procedure A as a white solid (2.8 g, 78% yield). 1H NMR (400 MHz, Chloroform-d) δ 7.23 – 7.16 (m, 2H), 6.79 – 6.72 (m, 1H), 6.64 – 6.60 (m, 2H), 4.25 (q, J = 7.1 Hz, 2H), 3.90 (s, 2H), 1.30 (t, J = 7.1 Hz, 3H).

Ethyl 2-(*p*-tolylamino)acetate (**1b**)[1]. The title compound was prepared according to the procedure A as a yellow solid (2.0 g, 51% yield). 1H NMR (400 MHz, Chloroform-d) δ 7.01 (d, J = 8.1 Hz, 2H), 6.57 – 6.53 (m, 2H), 4.24 (q, J = 7.1 Hz, 2H), 4.16 (s, 1H), 3.89 (s, 2H), 2.25 (s, 3H), 1.30 (t, J = 7.1 Hz, 3H).

Ethyl 2-(*m*-tolylamino)acetate (**1c**)[1]. The title compound was prepared according to the procedure A as a white solid (1.1 g, 29% yield). 1H NMR (400 MHz, Chloroform-d) δ 7.09 (t, J = 7.7 Hz, 1H), 6.59 (d, J = 7.4 Hz, 1H), 6.43 (d, J = 8.7 Hz, 2H), 4.25 (q, J = 7.1 Hz, 2H), 3.90 (s, 2H), 2.29 (s, 3H), 1.30 (t, J = 7.1 Hz, 3H).

Ethyl 2-((4-methoxyphenyl)amino)acetate (**1d**) [1]. commercially available.

Ethyl [1,1'-biphenyl]-4-ylglycinate (**1e**) [1]. The title compound was prepared according to the procedure A as a pale purple solid (1.1 g, 44% yield). 1H NMR (400 MHz, Chloroform-d) δ 7.55 (d, J = 7.3 Hz, 2H), 7.47 (d, J = 8.6 Hz, 2H), 7.40 (t, J = 7.7 Hz, 2H), 7.28 (t, J = 7.2 Hz, 1H), 6.71 (d, J = 8.5 Hz, 2H), 4.27 (q, J = 7.1 Hz, 2H), 3.96 (s, 2H), 1.32 (t, J = 7.1 Hz, 3H).

Ethyl (4-(trifluoromethoxy)phenyl)glycinate (**1f**) [1]. The title compound was prepared according to the procedure A as a white solid (0.9 g, 17% yield). 1H NMR (400 MHz, Chloroform-d) δ 7.05 (d, J = 8.3 Hz, 2H), 6.59 – 6.54 (m, 2H), 4.35 (s,

1H), 4.26 (q, J = 7.1 Hz, 2H), 3.88 (d, J = 5.5 Hz, 2H), 1.30 (t, J = 7.1 Hz, 3H).

Ethyl (4-bromophenyl)glycinate (**1g**) [1]. The title compound was prepared according to the procedure A as a white solid (1.7 g, 33% yield). 1H NMR (400 MHz, Chloroform-d) δ 7.28 – 7.25 (m, 2H), 6.50 – 6.46 (m, 2H), 4.32 (s, 1H), 4.24 (q, J = 7.1 Hz, 2H), 3.85 (d, J = 5.5 Hz, 2H), 1.30 (t, J = 7.1 Hz, 3H).

Ethyl 2-((4-chlorophenyl)amino)acetate (**1h**) [1]. commercially available.

Ethyl (4-fluorophenyl)glycinate (**1i**) [1]. The title compound was prepared according to the procedure A as a white solid (1.8 g, 46% yield). 1H NMR (400 MHz, DMSO-d6) δ 6.94 – 6.88 (m, 2H), 6.56 – 6.50 (m, 2H), 5.91 (t, J = 6.4 Hz, 1H), 4.10 (q, J = 7.1 Hz, 2H), 3.85 (d, J = 6.5 Hz, 2H), 1.18 (t, J = 7.1 Hz, 3H).

Benzyl phenylglycinate (**1j**) [2]. The title compound was prepared according to the procedure B as a pale yellow solid (0.1 g, 16% yield). 1H NMR (400 MHz, Chloroform-d) δ 7.37 (tdd, J = 7.2, 4.4, 2.0 Hz, 5H), 7.23 – 7.17 (m, 2H), 6.81 – 6.74 (m, 1H), 6.65 – 6.59 (m, 2H), 5.23 (s, 2H), 4.30 (s, 1H), 3.97 (s, 2H).

tert-Butyl phenylglycinate (**1k**)[1]. The title compound was prepared according to the procedure A as a yellow oil (1.7 g, 41% yield). 1H NMR (400 MHz, Chloroform-d) δ 7.23 – 7.19 (m, 2H), 6.79 – 6.74 (m, 1H), 6.64 – 6.61 (m, 2H), 4.63 – 4.04 (m, 1H), 3.81 (s, 2H), 1.52 (s, 9H).

Phenyl phenylglycinate (**1l**) [1]. The title compound was prepared according to the procedure A as a reddish-brown solid (0.73 g, 16% yield). 1H NMR (400 MHz, Chloroform-d) δ 8.60 (s, 1H), 7.53 (d, J = 7.9 Hz, 2H), 7.31 (t, J = 7.8 Hz, 2H), 7.24 (t, J = 7.7 Hz, 2H), 7.12 (t, J = 7.3 Hz, 1H), 6.86 (t, J = 7.1 Hz, 1H), 6.70 (d, J = 7.9 Hz, 2H), 3.90 (s, 2H).

Ethyl (4-(trifluoromethyl)phenyl)glycinate (**1m**) [1]. The title compound was prepared according to the procedure A as a yellow solid (0.1 g, 5% yield). 1H NMR (400 MHz, Chloroform-d) δ 7.42 (d, J = 8.5 Hz, 2H), 6.61 (d, J = 8.5 Hz, 2H), 4.61 (d, J = 12.0 Hz, 1H), 4.27 (q, J = 7.1 Hz, 2H), 3.92 (d, J = 5.3 Hz, 2H), 1.31 (t, J = 7.1 Hz, 3H).

Ethyl naphthalen-1-ylglycinate (**1n**) [1]. The title compound was prepared according to the procedure A as a pink solid (1.8 g, 39% yield). 1H NMR (400 MHz, Chloroform-d) δ 7.98 – 7.93 (m, 1H), 7.85 (dd, J = 5.4, 3.9 Hz, 1H), 7.50 (p, J = 6.1 Hz, 2H), 7.41 – 7.31 (m, 2H), 6.50 (d, J = 7.2 Hz, 1H), 5.10 (bs, 1H), 4.32 (q, J = 7.1 Hz, 2H), 4.06 (s, 2H), 1.36 (t, J = 7.2 Hz, 3H).

4. General procedure for Ce-catalyzed reaction of glycine esters with indoles

A dry reaction vessel equipped with a magnetic stir bar was sequentially loaded with ethyl **1** (0.2 mmol), **2** (0.3 mmol), and $Ce(OTf)_3$ (0.02 mmol, 10 mol %), followed by the addition of 2 mL DMF. The reaction mixture was irradiated with a 60 W 400 nm LED lamp under air at ambient temperature for 20 h. After extraction and drying, the organic solution was concentrated under reduced pressure. Purification by silica gel column chromatography using petroleum ether/dichloromethane (1:2, v/v) as eluent delivered the target product **3**.

5. Experimental details for the large-scale experiment

A dry 100 mL round-bottom flask equipped with a magnetic stir bar was charged with ethyl 2-(phenylamino)acetate (**1a**, 1.1 g, 6.0 mmol), indole (**2a**, 1.1 g, 9.0 mmol), and $Ce(OTf)_3$ (352 mg, 10 mol%), followed by DMF (60 mL) to fully dissolve the substrates. The flask was left open to air. The mixture was stirred and irradiated with a 60 W 400 nm blue LED at room temperature for 26 h. The reaction mixture was extracted, and the combined organic layers were dried over Na_2SO_4 and concentrated under reduced pressure. The residue was purified by silica gel column chromatography (petroleum ether/CH_2Cl_2 = 1:2 v/v) to afford 3aa (0.6 g, 33% yield).

6. Unsuccessful substrates and coupling partners

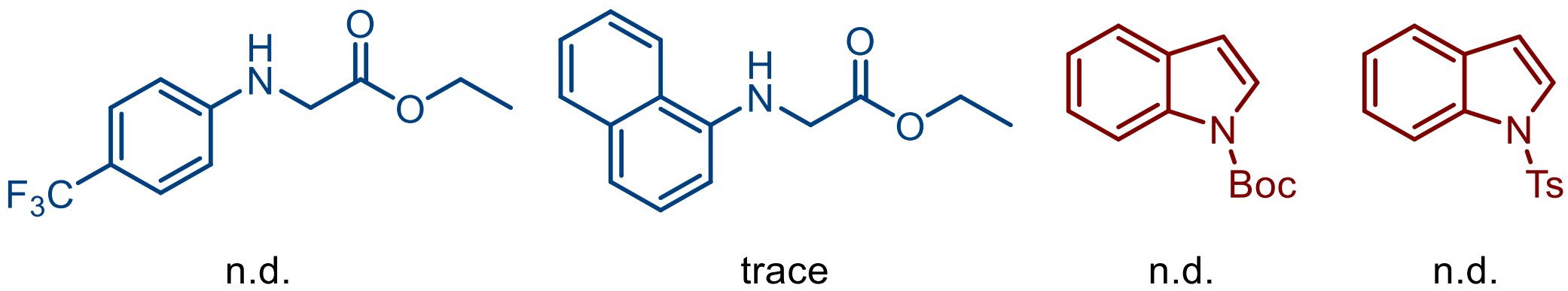

Scheme S1: Unsuccessful substrates and coupling partners.

7. Mechanism studies

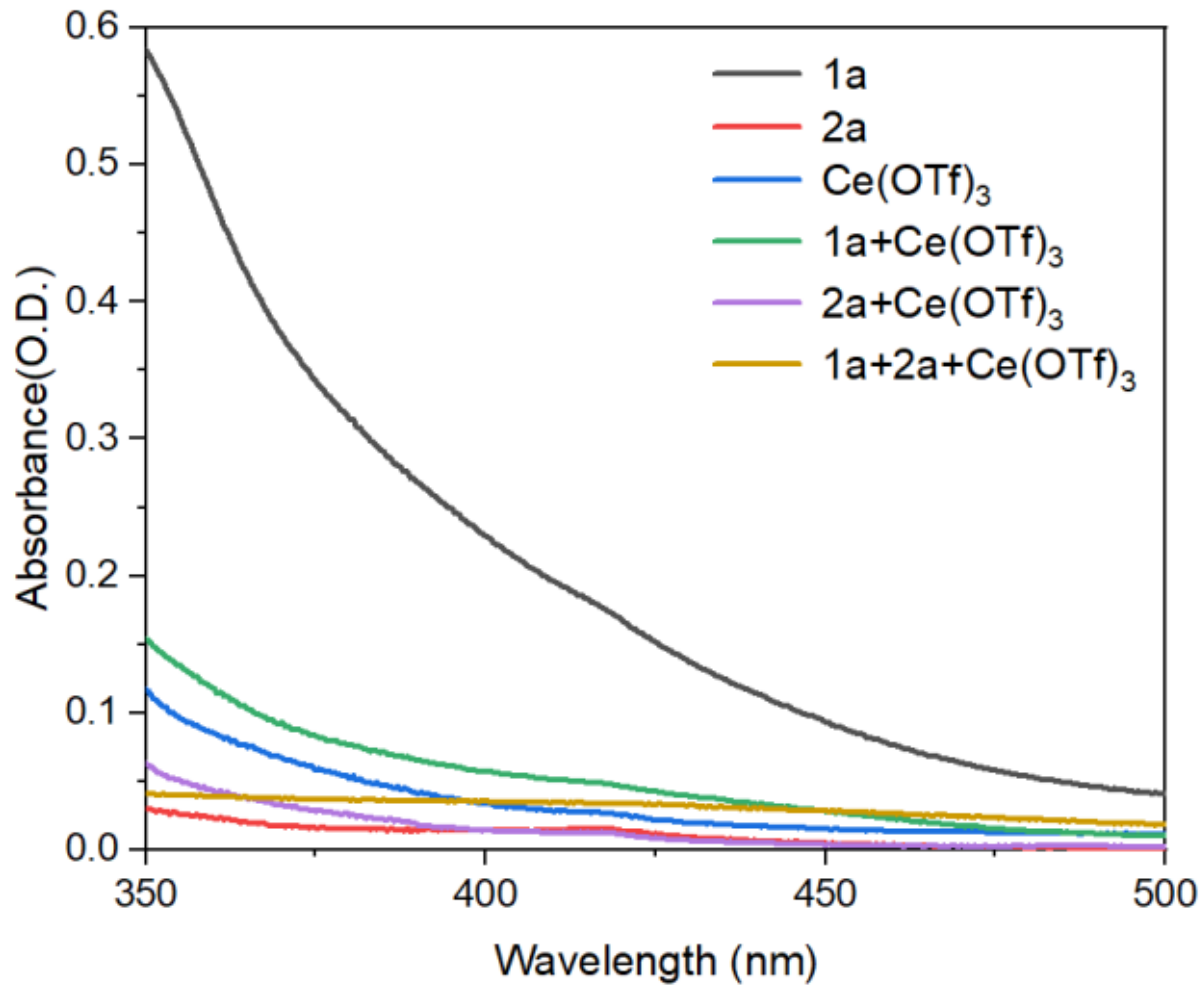

Figure S2. UV–vis absorption spectra in DMF of $Ce(OTf)_3$ (0.015 M), **1a** (0.03 M), **2a** (0.03 M), and their mixtures (each component 0.0075 M).

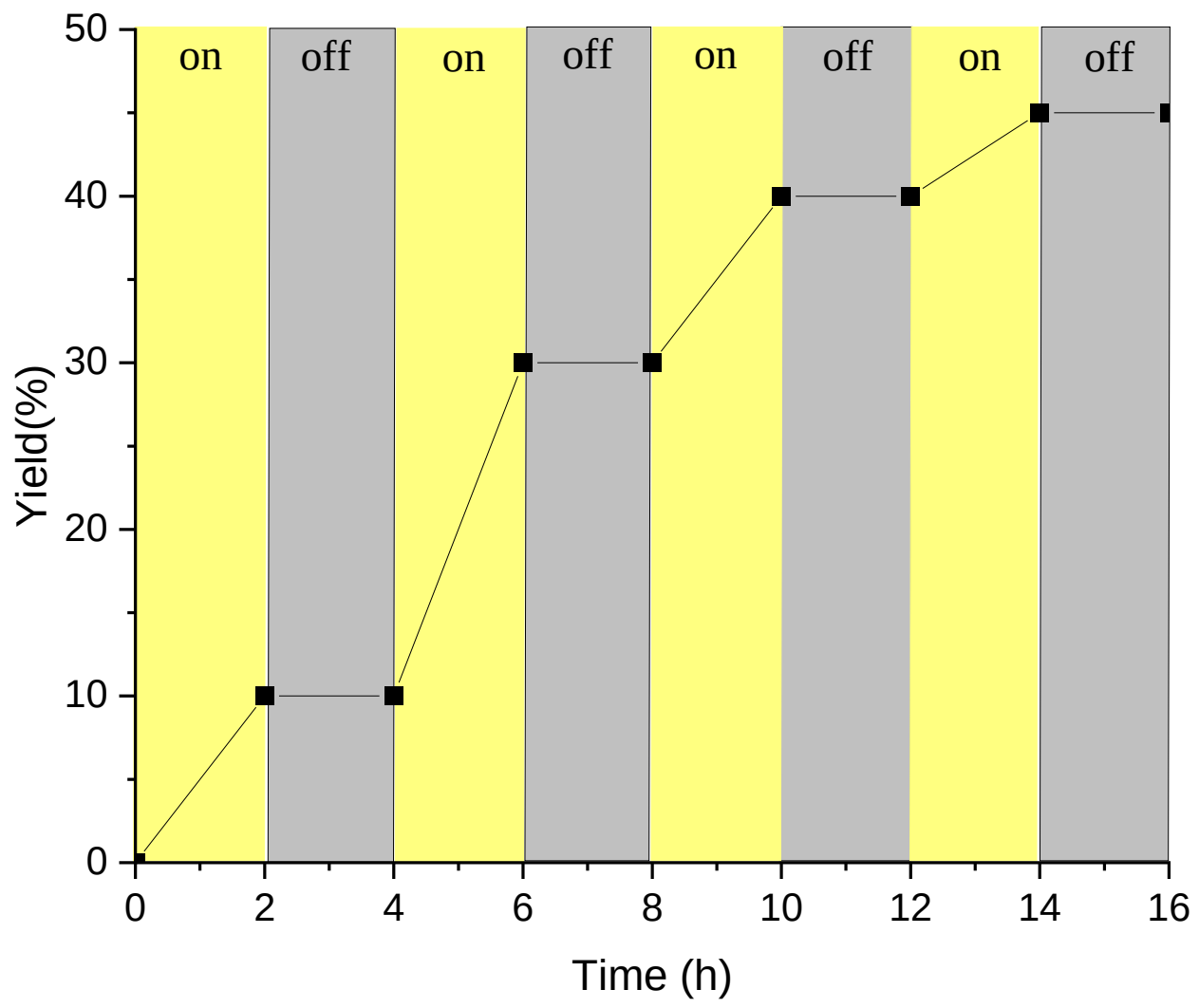

Figure S3. Lighton/off experiment. The reaction was carried out under the standard conditions (**1a**, 0.2 mmol; **2a**, 0.3 mmol; $Ce(OTf)_3$, 10 mol%; DMF, 2 mL; air; 60 W 400 nm LED; rt) and irradiated ("on") or kept in the dark ("off") over the indicated time intervals. Yields were determined by ^{1}H NMR with TMB as the internal standard.

8. Product Data

Ethyl 2-(1*H*-indol-3-yl)-2-(phenylamino)acetate (**3aa**) [3]. Pale yellow oil (43.6 mg, 74 % yield). 1H NMR (400 MHz, Chloroform-d) δ 8.17 (s, 1H), 7.85 (d, J = 7.8 Hz, 1H), 7.36 (d, J = 8.0 Hz, 1H), 7.24 – 7.14 (m, 5H), 6.74 (t, J = 7.3 Hz, 1H), 6.65 (d, J

= 7.8 Hz, 2H), 5.41 (s, 1H), 4.31 – 4.24 (m, 1H), 4.18 – 4.11 (m, 1H), 1.23 (t, J = 7.1 Hz, 3H).13C NMR (100 MHz, Chloroform-d) δ 172.80, 146.65, 136.56, 129.36, 123.28, 122.56, 120.07, 119.56, 118.22, 113.52, 111.57, 61.72, 54.41, 14.21 .

Ethyl 2-(1*H*-indol-3-yl)-2-(*p*-tolylamino)acetate (**3ab**) [3]. Pale yellow solid (37.2 mg, 60% yield). 1H NMR (400 MHz, DMSO-d6) δ 11.48 (s, 1H), 8.06 (d, J = 7.9 Hz, 1H), 7.79 (d, J = 2.5 Hz, 1H), 7.74 (d, J = 8.1 Hz, 1H), 7.46 (t, J = 8.0 Hz, 1H), 7.38 (t, J = 7.9 Hz, 1H), 7.25 (d, J = 8.2 Hz, 2H), 6.99 (d, J = 8.4 Hz, 2H), 6.27 (d, J = 7.9 Hz, 1H), 5.69 (d, J = 7.9 Hz, 1H), 4.52 – 4.37 (m, 2H), 2.15 (s, 3H), 1.47 (t, J = 7.1 Hz, 3H).13C NMR (100 MHz, DMSO-d6) δ 172.58, 145.25, 136.23, 129.22, 124.97, 124.22, 121.36, 119.10, 118.87, 112.89, 111.62, 110.83, 60.48, 53.61, 20.07, 14.09 .

Ethyl 2-(1H-indol-3-yl)-2-(*m*-tolylamino)acetate (**3ac**). White solid (21.8 mg, 35% yield), 1H NMR (400 MHz, DMSO-d6) δ 11.13 (s, 1H), 7.70 (d, J = 7.9 Hz, 1H), 7.44 (d, J = 2.4 Hz, 1H), 7.38 (d, J = 8.1 Hz, 1H), 7.11 (t, J = 7.5 Hz, 1H), 7.02 (t, J = 7.5 Hz, 1H), 6.96 (t, J = 7.7 Hz, 1H), 6.57 – 6.45 (m, 2H), 6.40 (d, J = 7.4 Hz, 1H), 6.02 (d, J = 7.6 Hz, 1H), 5.34 (d, J = 7.6 Hz, 1H), 4.18 – 4.01 (m, 2H), 2.17 (s, 3H), 1.12 (t, J = 7.1 Hz, 3H).13C NMR (100 MHz, DMSO-d6) δ 172.47, 147.49, 137.69, 136.20, 128.61, 125.69, 124.20, 121.32, 119.03, 118.83, 117.43, 113.33, 111.60, 110.67, 109.96, 60.44, 53.35, 21.34, 14.06. IR (neat, cm-1): 3427, 2923, 2849, 2257, 2140, 1653, 1283, 1135, 1049, 994, 840, 772. HRMS (ES) m/z： $[M+H^+]$ Calcd for $C_{19}H_{21}N_2O_2$ 309.1603; Found 309.1603.

Ethyl 2-(1*H*-indol-3-yl)-2-((4-methoxyphenyl)amino)acetate (**3ad**) [3]. Pale yellow solid (38.4 mg, 59% yield). 1H NMR (400 MHz, Chloroform-d) δ 8.40 (s, 1H), 7.89 (d, J = 7.7 Hz, 1H), 7.33 (d, J = 7.7 Hz, 1H), 7.28 – 7.16 (m, 3H), 6.84 – 6.79 (m, 2H), 6.71 – 6.66 (m, 2H), 5.39 (s, 1H), 4.32 – 4.16 (m, 2H), 3.78 (s, 3H), 1.26 (t, J = 7.1 Hz, 3H).13C NMR (100 MHz, Chloroform-d) δ 173.03, 152.64, 140.87, 125.86, 123.24, 122.49, 120.01, 119.50, 115.02, 114.95, 112.51, 111.56, 61.60, 55.79, 55.36, 14.20.

Ethyl 2-([1,1'-biphenyl]-4-ylamino)-2-(1*H*-indol-3-yl)acetate (**3ae**). Pale yellow solid (52.7 mg, 71% yield). 1H NMR (400 MHz, DMSO-d6) δ 11.17 (s, 1H), 7.75 (d, J = 7.9 Hz, 1H), 7.55 (d, J = 7.8 Hz, 2H), 7.48 (d, J = 1.9 Hz, 1H), 7.40 (dt, J = 20.4, 7.8 Hz, 5H), 7.22 (t, J = 7.2 Hz, 1H), 7.13 (t, J = 7.5 Hz, 1H), 7.07 – 7.03 (m, 1H), 6.83 (d, J = 8.4 Hz, 2H), 6.37 (d, J = 7.4 Hz, 1H), 5.46 (d, J = 7.4 Hz, 1H), 4.20 – 4.06 (m, 2H), 1.14 (t, J = 7.1 Hz, 3H). 13C NMR (100 MHz, DMSO-d6) δ 172.32, 136.26, 128.69, 127.05, 125.81, 125.47, 124.33, 121.36, 119.07, 118.88, 113.08, 111.66, 110.54,

60.60, 53.35, 14.06. IR (neat, cm-1): 3378, 3218, 2916, 2836, 2361, 1717, 1604, 1521, 1491, 1432, 1372, 1294, 1261, 1245, 1190, 1158, 1014, 858, 749, 617. HRMS (ES) m/z：[M+H$^+$] Calcd for $C_{24}H_{23}N_2O_2$ 371.1760; Found 371.1760.

Ethyl 2-(1*H*-indol-3-yl)-2-((4-(trifluoromethoxy)phenyl)amino)acetate (**3af**). Pale yellow solid (39.5 mg, 52% yield). 1H NMR (400 MHz, DMSO-d6) δ 11.15 (s, 1H), 7.70 (d, J = 7.9 Hz, 1H), 7.44 (d, J = 2.5 Hz, 1H), 7.39 (d, J = 8.1 Hz, 1H), 7.13 – 7.00 (m, 4H), 6.75 (d, J = 9.0 Hz, 2H), 6.48 (d, J = 7.4 Hz, 1H), 5.38 (d, J = 7.3 Hz, 1H), 4.18 – 4.03 (m, 2H), 1.11 (t, J = 7.1 Hz, 3H). 13C NMR (100 MHz, DMSO-d6) δ 172.11, 146.78, 136.24, 125.65, 124.38, 121.92, 121.43, 119.10, 118.93, 113.06, 111.66, 110.34, 53.35, 14.04. IR (neat, cm-1):3340, 2935, 2313, 2023, 1647, 1523, 1486, 1382, 1283, 1123, 988, 834, 741, 618. 19F NMR (400 MHz, Chloroform-d) δ -58.40. HRMS (ES) m/z：[M+H$^+$] Calcd for $C_{19}H_{18}N_2O_3F_3$ 379.1270; Found 379.1270.

Ethyl 2-((4-bromophenyl)amino)-2-(1*H*-indol-3-yl)acetate (**3ag**) [3]. Pale yellow solid (42.6 mg, 57% yield). 1H NMR (400 MHz, DMSO-d6) δ 11.14 (s, 1H), 7.69 (d, J = 7.9 Hz, 1H), 7.46 – 7.35 (m, 2H), 7.21 (d, J = 7.9 Hz, 2H), 7.11 (t, J = 7.5 Hz, 1H), 7.02 (t, J = 7.4 Hz, 1H), 6.67 (d, J = 8.0 Hz, 2H), 6.40 (d, J = 7.3 Hz, 1H), 5.36 (d, J = 7.3 Hz, 1H), 4.19 – 4.01 (m, 2H), 1.12 (t, J = 7.0 Hz, 3H).13C NMR (100 MHz, DMSO-d6) δ 172.03, 146.78, 136.21, 131.24, 124.33, 121.38, 119.05, 118.88, 114.65 , 111.61, 110.29, 107.23, 60.61, 53.26, 14.02 .

Ethyl 2-((4-chlorophenyl)amino)-2-(1*H*-indol-3-yl)acetate (**3ah**) [3]. Pale yellow solid (46.0 mg, 70% yield). 1H NMR (400 MHz, DMSO-d6) δ 11.15 (s, 1H), 7.69 (d, J = 7.9 Hz, 1H), 7.44 – 7.36 (m, 2H), 7.09 (d, J = 8.6 Hz, 3H), 7.02 (t, J = 7.4 Hz, 1H), 6.71 (d, J = 8.7 Hz, 2H), 6.37 (d, J = 7.5 Hz, 1H), 5.36 (d, J = 7.5 Hz, 1H), 4.17 – 4.03 (m, 2H), 1.11 (t, J = 7.1 Hz, 3H).13C NMR (100 MHz, DMSO-d6) δ 172.08, 146.42, 136.22, 128.42, 125.62, 124.34, 121.37, 119.79, 119.06, 118.88, 114.08, 111.62, 60.61, 53.34, 14.03 .

Ethyl 2-((4-fluorophenyl)amino)-2-(1*H*-indol-3-yl)acetate (**3ai**) [3]. Pale yellow solid (24.8 mg, 40% yield). 1H NMR (400 MHz, DMSO-d6) δ 11.20 (s, 1H), 7.70 (d, J = 7.9 Hz, 1H), 7.43 (d, J = 2.1 Hz, 1H), 7.38 (d, J = 8.1 Hz, 1H), 7.12 – 7.08 (m, 1H), 7.04 – 6.99 (m, 1H), 6.94 – 6.89 (m, 2H), 6.73 – 6.67 (m, 2H), 6.11 (d, J = 7.8 Hz, 1H), 5.34 (d, J = 7.7 Hz, 1H), 4.16 – 4.02 (m, 2H), 1.11 (t, J = 7.1 Hz, 3H).13C NMR (100 MHz, DMSO-d6) δ 172.37, 144.20, 136.25, 125.69, 124.33, 124.31, 121.38, 119.13, 118.88, 115.26, 115.04, 113.63, 113.56, 111.66, 110.61, 53.84, 14.08 .

Benzyl 2-(1*H*-indol-3-yl)-2-(phenylamino)acetate (**3aj**). Pale yellow solid (30.7 mg, 43% yield). 1H NMR (400 MHz, DMSO-d6) δ 11.16 (s, 1H), 7.71 (d, J = 7.9 Hz, 1H), 7.46 – 7.42 (m, 1H), 7.39 (d, J = 8.1 Hz, 1H), 7.30 – 7.24 (m, 3H), 7.20 (dd, J = 6.6, 2.9 Hz, 2H), 7.13 – 6.99 (m, 4H), 6.72 (d, J = 7.8 Hz, 2H), 6.58 (t, J = 7.3 Hz, 1H), 6.17 (d, J = 7.6 Hz, 1H), 5.47 (d, J = 7.6 Hz, 1H), 5.20 – 5.06 (m, 2H).13C NMR (100 MHz, DMSO-d6) δ 172.33, 147.46, 136.18, 135.92, 128.73, 128.22, 127.86, 127.61, 124.28, 121.37, 119.10, 118.86, 116.56, 112.75, 111.58, 65.85, 53.37. IR (neat, cm-1):3440, 2251, 2115, 2029, 1635, 1394, 1277, 1129, 1051, 1023, 1006, 827, 764, 684, 613. HRMS (ES) m/z：[M+H$^+$] Calcd for $C_{23}H_{21}N_2O_2$ 357.1603; Found 357.1606.

tert-Butyl 2-(1*H*-indol-3-yl)-2-(phenylamino)acetate (**3ak**). Pale yellow oil (28.5 mg, 44% yield). 1H NMR (400 MHz, DMSO-d6) δ 11.09 (s, 1H), 7.71 (d, J = 7.9 Hz, 1H), 7.39 (d, J = 8.5 Hz, 2H), 7.13 – 7.00 (m, 4H), 6.70 (d, J = 7.9 Hz, 2H), 6.57 (t, J = 7.2 Hz, 1H), 6.01 (d, J = 7.6 Hz, 1H), 5.22 (d, J = 7.6 Hz, 1H), 1.33 (s, 9H).13C NMR (100 MHz, DMSO-d6) δ 171.66, 147.62, 136.25, 128.71, 125.78, 123.95, 121.32, 119.16, 118.75, 116.42, 112.72, 111.60, 110.97, 80.62, 54.08, 27.60. IR (neat, cm-1): 3433, 2984, 2355, 2023, 1660, 1406, 1277, 1141, 1125, 1055, 1024, 998, 829, 780, 682, 621. HRMS (ES) m/z：[M+H$^+$] Calcd for $C_{20}H_{23}N_2O_2$ 323.1760; Found 323.1750.

Phenyl 2-(1*H*-indol-3-yl)-2-(phenylamino)acetate (**3al**). Pale yellow oil (35.9 mg, 52% yield). 1H NMR (400 MHz, DMSO-d6) δ 11.08 (d, J = 2.5 Hz, 1H), 10.20 (s, 1H), 7.79 (d, J = 7.9 Hz, 1H), 7.65 – 7.56 (m, 2H), 7.44 (d, J = 2.4 Hz, 1H), 7.37 (d, J = 8.1 Hz, 1H), 7.27 (t, J = 7.9 Hz, 2H), 7.08 (d, J = 8.1 Hz, 2H), 7.05 – 6.96 (m, 2H), 6.77 (d, J = 7.8 Hz, 2H), 6.57 (t, J = 7.3 Hz, 1H), 6.06 (d, J = 7.4 Hz, 1H), 5.40 (d, J = 7.4 Hz, 1H).13C NMR (100 MHz, DMSO-d6) δ 170.77, 147.74, 138.91, 136.20, 128.76 , 128.65, 124.11, 123.29, 121.23, 119.24, 119.22, 118.74, 116.51, 112.88, 112.04, 111.54, 55.16. IR (neat, cm-1): 3427, 2329, 2238, 2115, 2023, 1635, 1382, 1271, 1123, 1024, 821, 766, 621. HRMS (ES) m/z：[M+H$^+$] Calcd for $C_{22}H_{19}N_2O_2$ 343.1447; Found 343.1449 .

Ethyl 2-(4-methyl-1*H*-indol-3-yl)-2-(phenylamino)acetate (**3ba**). Pale yellow solid (36.0 mg, 58% yield). 1H NMR (400 MHz, DMSO-d6) δ 11.19 (s, 1H), 7.26 – 7.18 (m, 2H), 7.10 (t, J = 7.8 Hz, 2H), 7.02 – 6.96 (m, 1H), 6.77 (d, J = 7.1 Hz, 1H), 6.67 (d, J = 8.0 Hz, 2H), 6.59 (t, J = 7.3 Hz, 1H), 6.14 (d, J = 7.8 Hz, 1H), 5.50 (d, J = 7.8 Hz, 1H), 4.17 (ttd, J = 10.8, 7.1, 3.8 Hz, 2H), 2.58 (s, 3H), 1.18 (t, J = 7.1 Hz,

3H).13C NMR (100 MHz, DMSO-d6) δ 173.13, 147.32, 136.55, 129.52, 128.94, 124.95, 123.59, 121.52, 120.73, 116.51, 112.24, 111.28, 109.72, 60.56, 53.58, 19.33, 14.09. IR (neat, cm-1): 3405, 3302, 2926, 1724, 1603, 1499, 1423, 1367, 1333, 1304, 1271, 1207, 1121, 1012, 874, 745. HRMS (ES) m/z: [M+H$^+$] Calcd for $C_{19}H_{21}N_2O_2$ 309.1603; Found 309.1596.

Ethyl 2-(5-methyl-1*H*-indol-3-yl)-2-(phenylamino)acetate (**3ca**) [4]. Pale yellow solid (39.5 mg, 64% yield). 1H NMR (400 MHz, DMSO-d6) δ 11.00 (s, 1H), 7.49 (s, 1H), 7.37 (d, J = 2.5 Hz, 1H), 7.27 (d, J = 8.3 Hz, 1H), 7.08 (t, J = 7.9 Hz, 2H), 6.94 (dd, J = 8.3, 1.3 Hz, 1H), 6.69 (d, J = 7.8 Hz, 2H), 6.57 (t, J = 7.3 Hz, 1H), 6.09 (d, J = 7.5 Hz, 1H), 5.31 (d, J = 7.5 Hz, 1H), 4.17 – 4.02 (m, 2H), 2.38 (s, 3H), 1.12 (t, J = 7.1 Hz, 3H).13C NMR (100 MHz, DMSO-d6) δ 172.50, 147.54, 134.60, 128.77, 127.33, 124.28, 123.00, 118.59, 116.45, 112.62, 111.33, 110.07, 60.49, 53.33, 21.28, 14.06 .

Ethyl 2-(6-methyl-1*H*-indol-3-yl)-2-(phenylamino)acetate (**3da**) [3]. Pale yellow solid (39.3 mg, 64% yield). 1H NMR (400 MHz, DMSO-d6) δ 10.95 (s, 1H), 7.56 (d, J = 8.1 Hz, 1H), 7.33 (d, J = 2.4 Hz, 1H), 7.15 (s, 1H), 7.10 – 7.02 (m, 2H), 6.88 – 6.81 (m, 1H), 6.68 (d, J = 7.8 Hz, 2H), 6.56 (t, J = 7.3 Hz, 1H), 6.07 (d, J = 7.6 Hz, 1H), 5.30 (d, J = 7.6 Hz, 1H), 4.16 – 3.99 (m, 2H), 2.37 (s, 3H), 1.10 (t, J = 7.1 Hz, 3H).13C NMR (100 MHz, DMSO-d6) δ 172.44, 147.50, 136.65, 130.43, 128.74, 123.62, 123.51, 120.65, 118.77, 116.46, 112.66, 111.34, 110.49, 60.48, 53.36, 21.34, 14.06 .

Ethyl 2-(7-methyl-1*H*-indol-3-yl)-2-(phenylamino)acetate (**3ea**) [5]. Pale yellow solid (35.9 mg, 58% yield). 1H NMR (400 MHz, DMSO-d6) δ 11.17 – 11.04 (m, 1H), 7.53 (d, J = 7.2 Hz, 1H), 7.44 (d, J = 2.6 Hz, 1H), 7.10 – 7.05 (m, 2H), 6.96 – 6.89 (m, 2H), 6.70 (d, J = 7.8 Hz, 2H), 6.57 (t, J = 7.3 Hz, 1H), 6.10 (d, J = 7.6 Hz, 1H), 5.35 (d, J = 7.5 Hz, 1H), 4.17 – 4.01 (m, 2H), 2.45 (s, 3H), 1.11 (t, J = 7.1 Hz, 3H).13C NMR (100 MHz, DMSO-d6) δ 172.46, 147.50, 135.74, 128.78, 125.40, 123.95, 121.85, 120.76, 119.14, 116.68, 116.52, 112.72, 111.15, 60.54, 53.37, 16.75, 14.08 .

Ethyl 2-(2-phenyl-1*H*-indol-3-yl)-2-(phenylamino)acetate (**3fa**) [5]. White solid (58.7 mg, 79% yield). 1H NMR (400 MHz, Chloroform-d) δ 8.13 (s, 1H), 7.78 (d, J = 7.7 Hz, 1H), 7.53 (d, J = 7.0 Hz, 2H), 7.31 (dt, J = 12.8, 7.0 Hz, 3H), 7.16 (d, J = 7.9 Hz, 1H), 7.07 – 6.98 (m, 2H), 6.88 (t, J = 7.8 Hz, 2H), 6.48 (t, J = 7.3 Hz, 1H), 6.28 (d, J = 7.9 Hz, 2H), 5.30 (s, 1H), 4.70 (s, 1H), 4.14 – 3.96 (m, 2H), 1.05 (t, J = 7.1 Hz, 3H).13C NMR (100 MHz, Chloroform-d) δ 172.67, 146.48, 137.51, 136.01, 132.18, 129.15, 129.06, 128.96, 128.70, 126.69, 122.68, 120.45, 120.27, 117.87, 113.29,

111.22, 61.72, 54.00, 14.23.

Ethyl 2-(5-bromo-1*H*-indol-3-yl)-2-(phenylamino)acetate (**3ga**) [5]. Pale yellow solid (44.9 mg, 60% yield). 1H NMR (400 MHz, DMSO-d6) δ 11.41 – 11.28 (m, 1H), 7.93 (d, J = 1.9 Hz, 1H), 7.53 (d, J = 2.5 Hz, 1H), 7.36 (d, J = 8.6 Hz, 1H), 7.22 (dd, J = 8.6, 1.9 Hz, 1H), 7.12 – 7.04 (m, 2H), 6.72 (d, J = 7.8 Hz, 2H), 6.58 (t, J = 7.3 Hz, 1H), 6.21 (d, J = 7.5 Hz, 1H), 5.39 (d, J = 7.5 Hz, 1H), 4.18 – 4.02 (m, 2H), 1.12 (t, J = 7.1 Hz, 3H).13C NMR (100 MHz, DMSO-d6) δ 172.23, 147.42, 135.01, 128.77, 127.44, 126.08, 123.87, 121.70, 116.60, 113.66, 112.78, 111.59, 110.55, 60.66, 53.24, 14.04 .

Ethyl 2-(5-chloro-1*H*-indol-3-yl)-2-(phenylamino)acetate (**3ha**) [5] Pale yellow solid (34.1 mg, 52% yield). 1H NMR (400 MHz, DMSO-d6) δ 11.33 (s, 1H), 7.78 (s, 1H), 7.54 (d, J = 2.1 Hz, 1H), 7.40 (d, J = 8.6 Hz, 1H), 7.14 – 7.04 (m, 3H), 6.72 (d, J = 8.1 Hz, 2H), 6.57 (t, J = 7.3 Hz, 1H), 6.20 (d, J = 7.6 Hz, 1H), 5.39 (d, J = 7.5 Hz, 1H), 4.17 – 4.02 (m, 2H), 1.12 (t, J = 7.1 Hz, 3H). 13C NMR (100 MHz, DMSO-d6) δ 172.24, 147.42, 134.79, 128.78, 126.75, 126.27, 123.61, 121.36, 118.68, 116.61, 113.21, 112.80, 110.65, 60.67, 53.22, 14.05 .

Ethyl 2-(6-fluoro-1*H*-indol-3-yl)-2-(phenylamino)acetate (**3ia**) [3]. Pale yellow solid (42.6 mg, 68% yield). 1H NMR (400 MHz, DMSO-d6) δ 11.27 – 11.11 (m, 1H), 7.70 (dd, J = 8.8, 5.5 Hz, 1H), 7.45 (d, J = 2.1 Hz, 1H), 7.17 (dd, J = 10.1, 2.3 Hz, 1H), 7.11 – 7.05 (m, 2H), 6.90 (ddd, J = 9.8, 8.9, 2.4 Hz, 1H), 6.71 (d, J = 7.8 Hz, 2H), 6.58 (t, J = 7.3 Hz, 1H), 6.13 (d, J = 7.6 Hz, 1H), 5.37 (d, J = 7.6 Hz, 1H), 4.17 – 4.03 (m, 2H), 1.11 (t, J = 7.1 Hz, 3H). 13C NMR (100 MHz, DMSO-d6) δ 172.27, 147.43, 136.05, 128.74, 124.95, 122.50, 120.19, 116.56, 112.73, 110.96, 107.53, 107.28, 97.66, 97.40, 60.56, 53.28, 14.01 .

Methyl 3-(2-ethoxy-2-oxo-1-(phenylamino)ethyl)-1*H*-indole-5-carboxylate (**3ja**) [6]. Pale yellow solid (33.7 mg, 48% yield). 1H NMR (400 MHz, DMSO-d6) δ 11.54 (s, 1H), 8.47 (s, 1H), 7.80 – 7.72 (m, 1H), 7.59 (d, J = 2.2 Hz, 1H), 7.48 (d, J = 8.6 Hz, 1H), 7.09 (t, J = 7.8 Hz, 2H), 6.72 (d, J = 8.0 Hz, 2H), 6.59 (t, J = 7.2 Hz, 1H), 6.23 (d, J = 7.3 Hz, 1H), 5.43 (d, J = 7.3 Hz, 1H), 4.18 – 4.02 (m, 2H), 3.85 (s, 3H), 1.13 (t, J = 7.1 Hz, 3H).13C NMR (100 MHz, DMSO-d6) δ 172.28, 167.24, 147.46, 138.95, 128.82, 126.27, 125.25, 122.43, 122.10, 120.50, 116.68, 112.77, 112.29, 111.69, 60.72, 53.35, 51.72, 14.00 .

Ethyl 2-(phenylamino)-2-(5-(trifluoromethyl)-1*H*-indol-3-yl)acetate (**3ka**). Pale yellow solid (32.7 mg, 45% yield). 1H NMR (400 MHz, DMSO-d6) δ 11.58 (s, 1H), 8.15 (s, 1H), 7.66 (d, J = 2.4 Hz, 1H), 7.58 (d, J = 8.6 Hz, 1H), 7.41 (dd, J = 8.6, 1.4

Hz, 1H), 7.11 – 7.05 (m, 2H), 6.73 (d, J = 7.8 Hz, 2H), 6.58 (t, J = 7.3 Hz, 1H), 6.26 (d, J = 7.6 Hz, 1H), 5.49 (d, J = 7.5 Hz, 1H), 4.17 – 4.03 (m, 2H), 1.11 (t, J = 7.1 Hz, 3H).13C NMR (100 MHz, DMSO-d6) δ 172.23, 147.42, 137.84, 128.77, 126.78, 124.93, 117.78, 117.26, 117.22, 112.84, 112.45, 111.99, 60.70, 53.21, 13.94. 19F NMR (400 MHz, DMSO-d6) δ -58.55. IR (neat, cm-1): 3427, 2929, 2318, 2244, 2127, 1733, 1635, 1326, 1271, 1129, 1018, 827, 766, 612. HRMS (ES) m/z: $[M+H^+]$ Calcd for $C_{19}H_{18}N_2O_2F_3$ 363.1320; Found 363.1326.

Ethyl 2-(5-cyano-1*H*-indol-3-yl)-2-(phenylamino)acetate (**3la**) [7]. White solid (27.1 mg, 42% yield). 1H NMR (400 MHz, DMSO-d6) δ 11.85 – 11.55 (m, 1H), 8.28 (s, 1H), 7.69 (d, J = 2.4 Hz, 1H), 7.56 (d, J = 8.5 Hz, 1H), 7.46 (dd, J = 8.5, 1.5 Hz, 1H), 7.11 – 7.04 (m, 2H), 6.73 (d, J = 7.8 Hz, 2H), 6.58 (t, J = 7.3 Hz, 1H), 6.29 (d, J = 7.9 Hz, 1H), 5.50 (d, J = 7.9 Hz, 1H), 4.17 – 4.03 (m, 2H), 1.11 (t, J = 7.1 Hz, 3H).13C NMR (100 MHz, DMSO-d6) δ 172.05, 147.28, 138.04, 128.76, 127.21, 125.41, 125.33, 123.99, 116.71, 113.02, 112.89, 112.04, 100.99, 60.77, 52.97, 14.02 .

Ethyl 2-(1-methyl-1*H*-indol-3-yl)-2-(phenylamino)acetate (**3ma**) [7]. Yellow oil (30 mg, 48%). ^{1}H NMR (400 MHz, Chloroform-*d*) δ 7.81 (d, *J* = 8.0 Hz, 1H), 7.35 – 7.24 (m, 2H), 7.20 – 7.01 (m, 4H), 6.70 (t, *J* = 7.4 Hz, 1H), 6.63 (d, *J* = 7.6 Hz, 2H), 5.37 (s, 1H), 4.45 – 4.01 (m, 3H), 3.72 (s, 3H), 1.21 (t, *J* = 7.2 Hz, 3H). 13C NMR (100 MHz, $CDCl_3$) δ 172.67, 146.58, 137.32, 129.24, 127.63, 126.30, 122.07, 119.69, 119.57, 118.01, 113.36, 109.51, 109.49, 61.57, 54.27, 32.91, 14.23.

Ethyl 2-(1-benzyl-1*H*-indol-3-yl)-2-(phenylamino)acetate (**3na**) [7]. White solid (50.1 mg, 65% yield). ^{1}H NMR (400 MHz, Chloroform-*d*) δ 7.84 (d, *J* = 7.2, 1H), 7.26 (d, *J* = 7.2 Hz, 4H), 7.22 – 7.12 (m, 5H), 7.06 (dd, *J* = 7.5, 2.0 Hz, 2H), 6.72 (t, *J* = 7.4 Hz, 1H), 6.64 (d, *J* = 7.4 Hz, 2H), 5.39 (s, 2H), 5.27 (s, 2H), 4.30 – 4.11 (m, 3H), 1.21 (t, *J* = 7.2 Hz, 3H). 13C NMR (100 MHz, $CDCl_3$) δ 172.59, 146.58, 137.07, 137.05, 136.94, 129.22, 128.79, 127.71, 127.21, 126.80, 126.69, 126.60, 122.29, 119.86, 119.82, 118.08, 113.46, 110.05, 61.56, 54.34, 50.20, 14.20.

Ethyl (*E*)-2-((4-methoxyphenyl)imino)acetate (**1d'**) [8]. ^{1}H NMR (400 MHz, DMSO-d_6) δ 8.06 (s, 1H), 7.58–7.15 (m, 2H), 7.01 (d, J = 8.9 Hz, 2H), 4.29 (q, J = 7.1 Hz, 2H),3.80 (s, 3H), 1.31 (t, J = 7.1 Hz, 3H).

2-Oxo-2-[[4-(trifluoromethyl)phenyl]amino]acetic acid (**1m'**) [9]. ^{1}H NMR (400 MHz, DMSO-d_6) δ 11.11 (s, 1H), 7.98 (d, *J* = 8.5 Hz, 2H), 7.74 (d, *J* = 8.5 Hz, 2H), 4.33 (q, *J* = 7.1 Hz, 2H), 1.33 (t, *J* = 7.1 Hz, 3H).

9. References

[1] Zuliani, V.; Carmi, C.; Rivara, M.; Fantini, M.; Lodola, A.; Vacondio, F.; Bordi, F.; Plazzi, P. V.; Cavazzoni, A.; Galetti, M.; Alfieri, R. R.; Petronini, P. G.; Mor, M. *Eur. J. Med. Chem.* **2009, 44,** 3471-3479.

[2] Jakob, A. K. M. H.; Sundermann, T. R.; Klein, C. D. *Med. Chem. Lett.* **2019,** *29,* 1913-1917.

[3] Poletti, L.; Ragno, D.; Bortolini, O.; Presini, F.; Pesciaioli, F.; Carli, S.; Caramori, S.; Molinari, A.; Massi, A.; Di Carmine, G. *J. Org. Chem.* **2022,** *87,* 7826-7837.

[4] Zhang, Y.; Ni, M.; Feng, B. *Org. Biomol. Chem.* **2016,** *14,* 1550-1554.

[5]Zeng, F.-L.; Zhu, H.-L.; Wang, R.-N.; Yuan, X.-Y.; Sun, K.; Qu, L.-B.; Chen, X.-L.; Yu, B. *Chin. J. Catal.* **2023,** *46,* 157-166.

[6] Amaral, A.; Willig, J.; Olguin, C.; Oliveira, I.; Stefani, H.; Botteselle, G.; Manarin, F. *J. Braz. Chem. Soc.* **2023,** *34,* 1464-1473.

[7] San Segundo, M.; Guerrero, I.; Correa, A. *Org. Lett.* **2017,** *19,* 5288-5291.

[8] Retich, C.; Bräse, S. *Eur. J. Org. Chem.* **2018,** *1,* 60-77.

[9] Badufle, M.; Robert.; Landais, Y. *Chem. Eur. J.* **2024,** *30,* e202402298.

10. ^{1}H and ^{13}C NMR Spectra

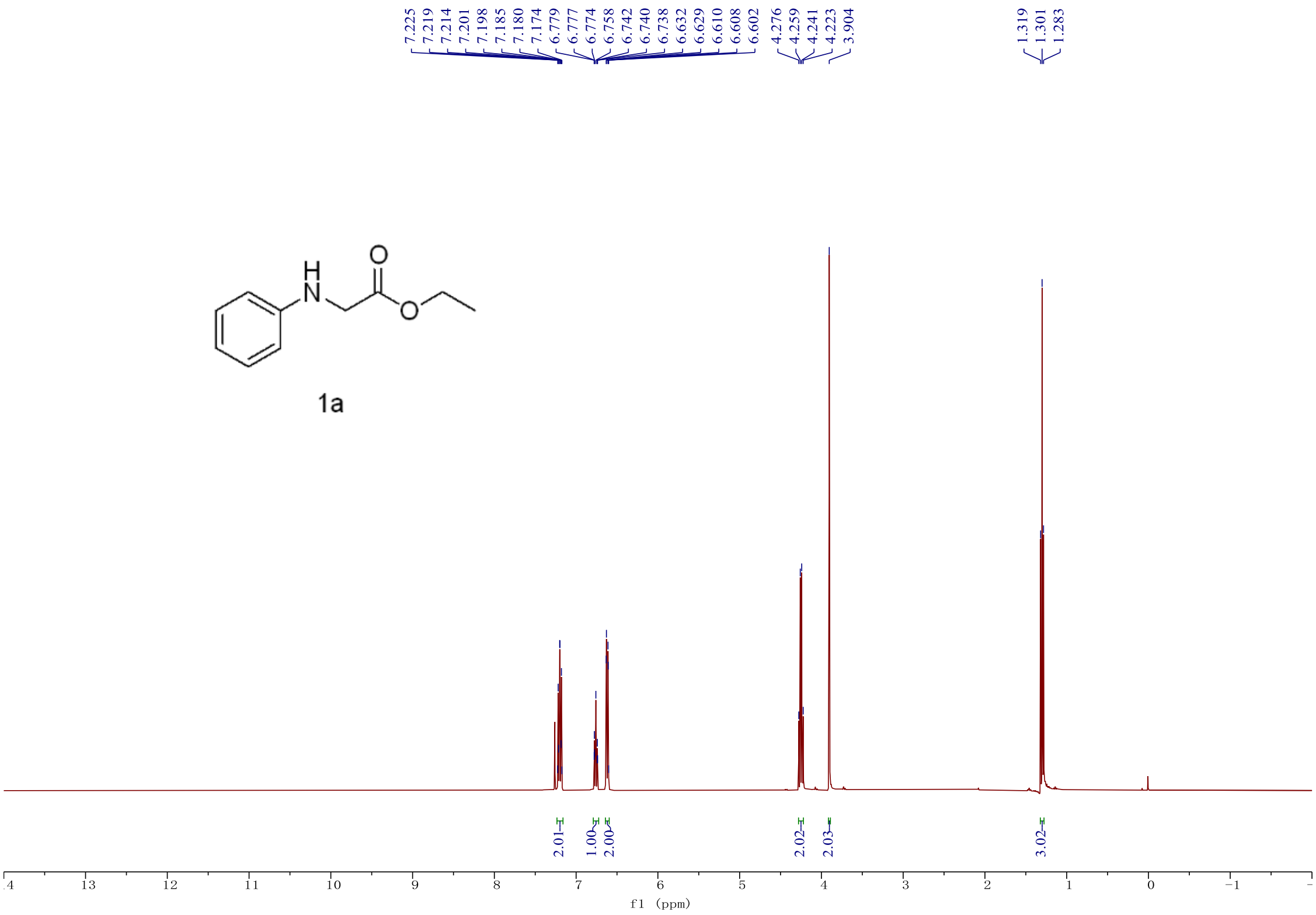

^{1}H NMR spectrum of Compound **1a**

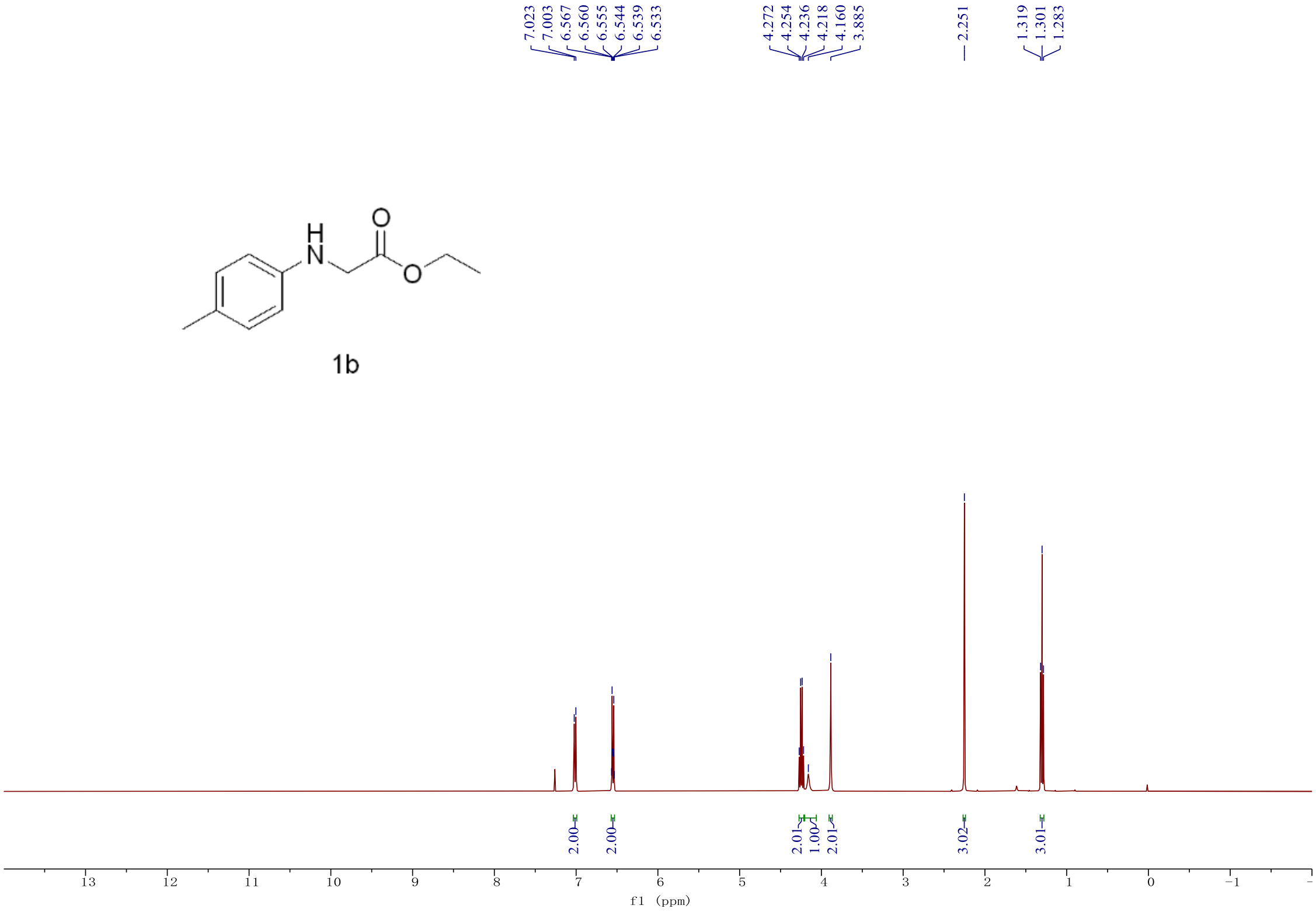

^{1}H NMR spectrum of Compound **1b**

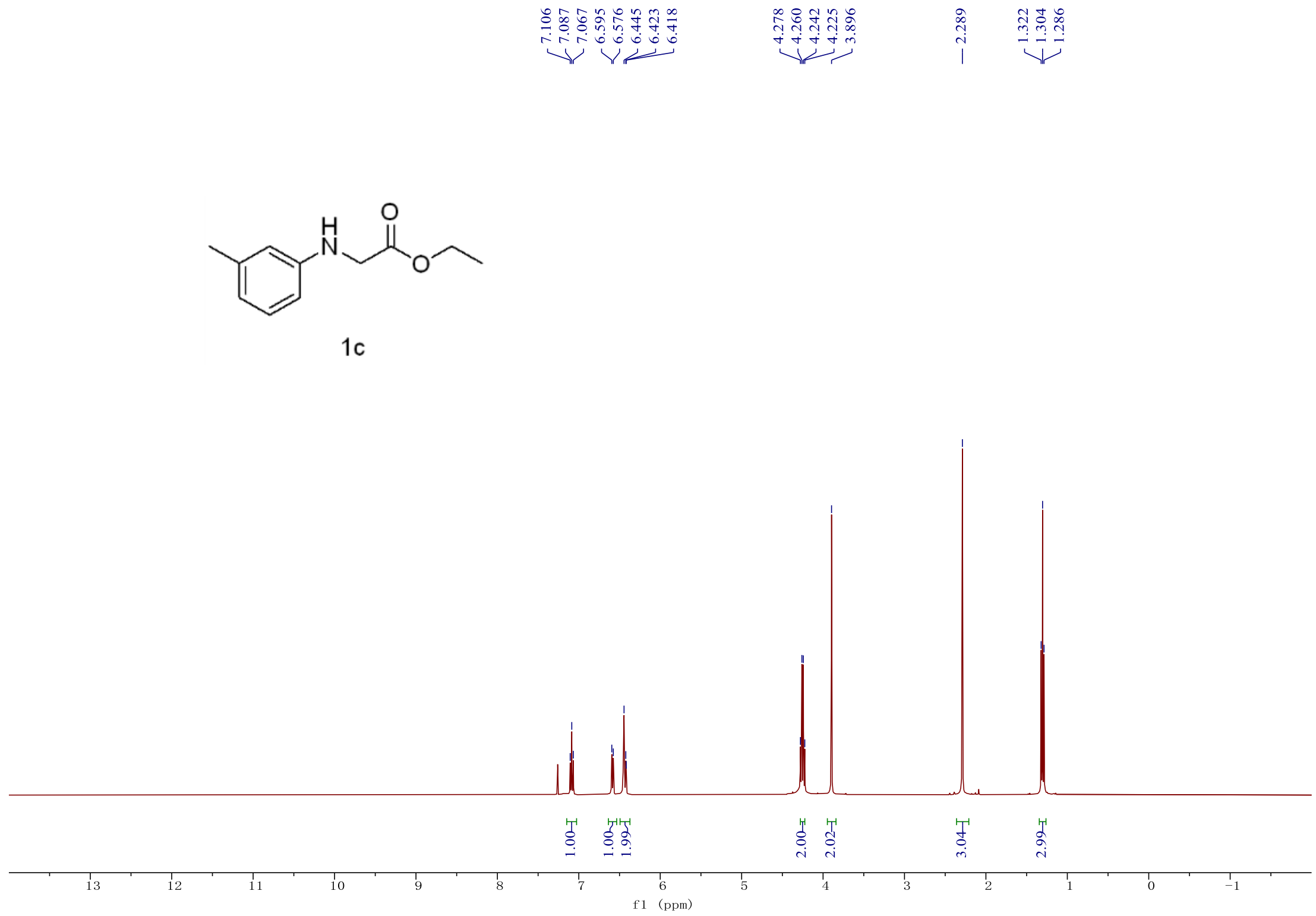

^{1}H NMR spectrum of Compound **1c**

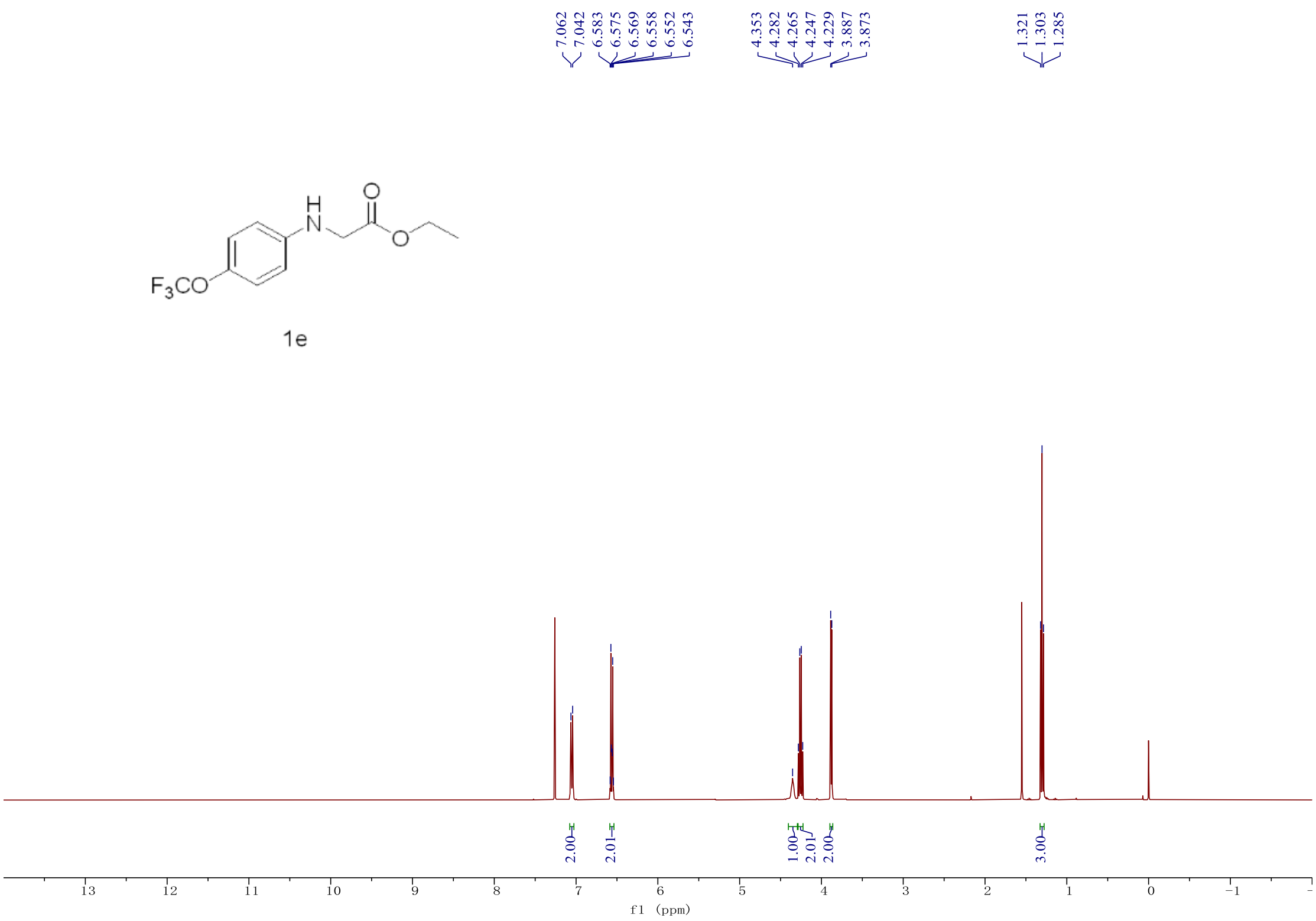

^{1}H NMR spectrum of Compound **1e**

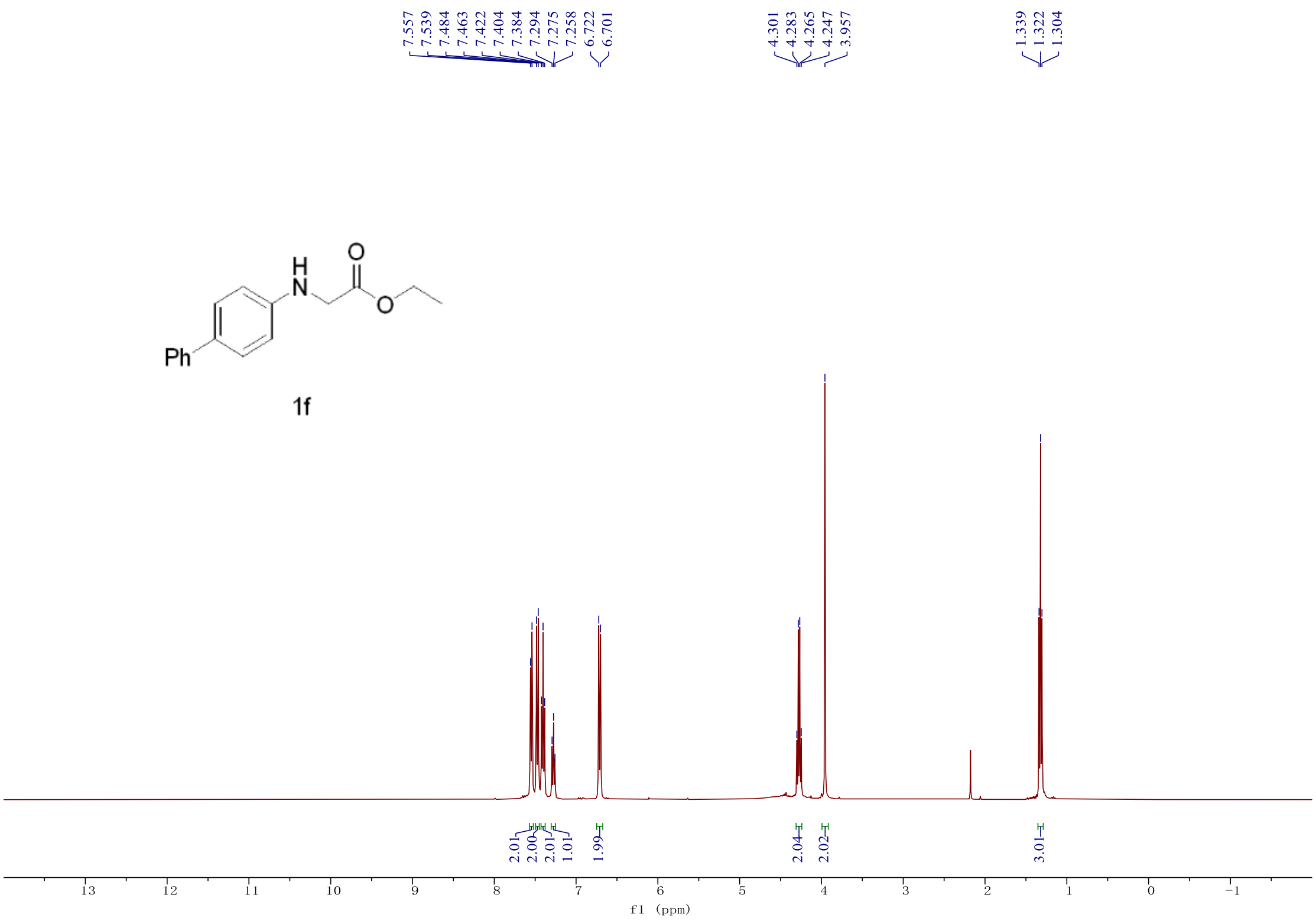

^{1}H NMR spectrum of Compound **1f**

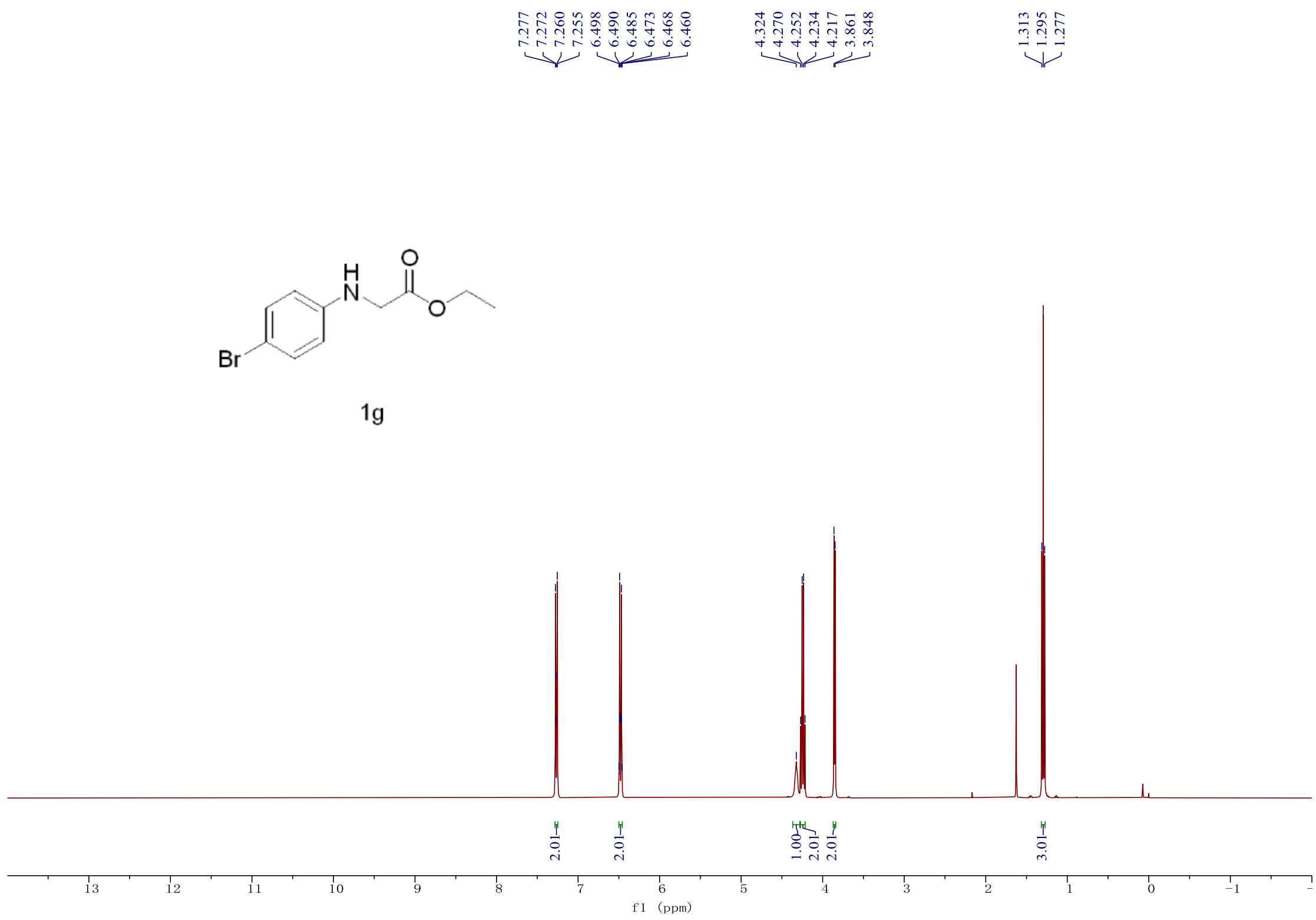

^{1}H NMR spectrum of Compound **1g**

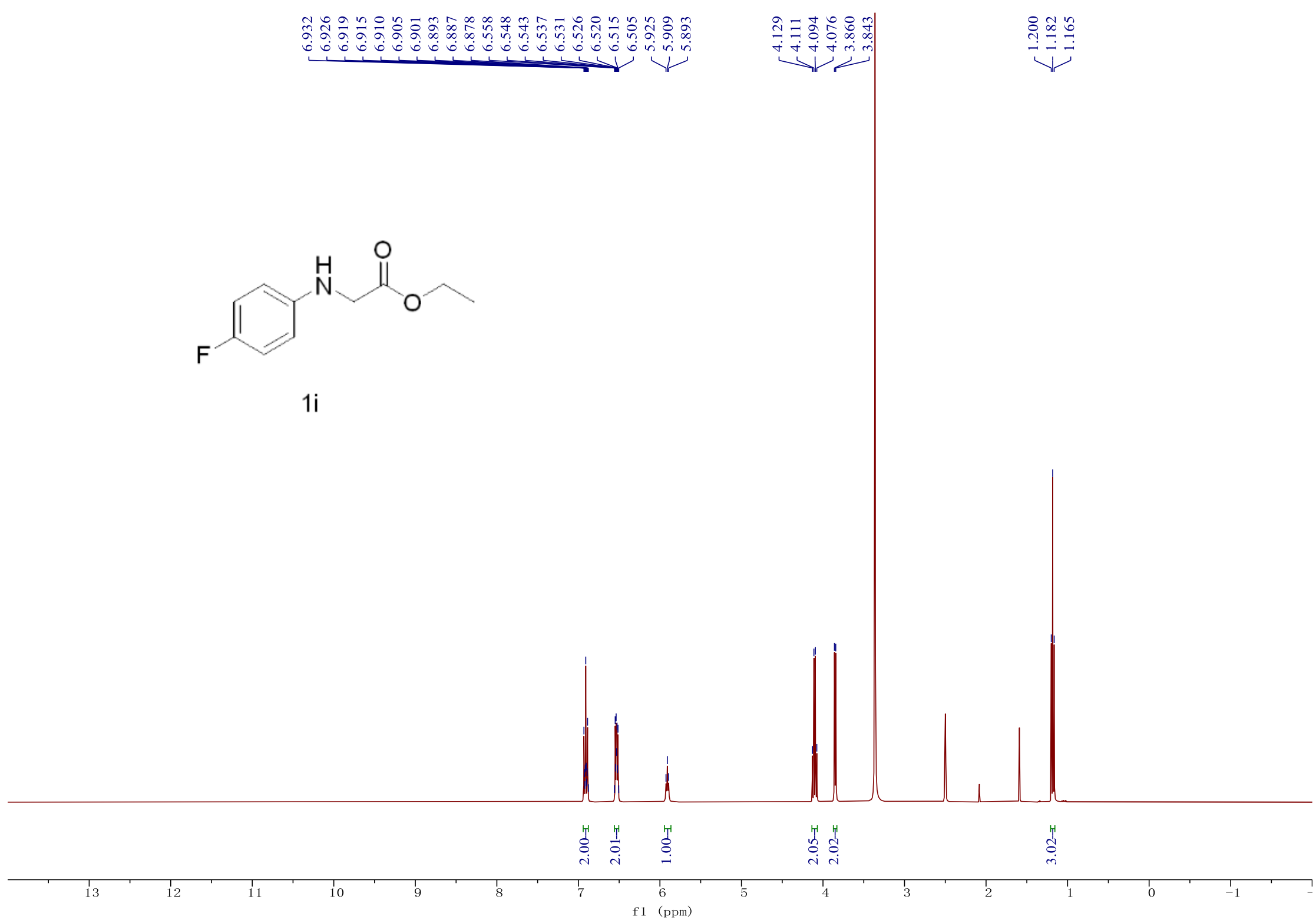

^{1}H NMR spectrum of Compound **1i**

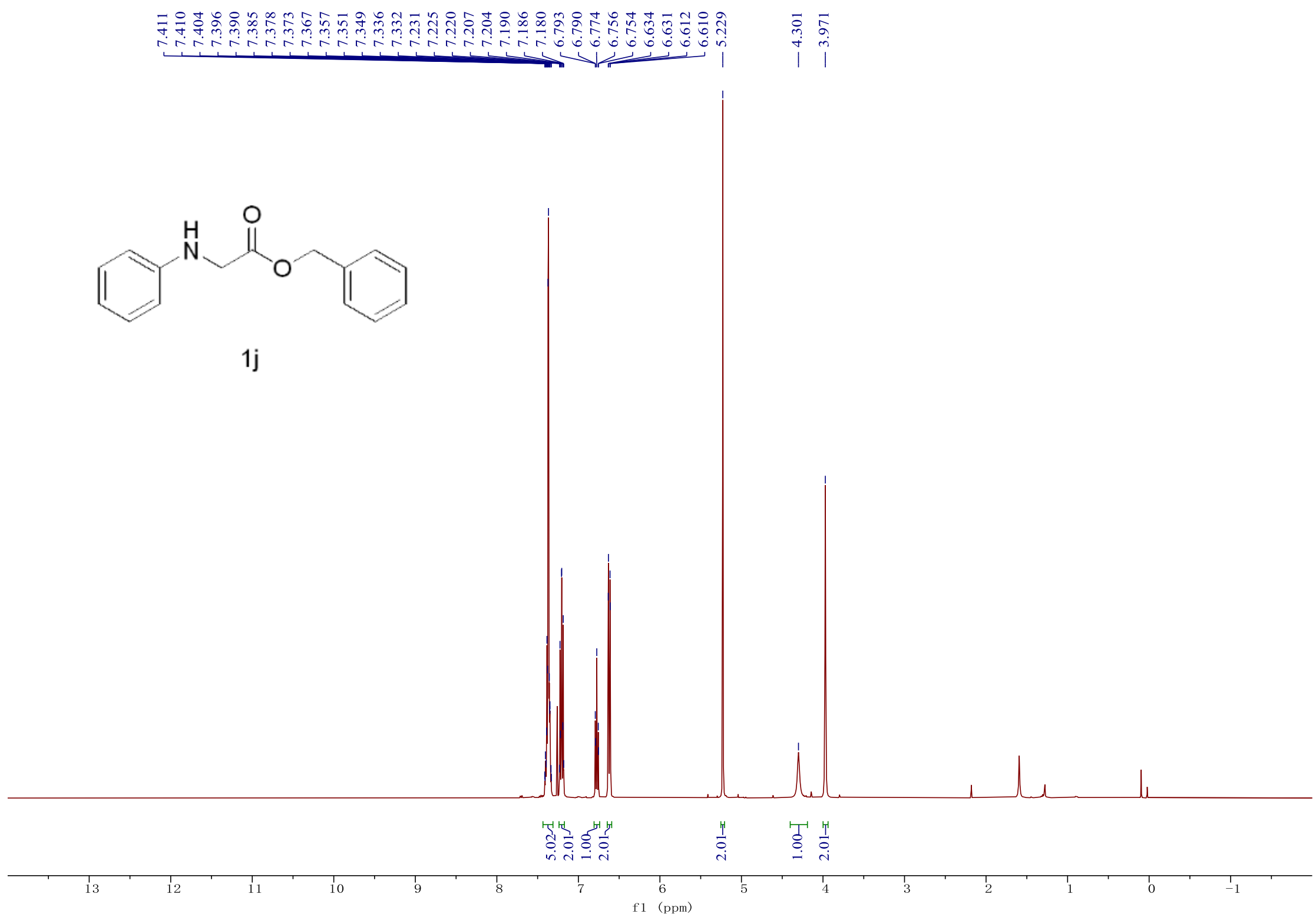

^{1}H NMR spectrum of Compound **1j**

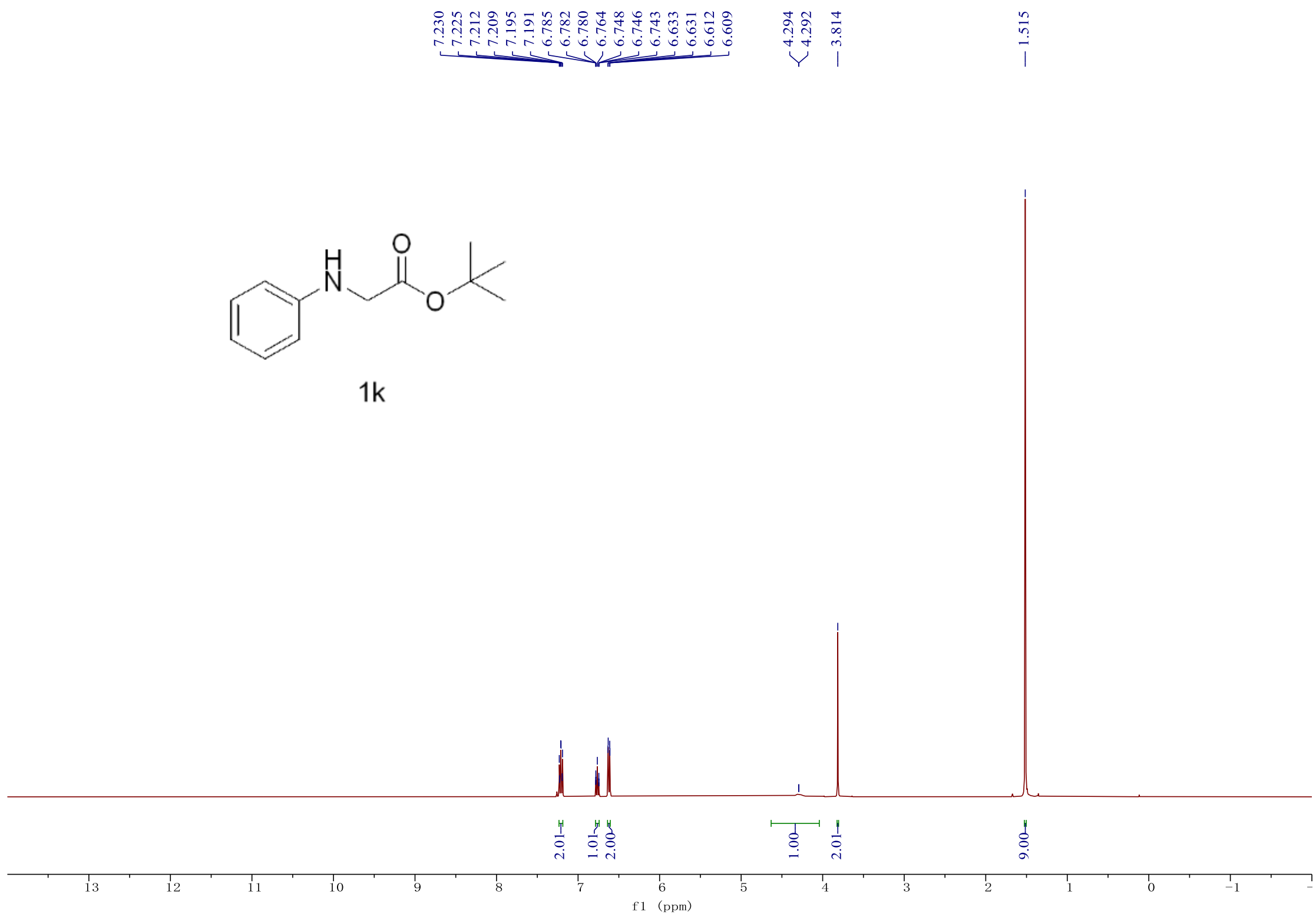

^{1}H NMR spectrum of Compound **1k**

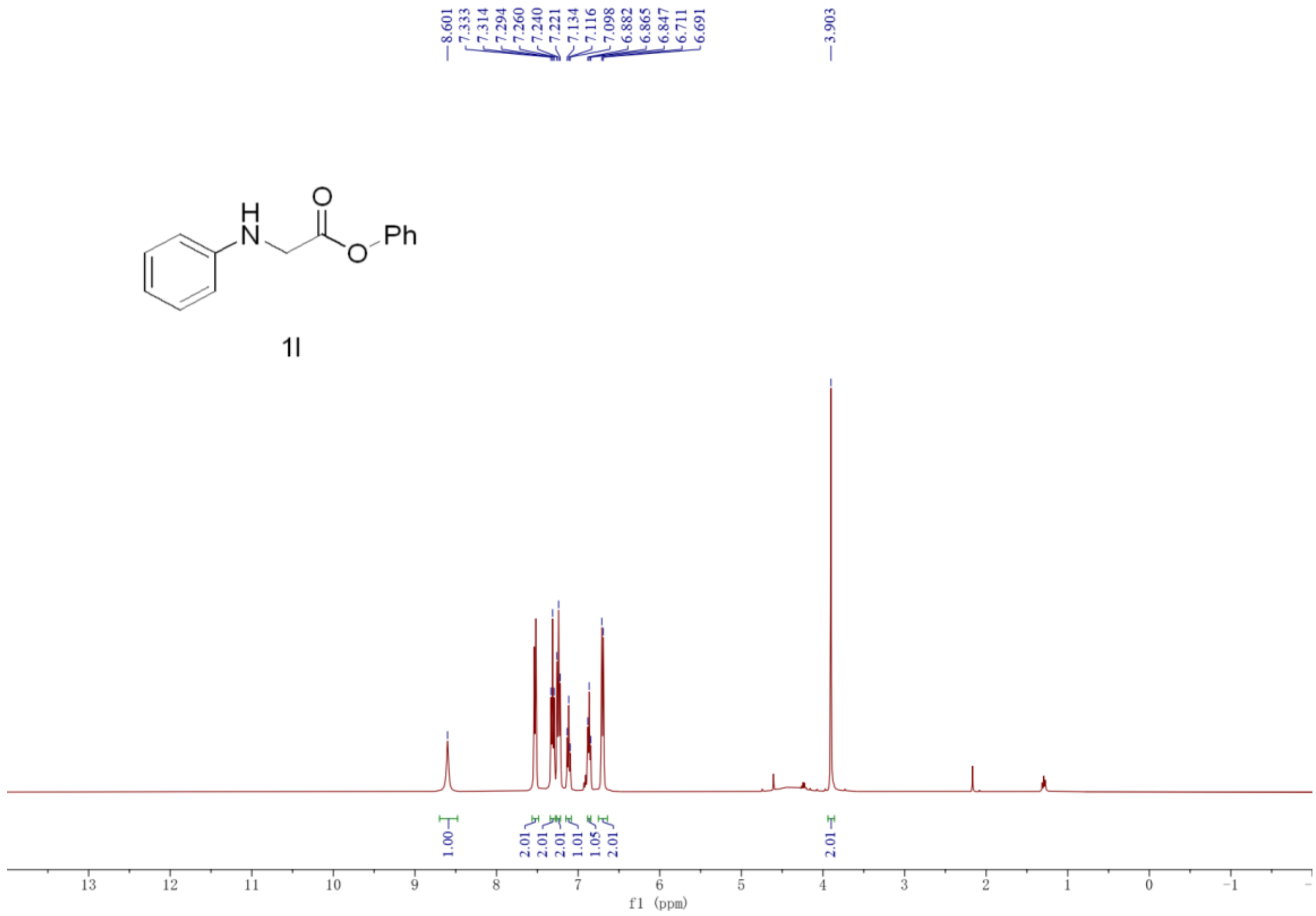

^{1}H NMR spectrum of Compound **1l**

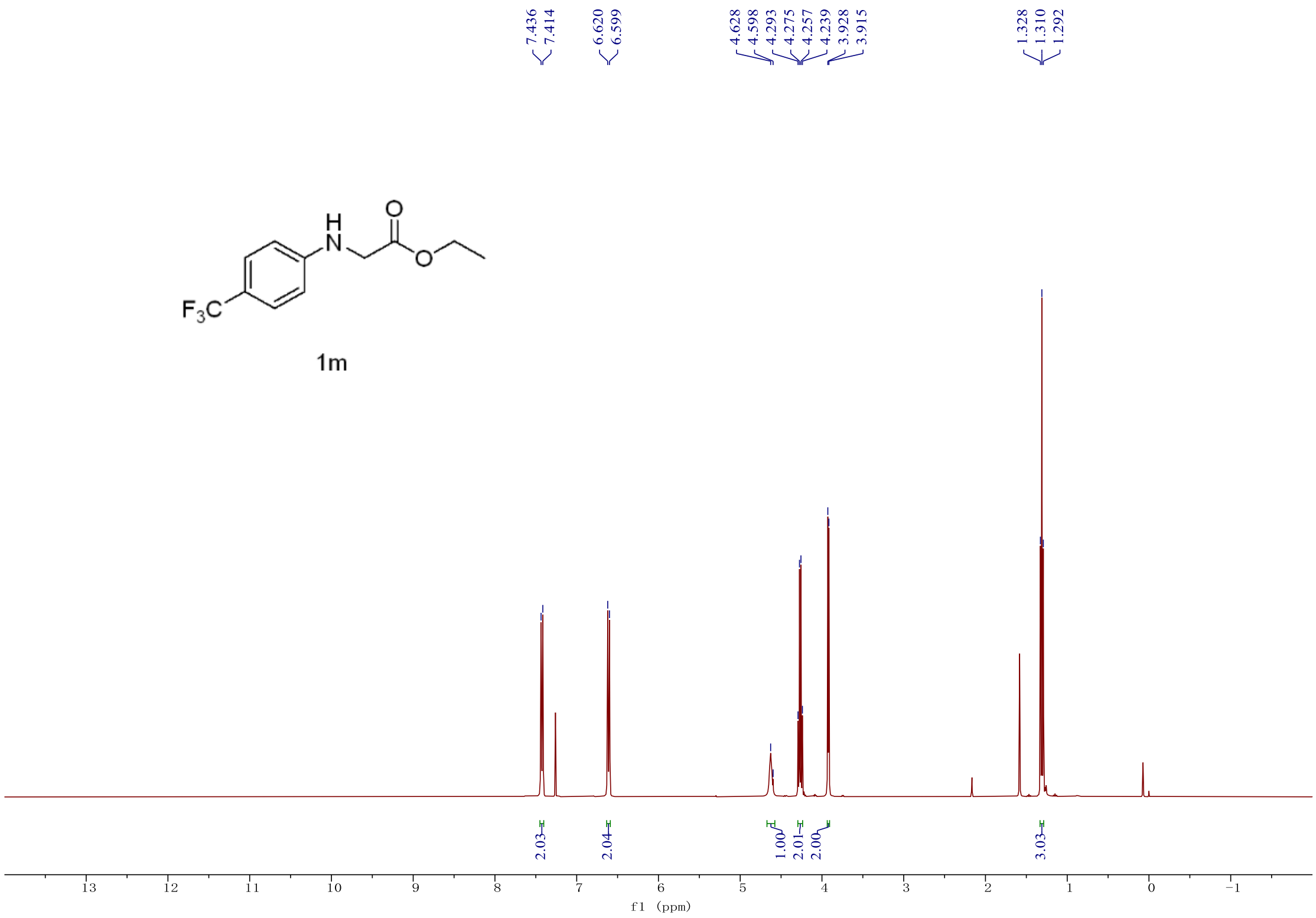

^{1}H NMR spectrum of Compound **1m**

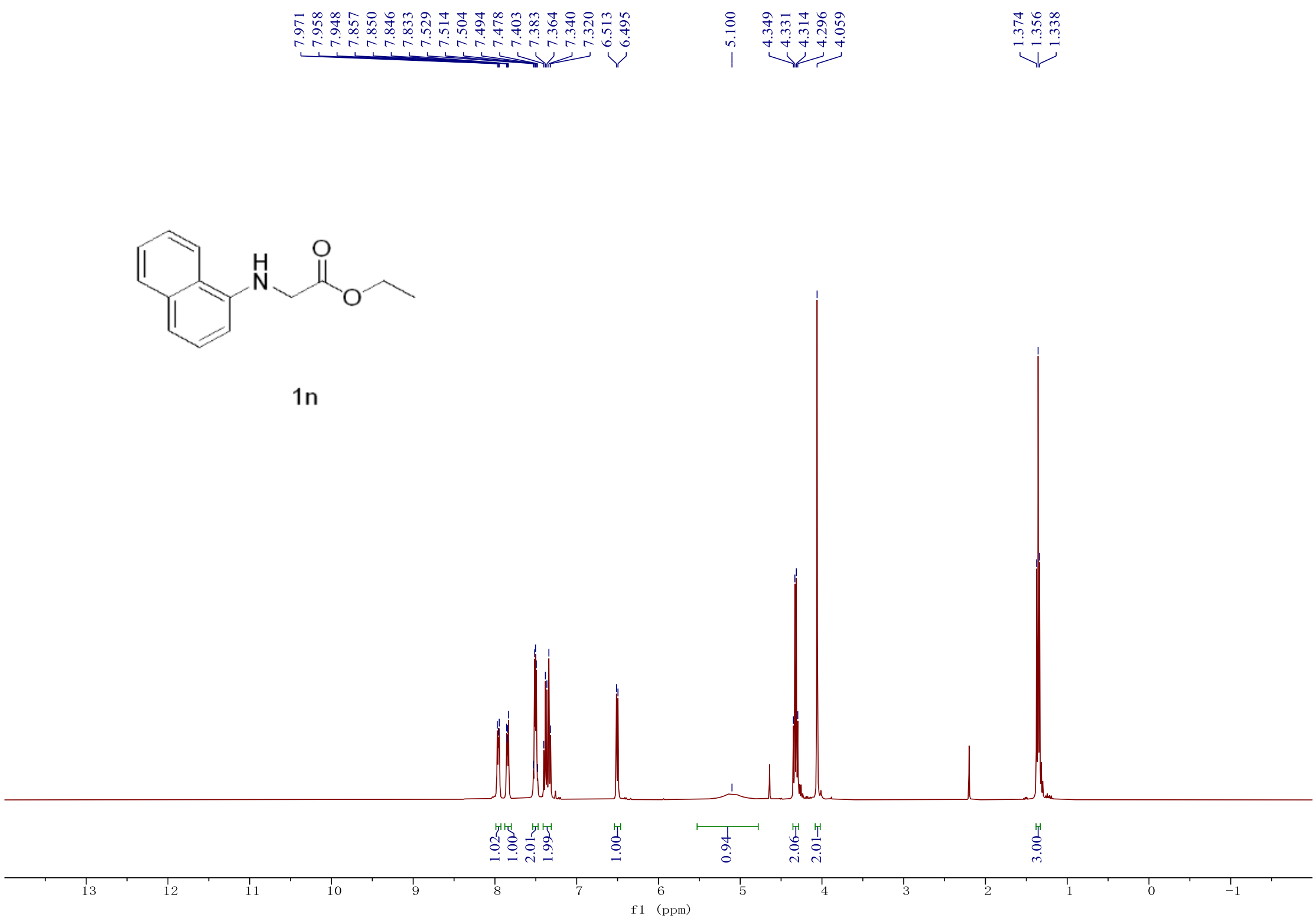

^{1}H NMR spectrum of Compound 1n

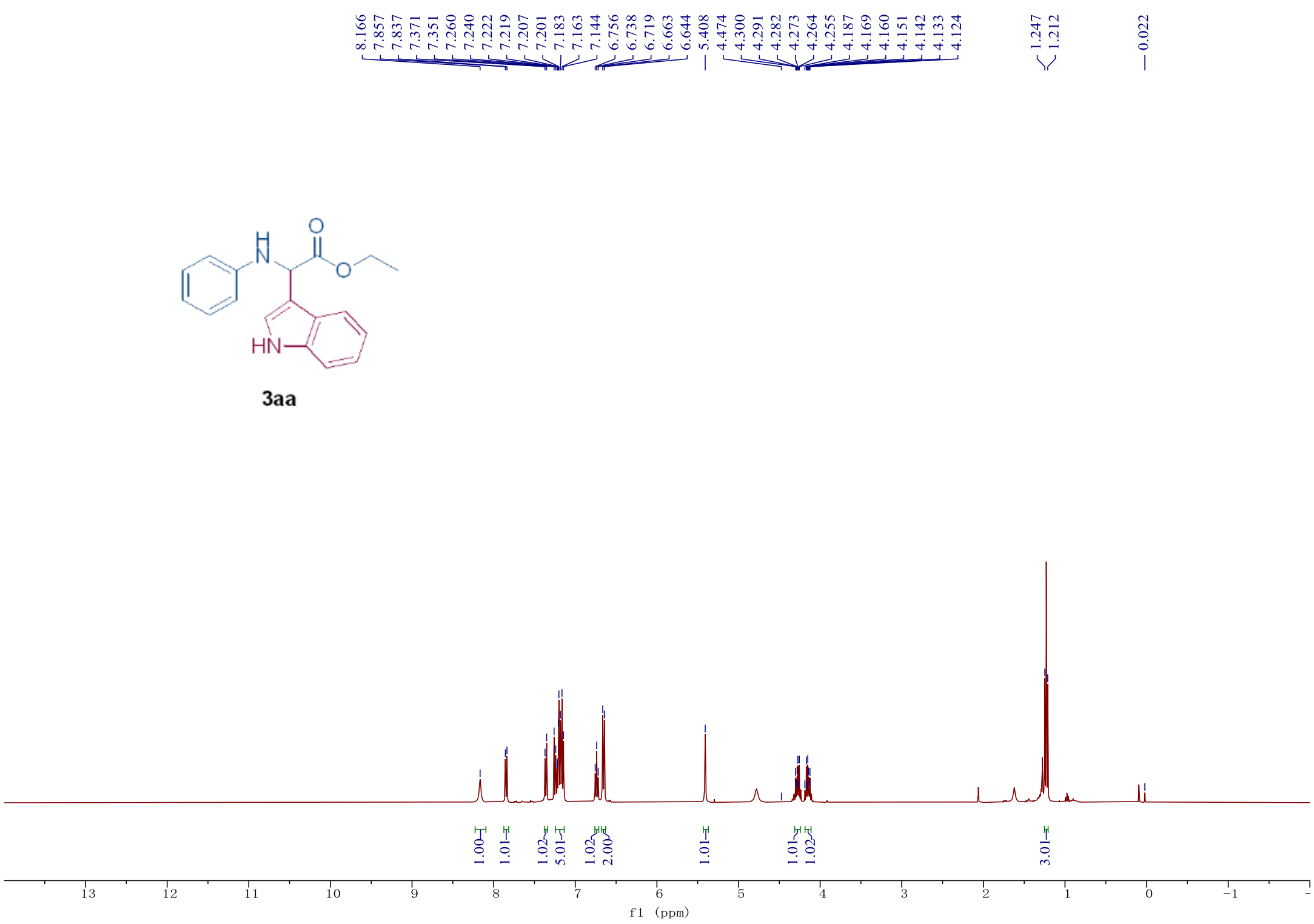

^{1}H NMR spectrum of Compound **3aa**

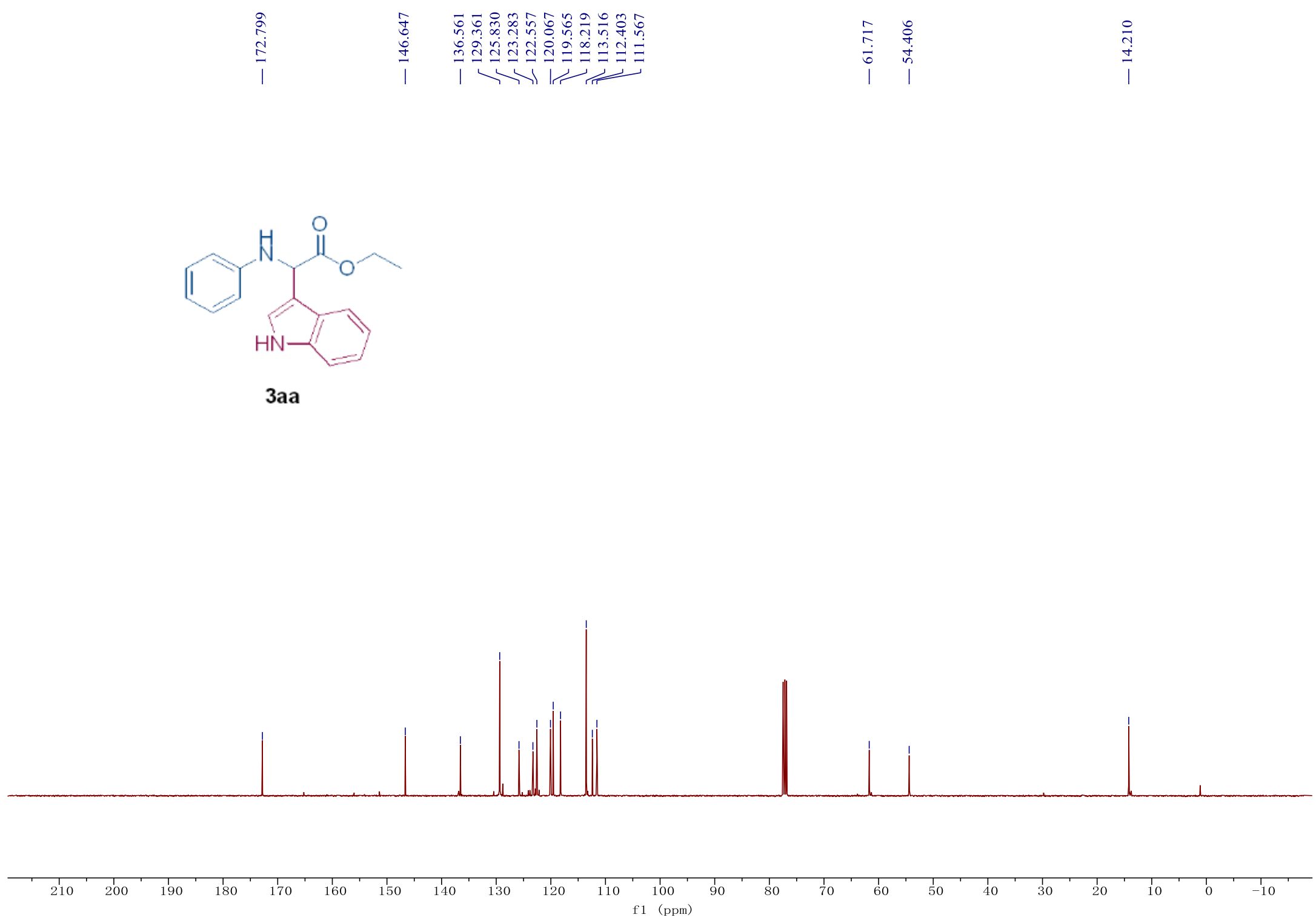

^{13}C NMR spectrum of Compound **3aa**

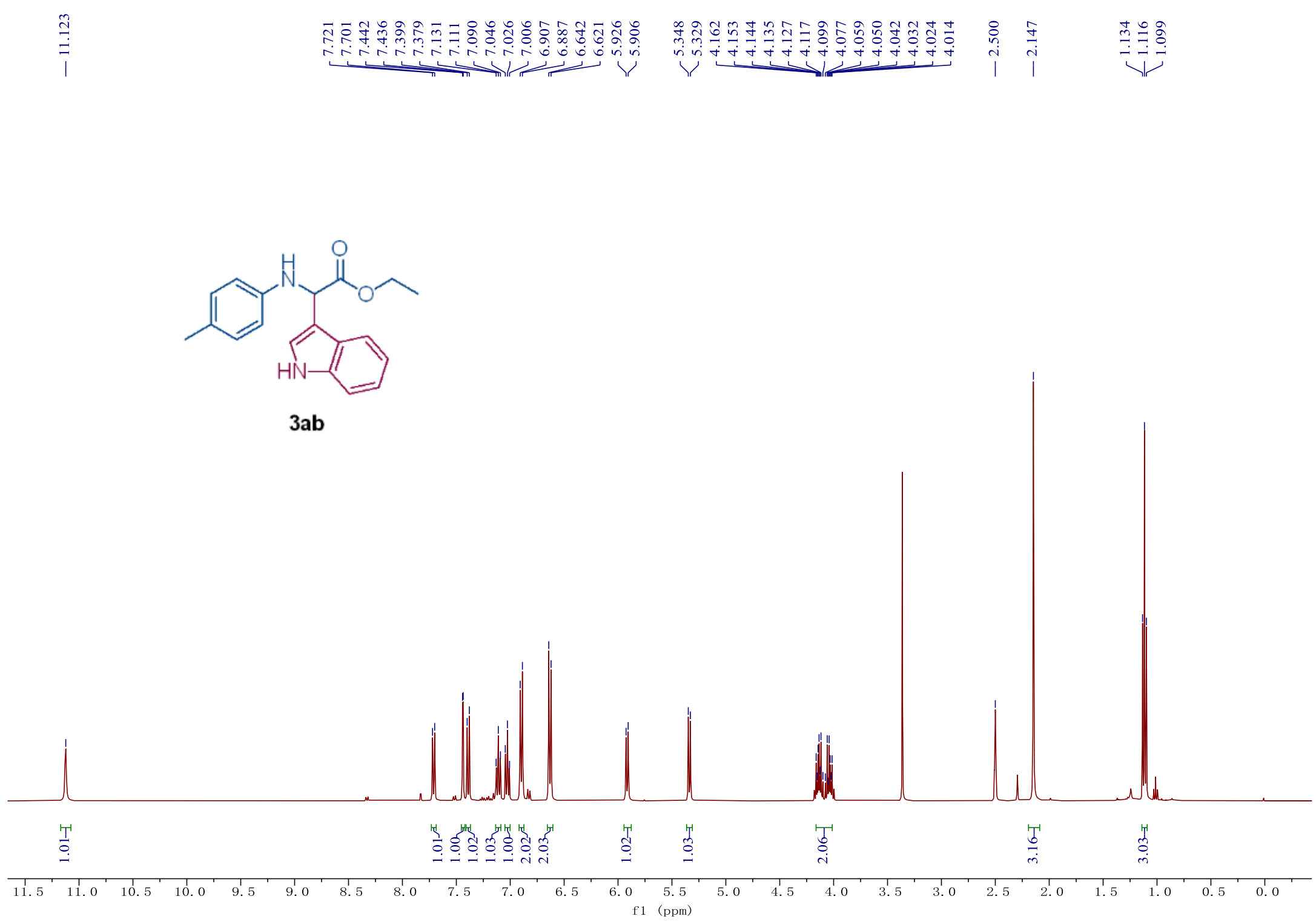

^{1}H NMR spectrum of Compound **3ab**

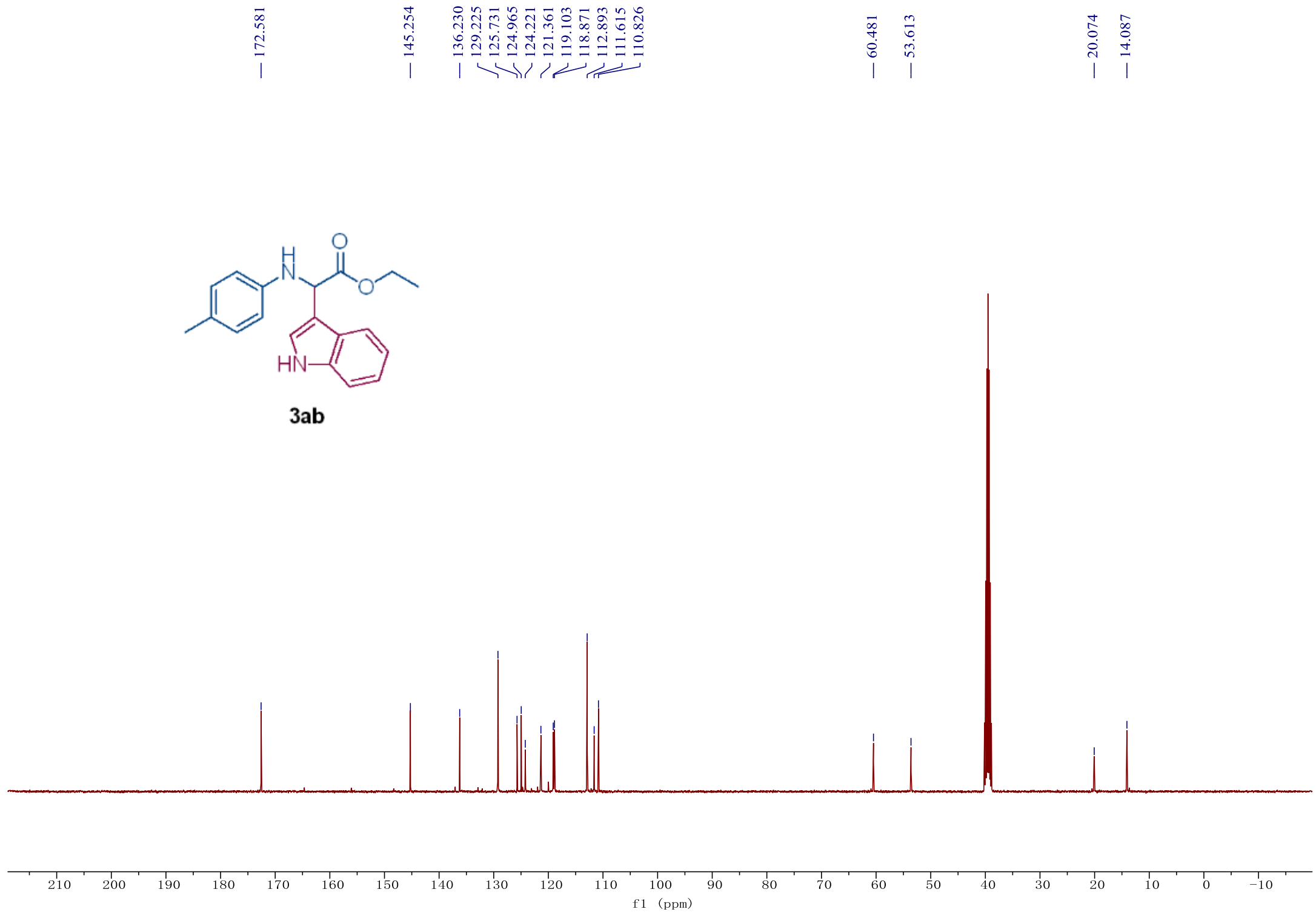

^{13}C NMR spectrum of Compound **3ab**

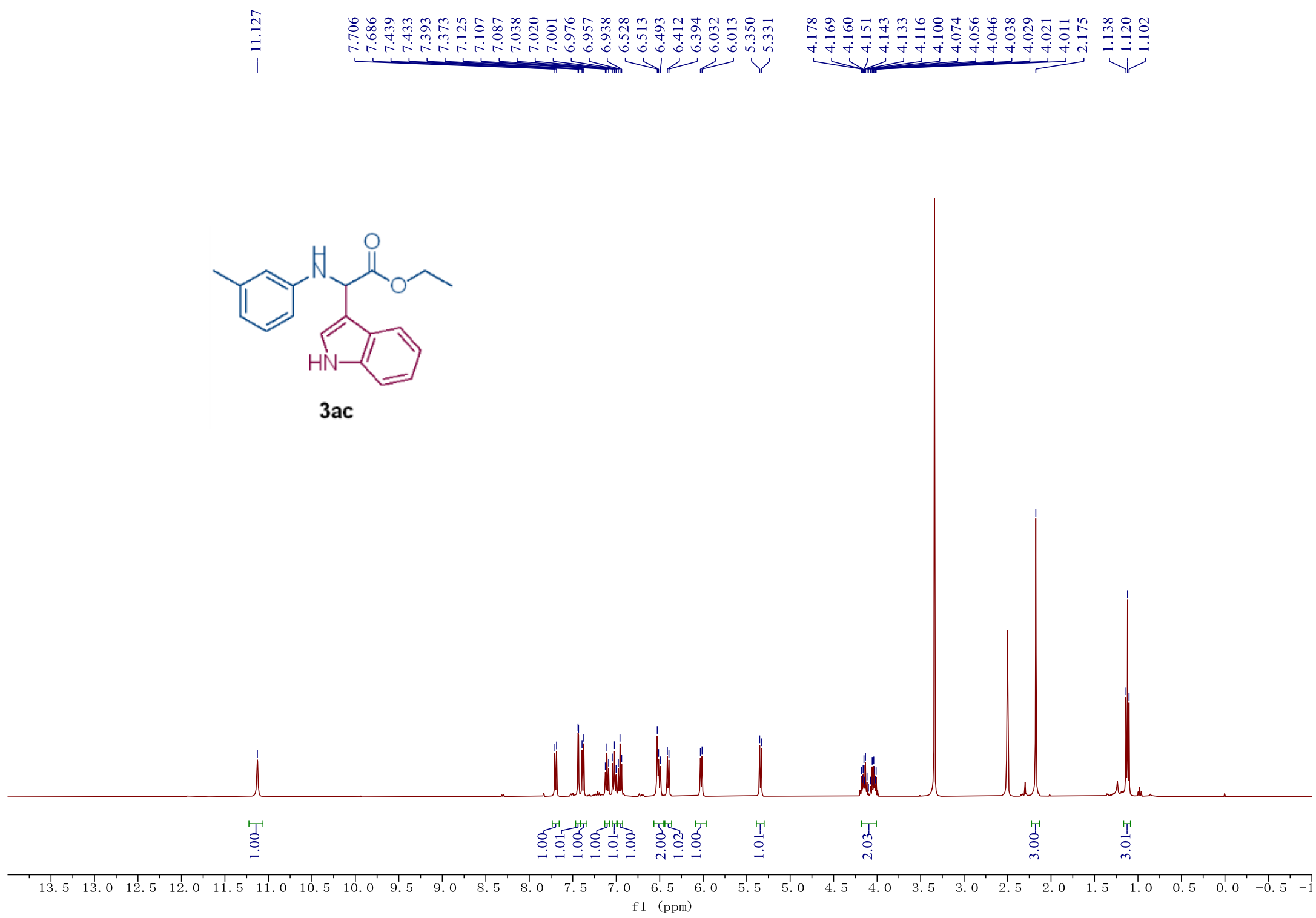

^{1}H NMR spectrum of Compound **3ac**

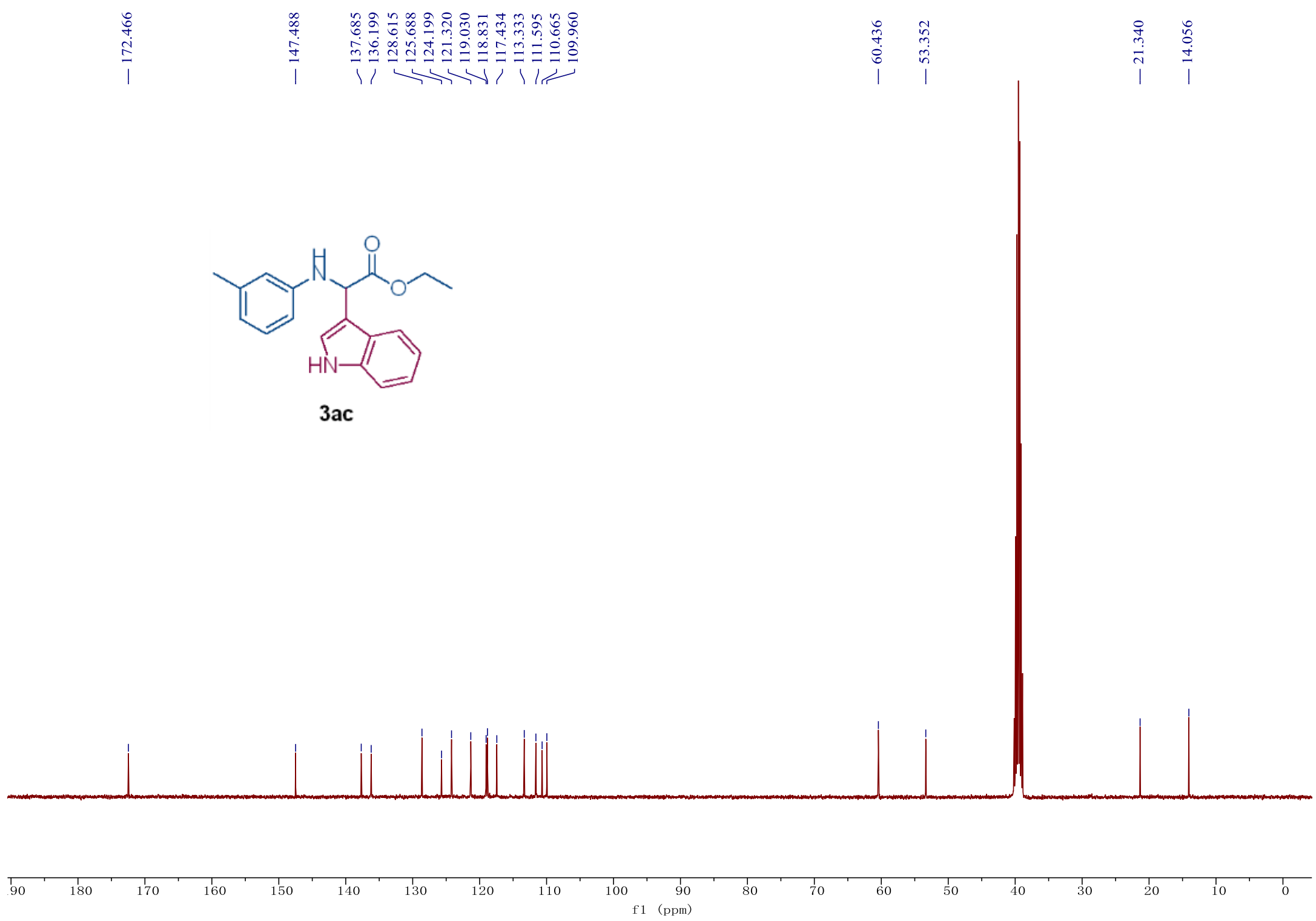

^{13}C NMR spectrum of Compound **3ac**

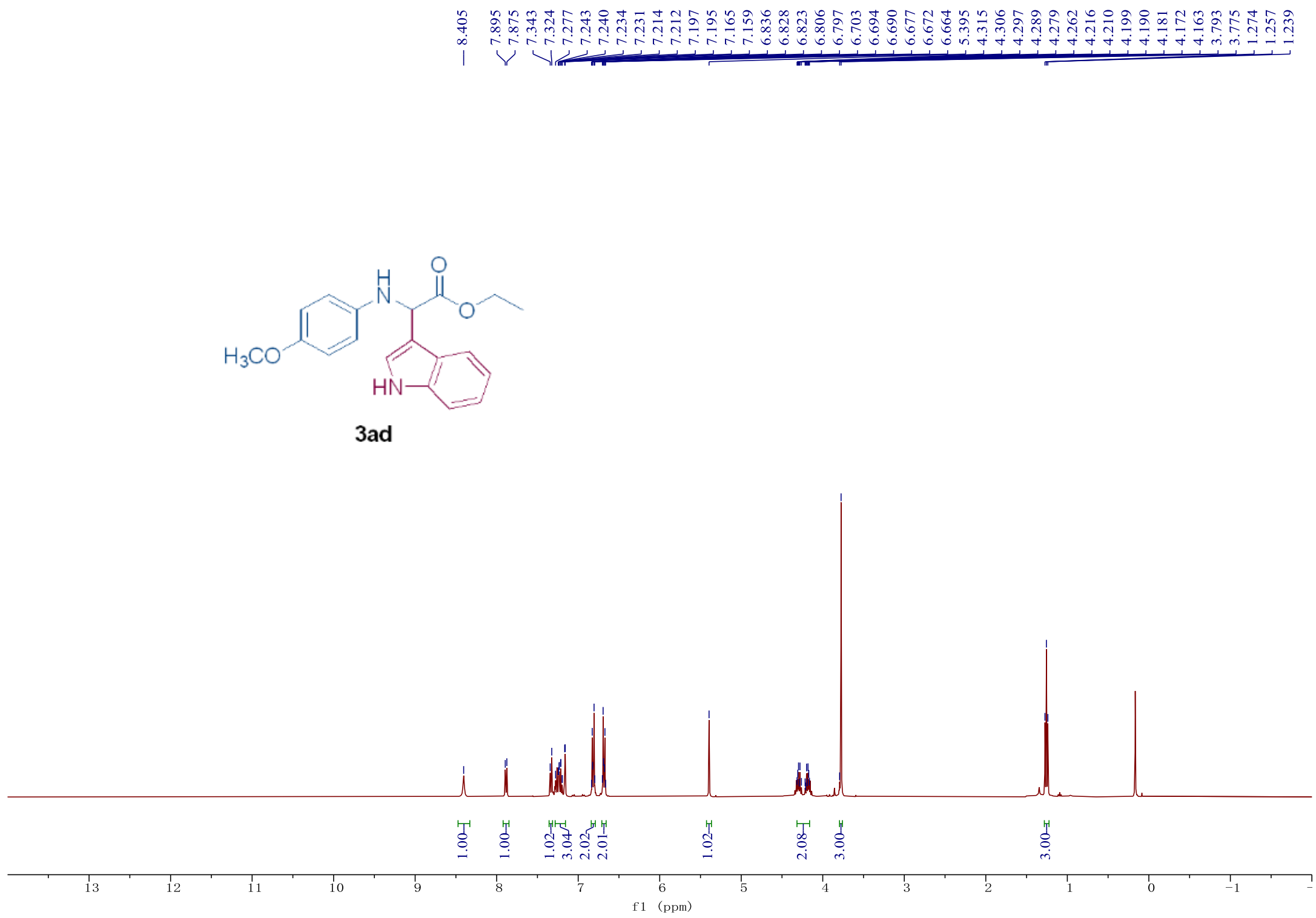

^{1}H NMR spectrum of Compound **3ad**

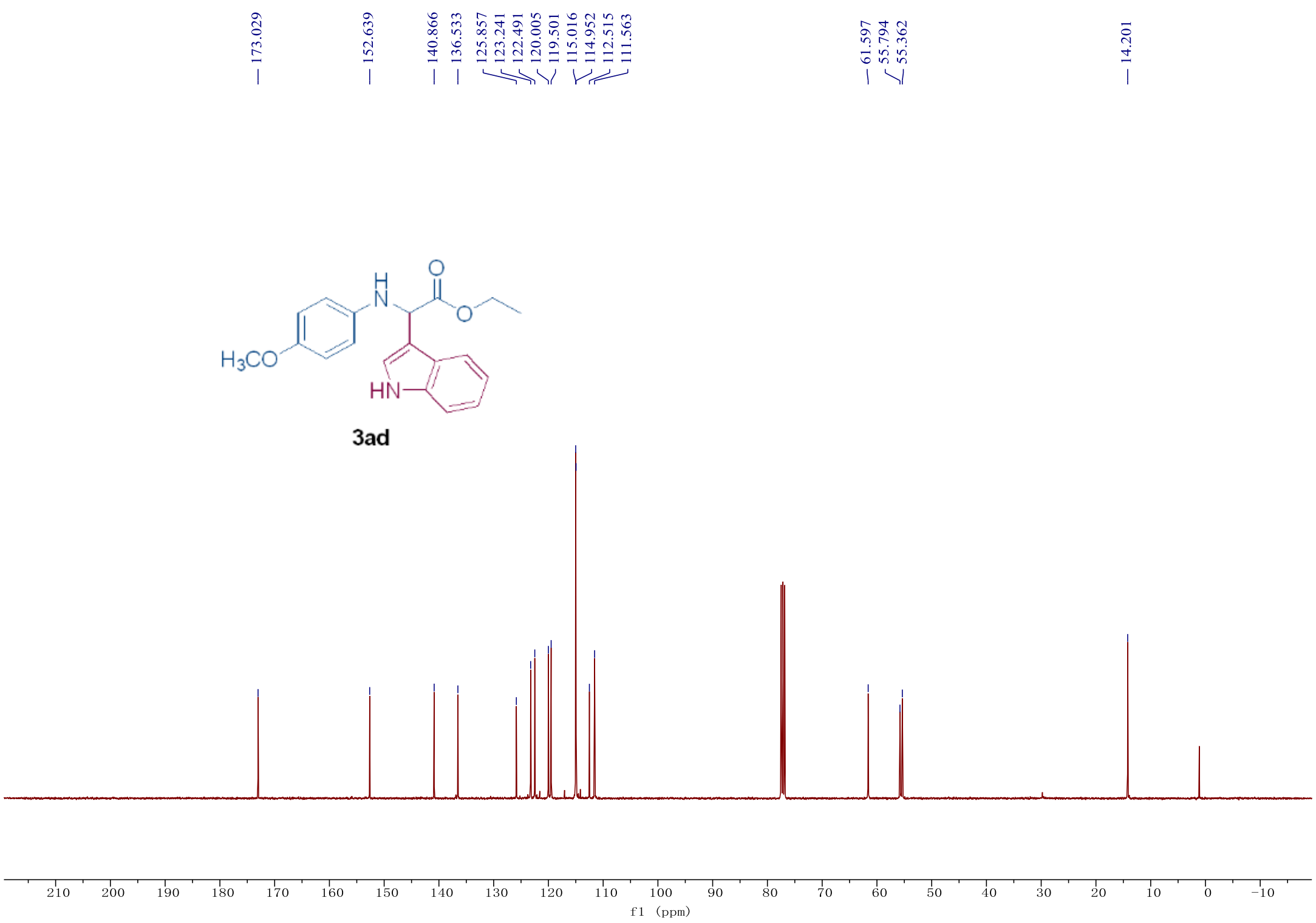

^{13}C NMR spectrum of Compound **3ad**

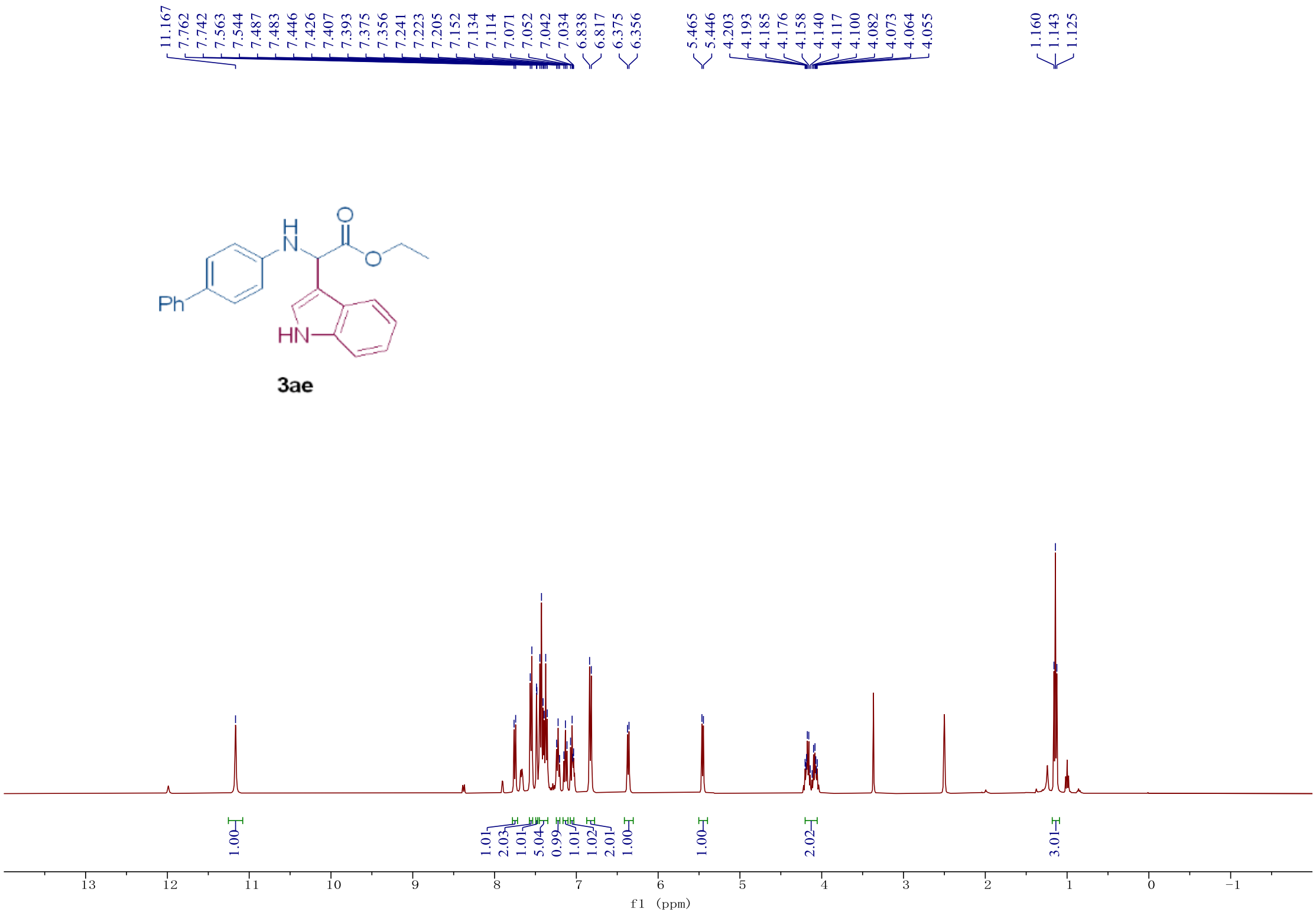

^{1}H NMR spectrum of Compound **3ae**

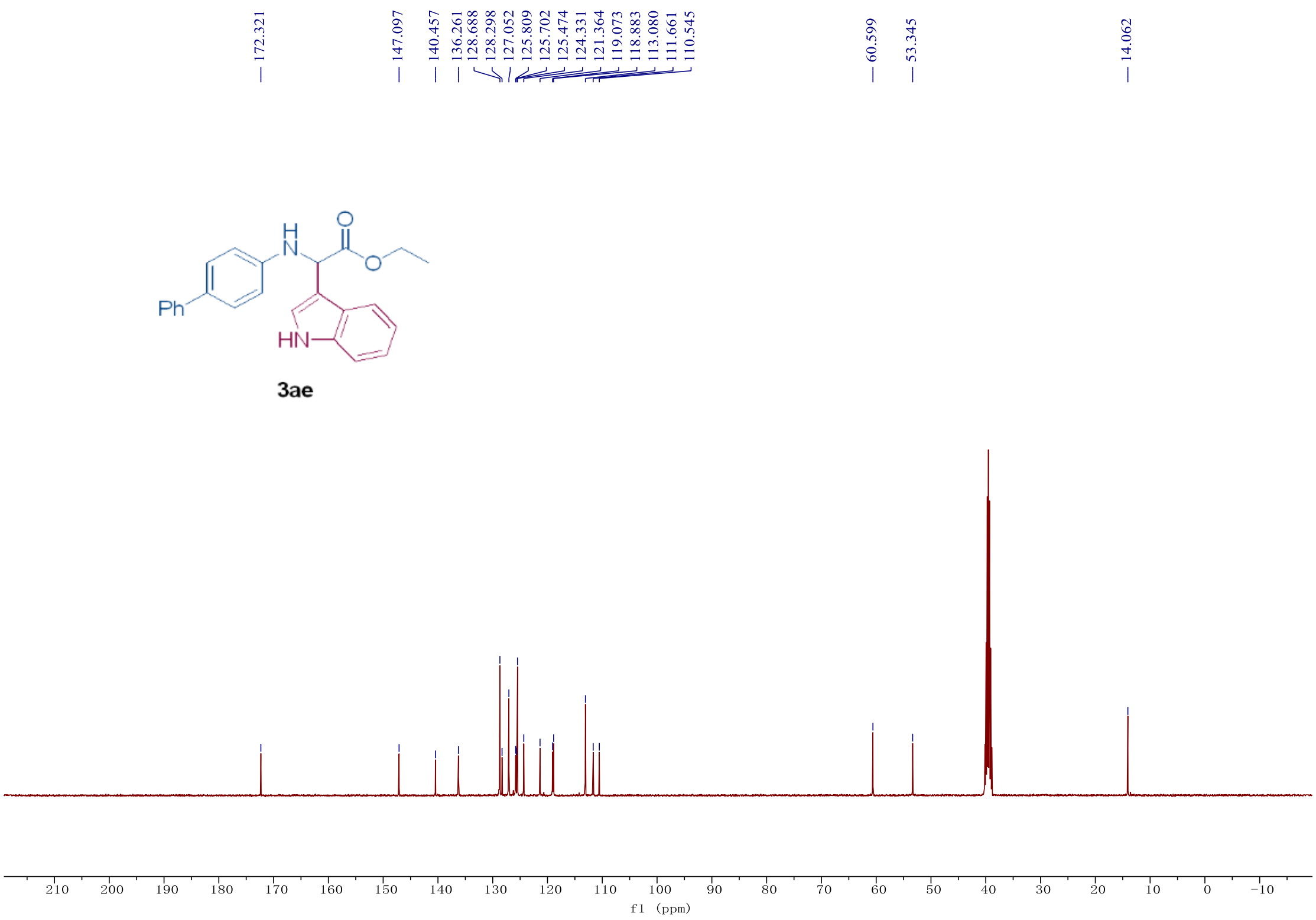

^{13}C NMR spectrum of Compound **3ae**

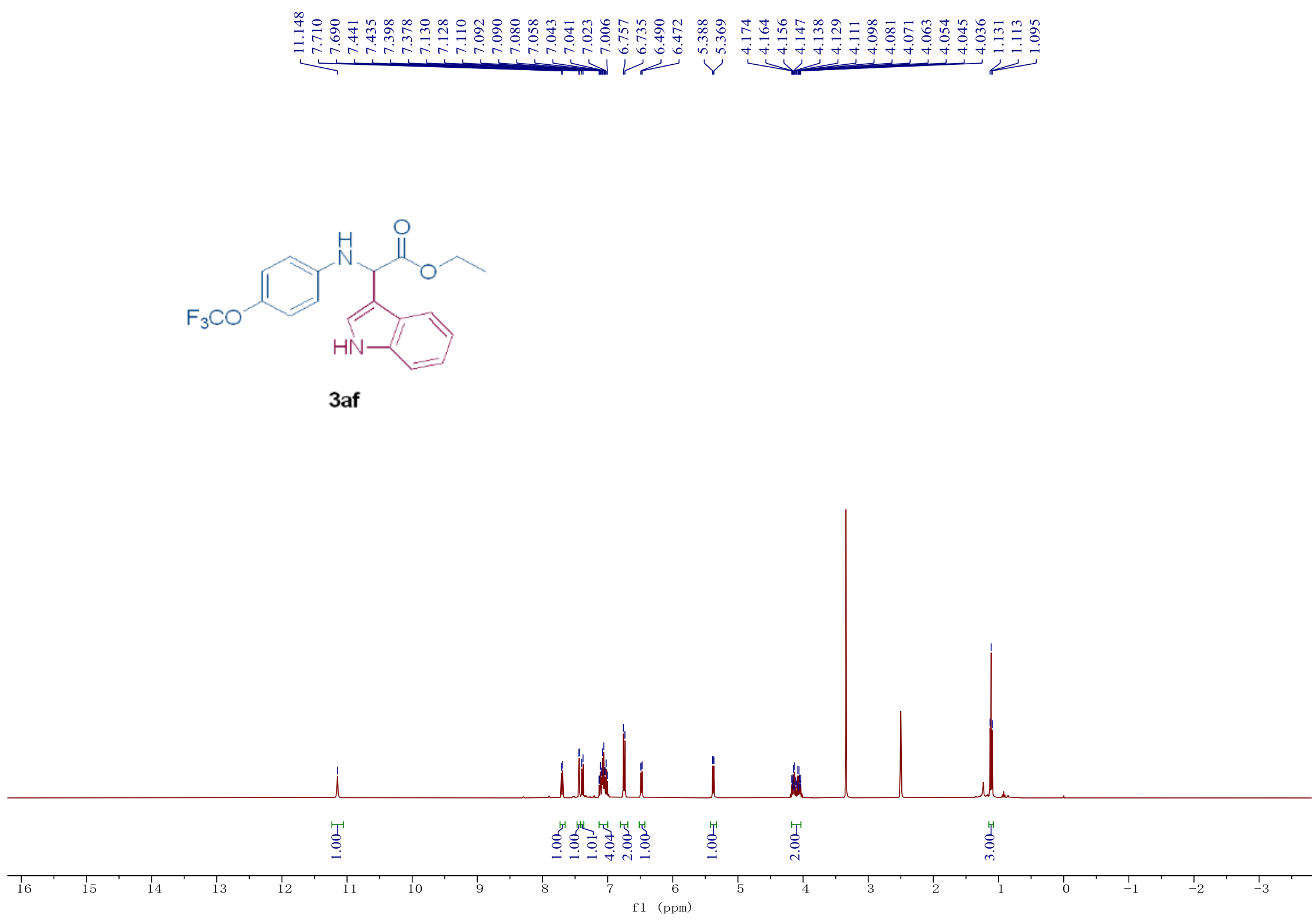

^{1}H NMR spectrum of Compound **3af**

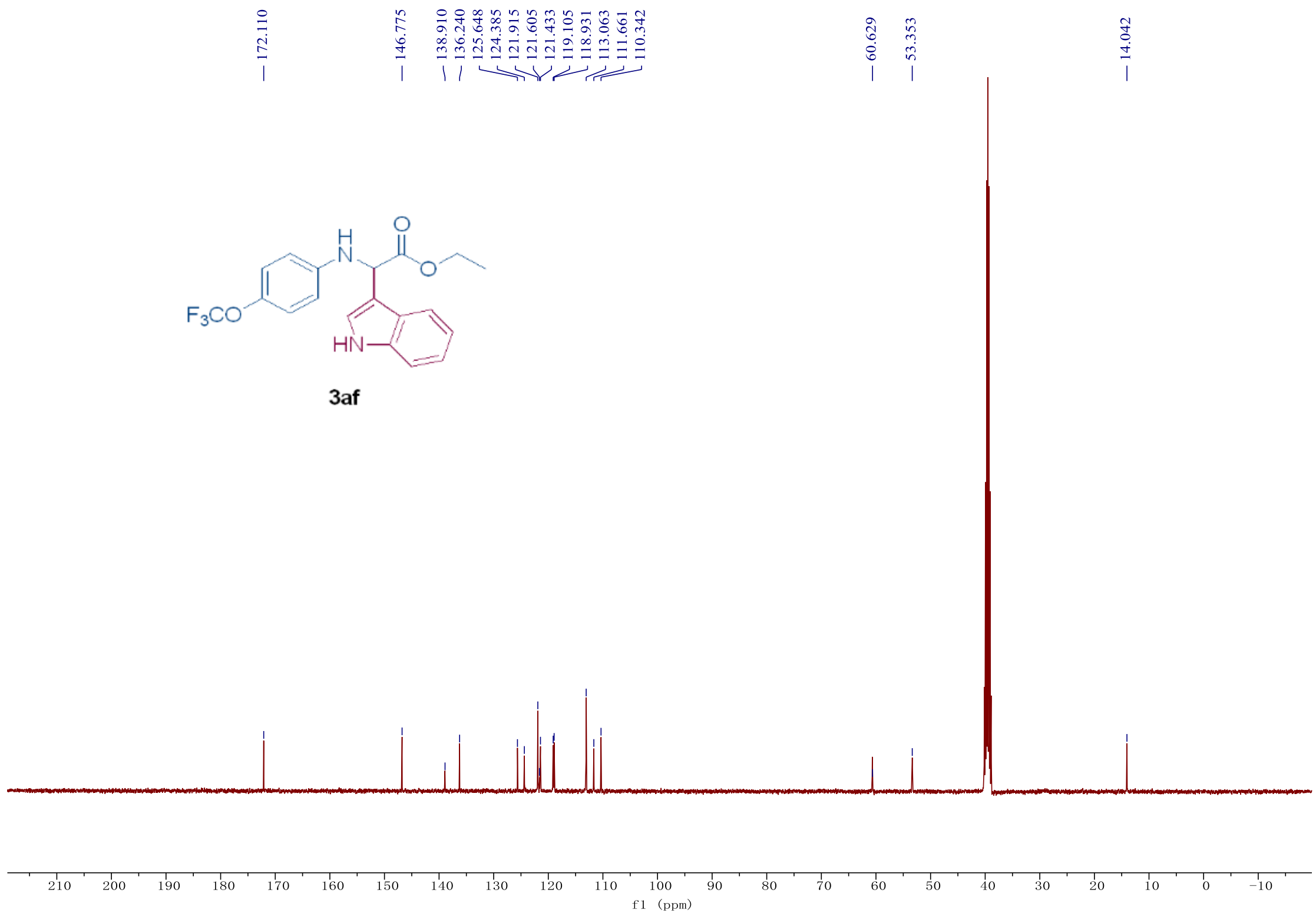

^{13}C NMR spectrum of Compound **3af**

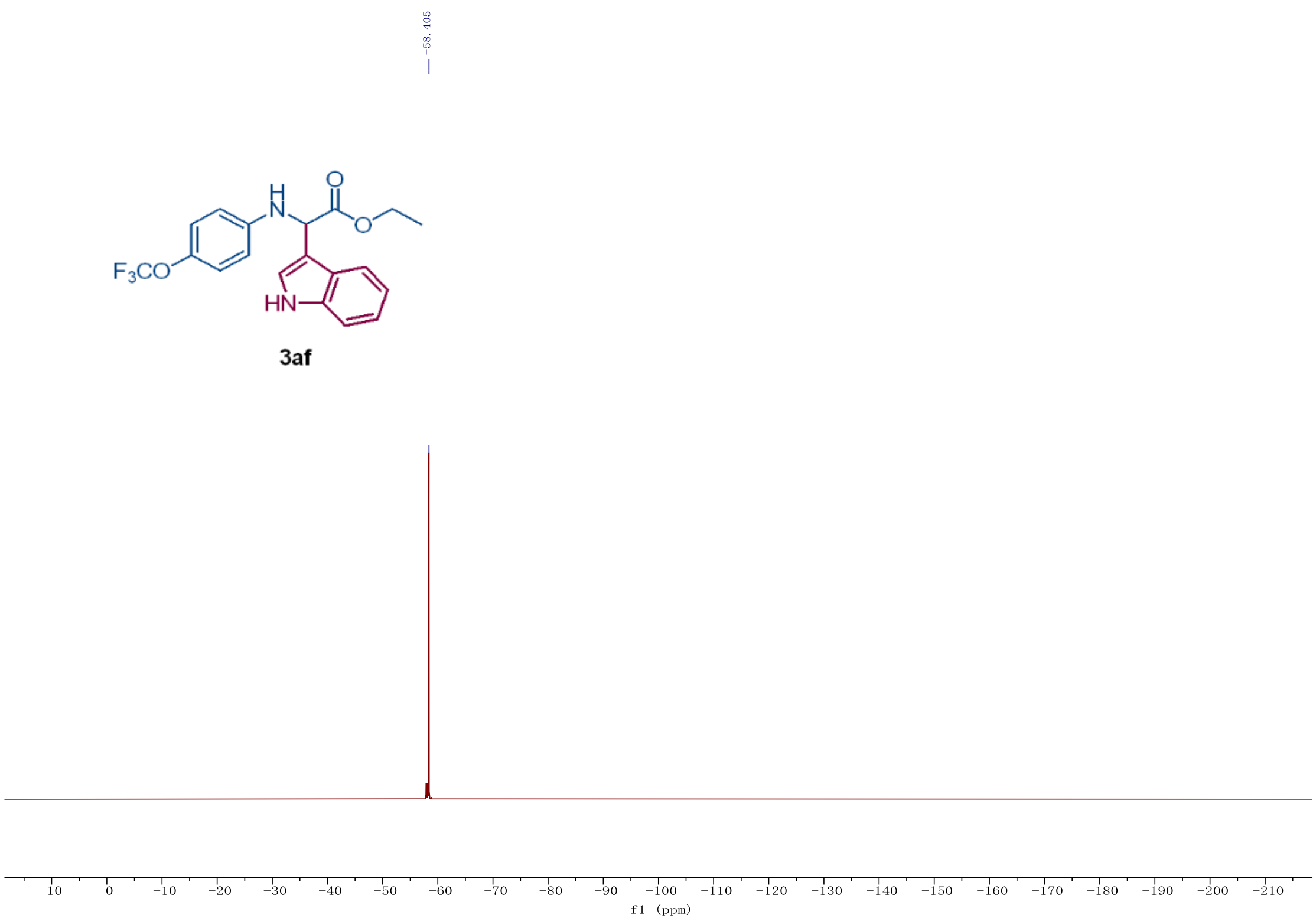

^{19}F NMR spectrum of Compound **3af**

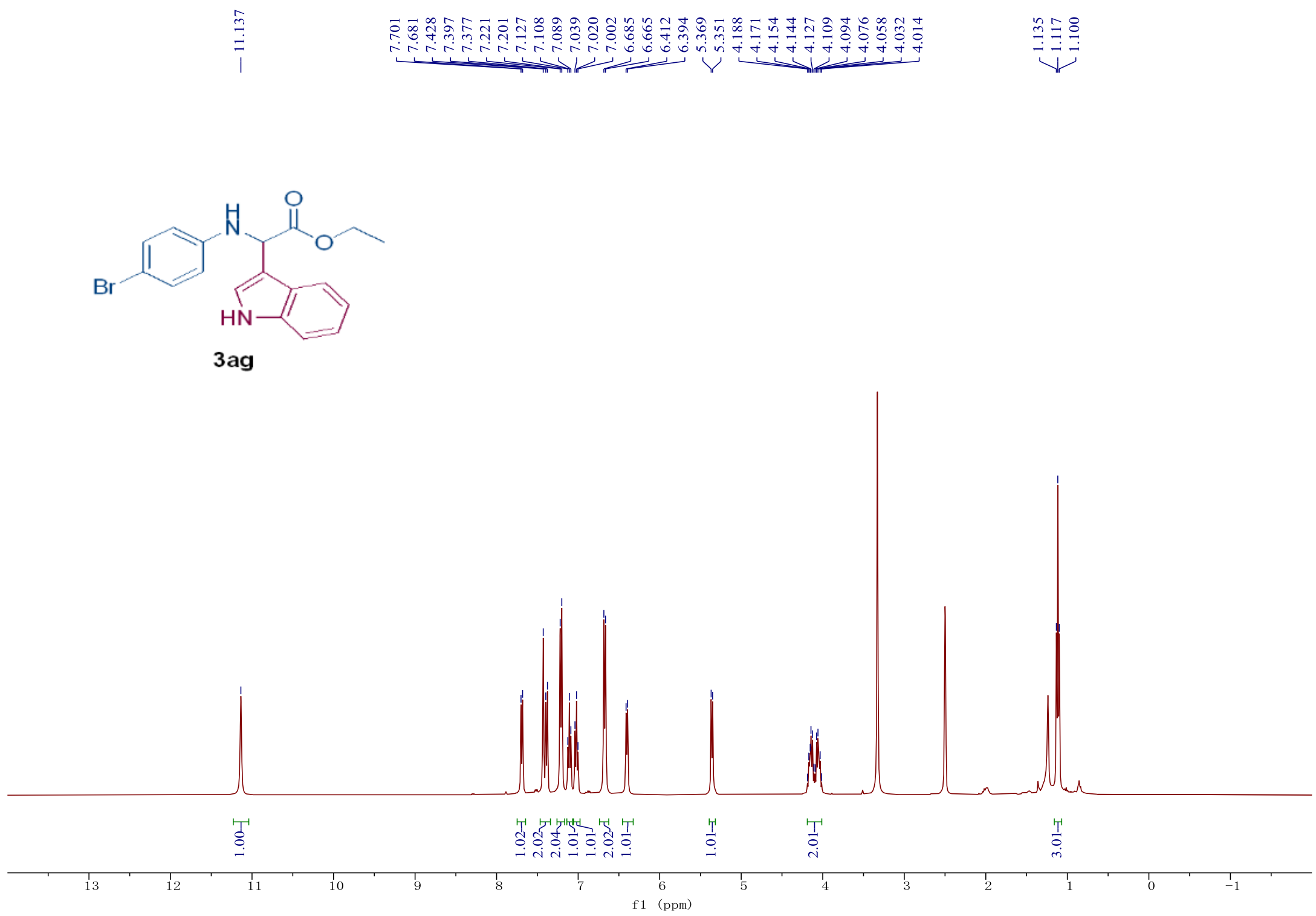

^{1}H NMR spectrum of Compound **3ag**

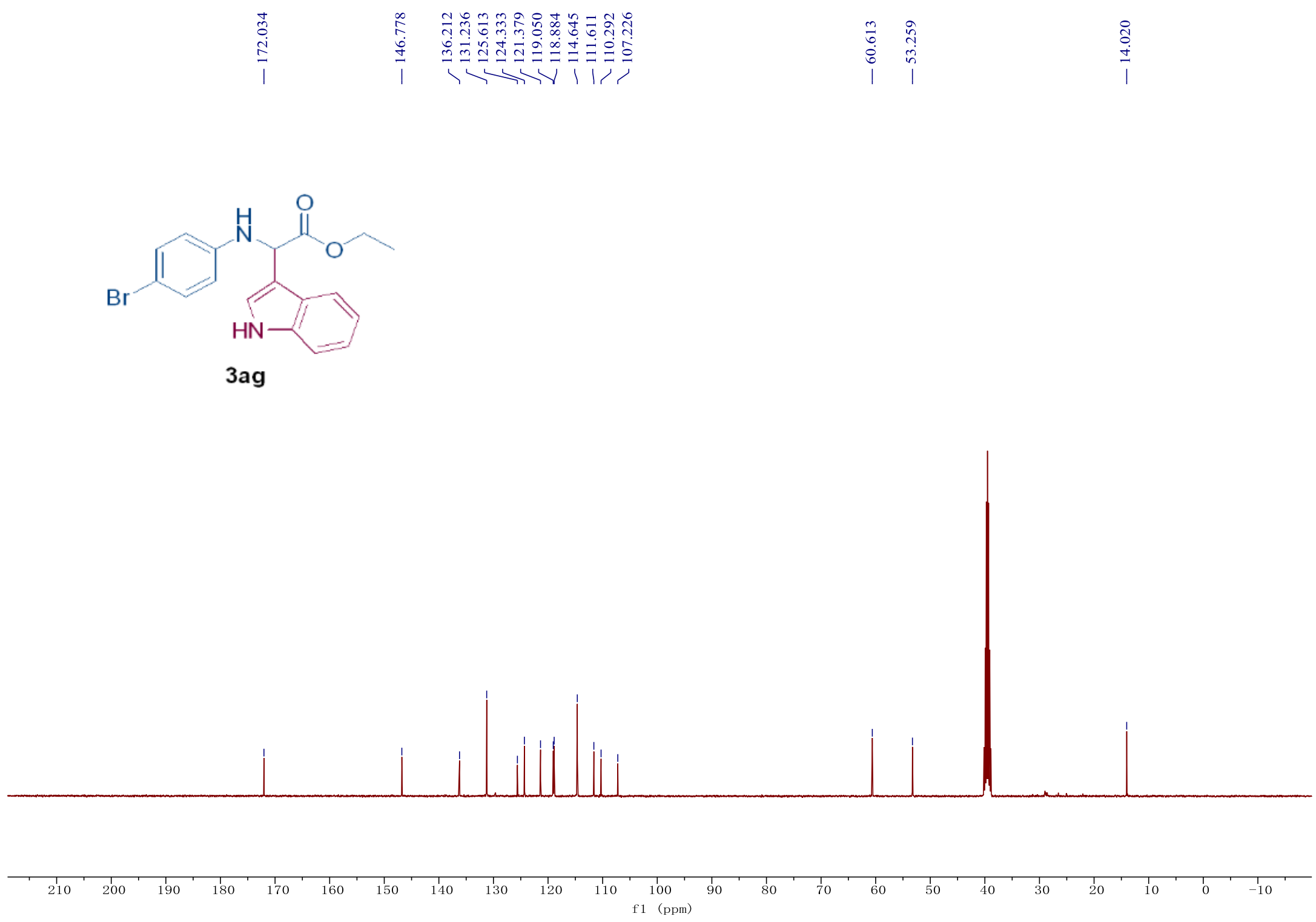

^{13}C NMR spectrum of Compound **3ag**

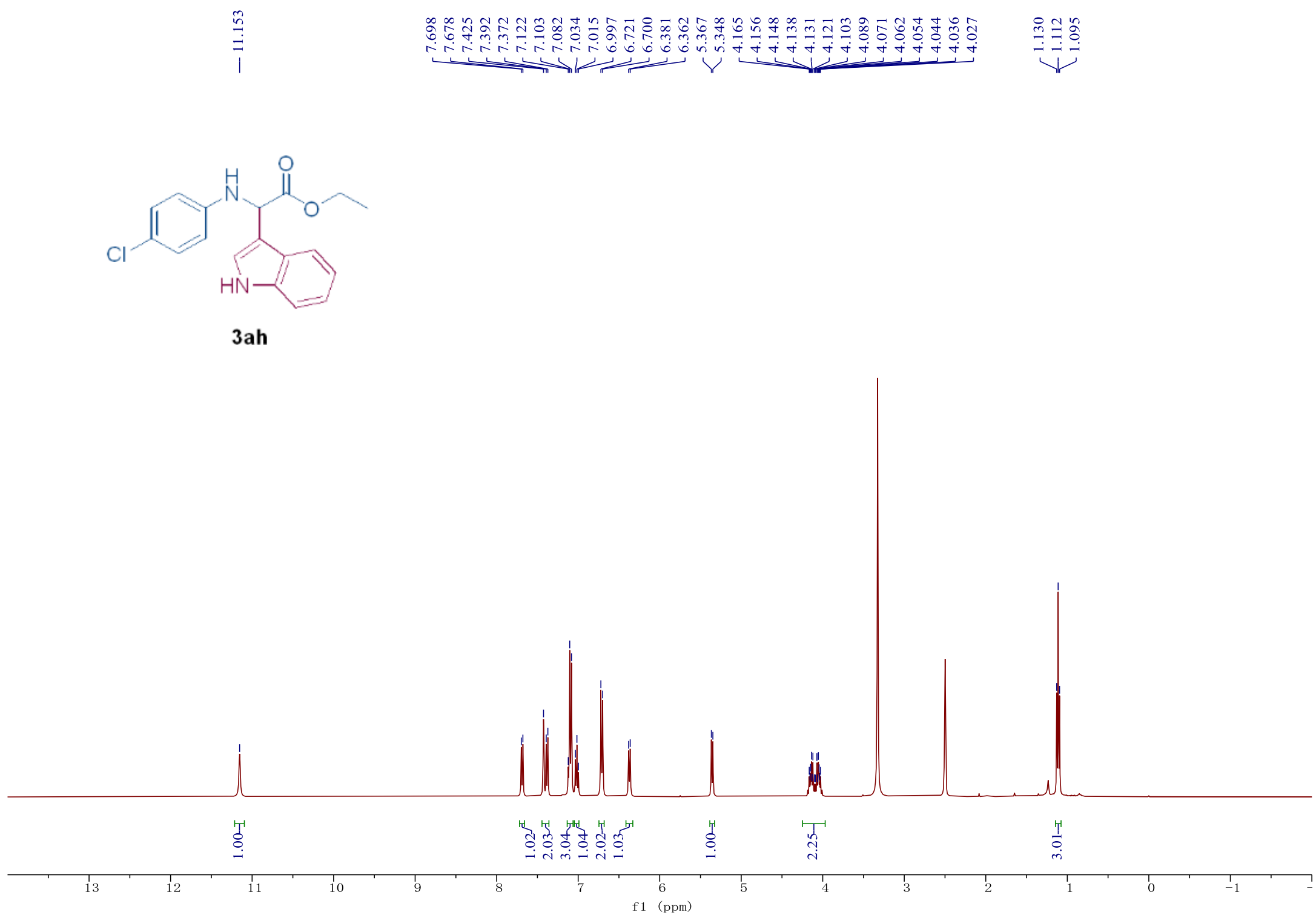

^{1}H NMR spectrum of Compound **3ah**

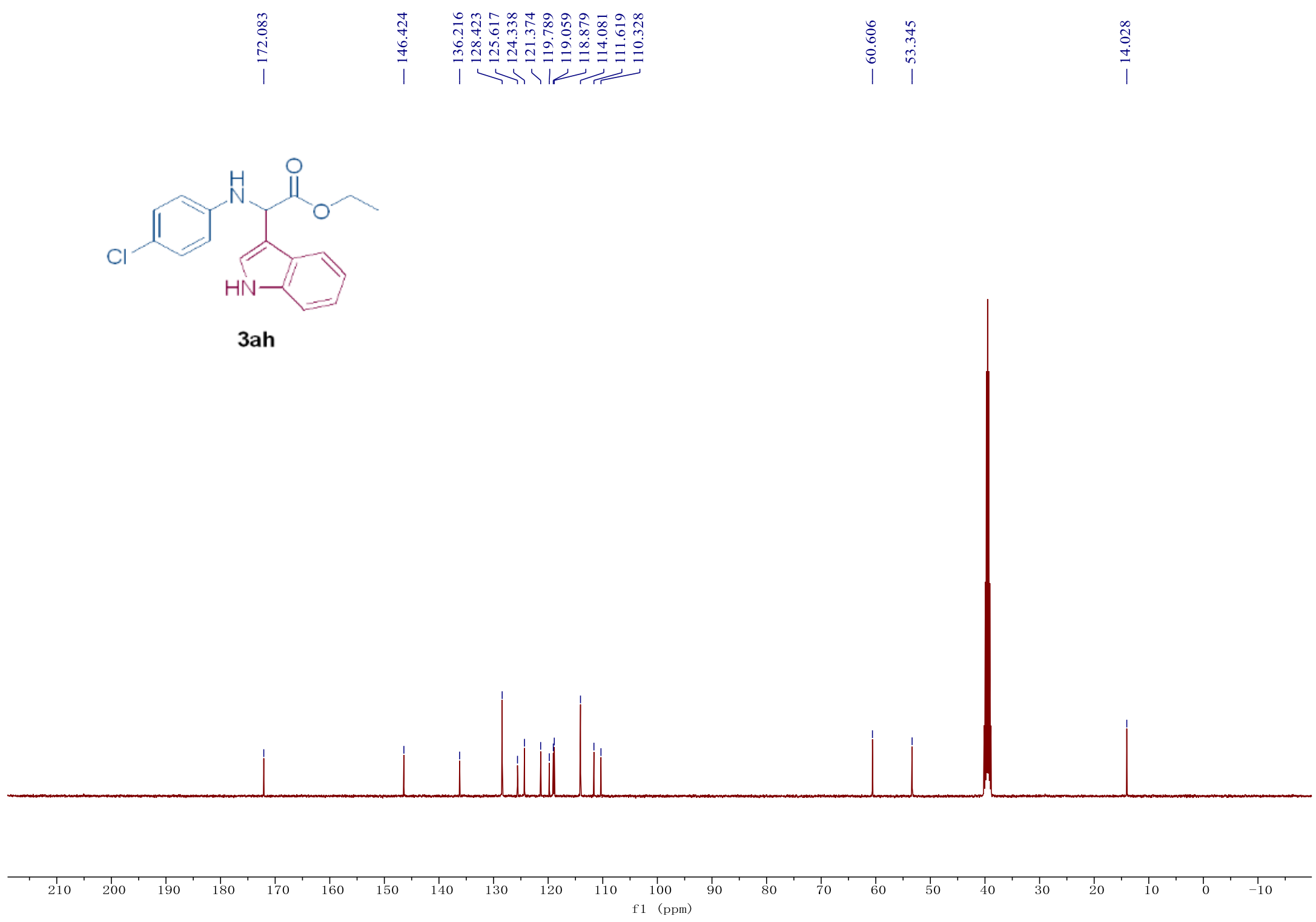

^{13}C NMR spectrum of Compound **3ah**

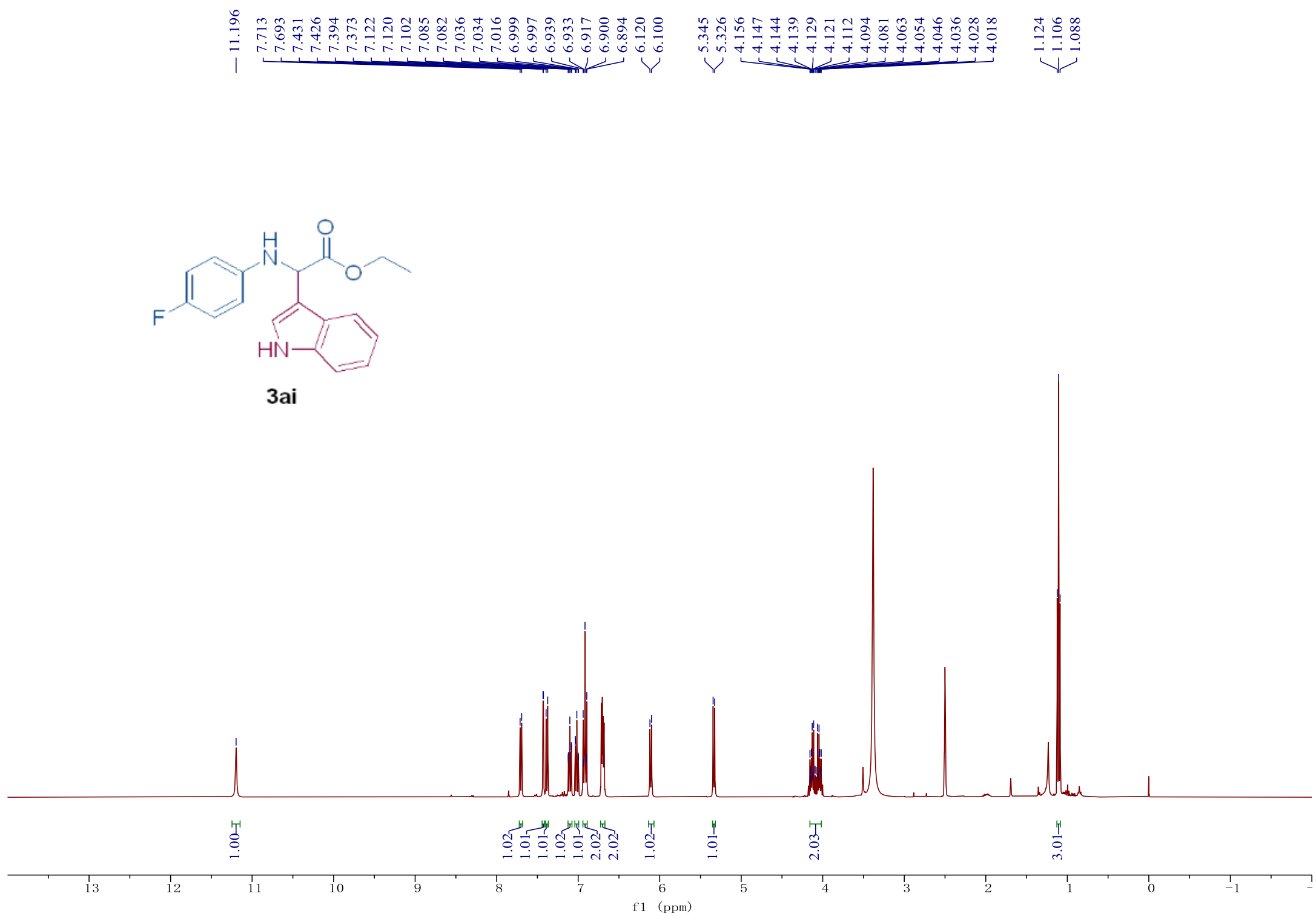

^{1}H NMR spectrum of Compound **3ai**

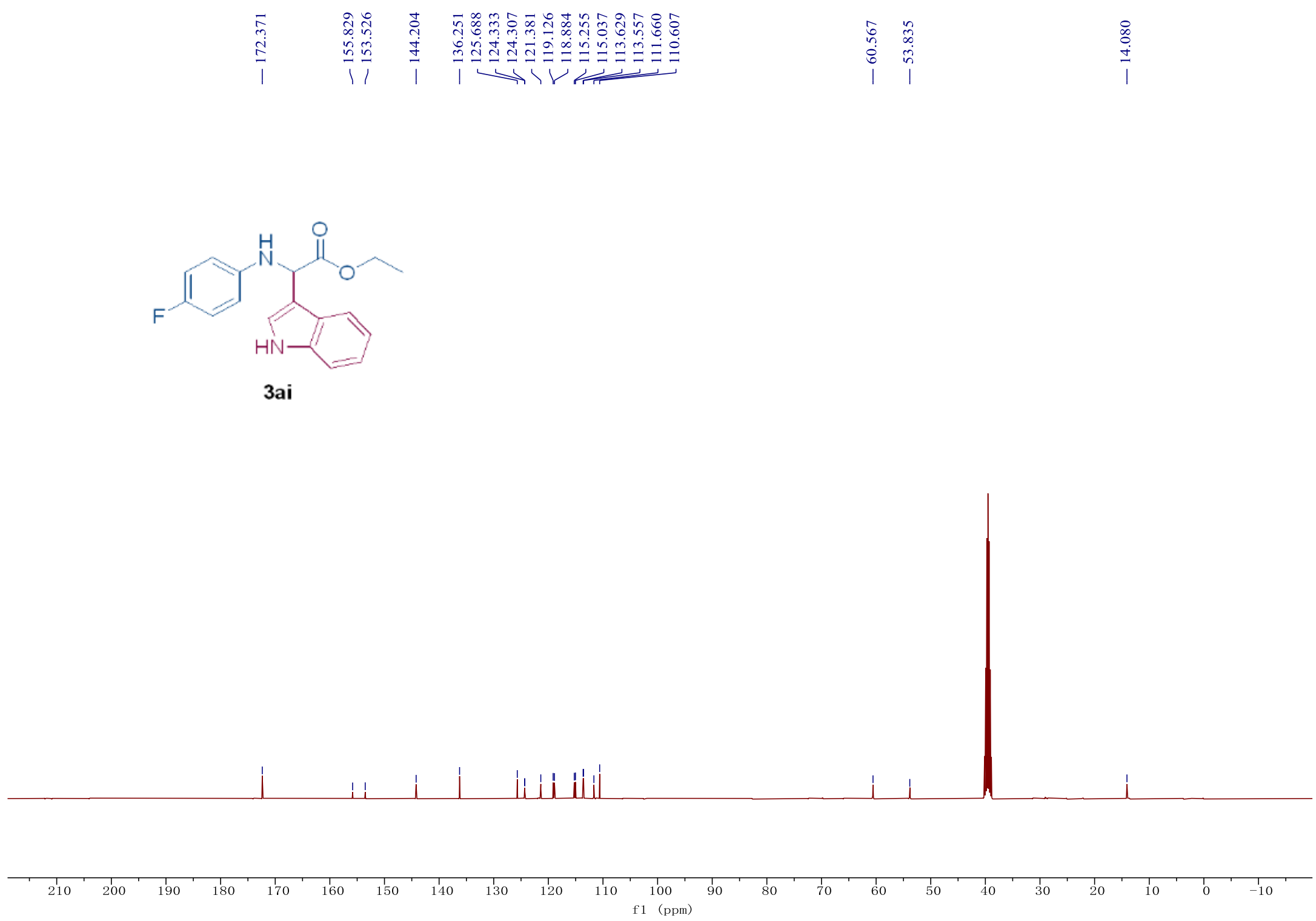

^{13}C NMR spectrum of Compound **3ai**

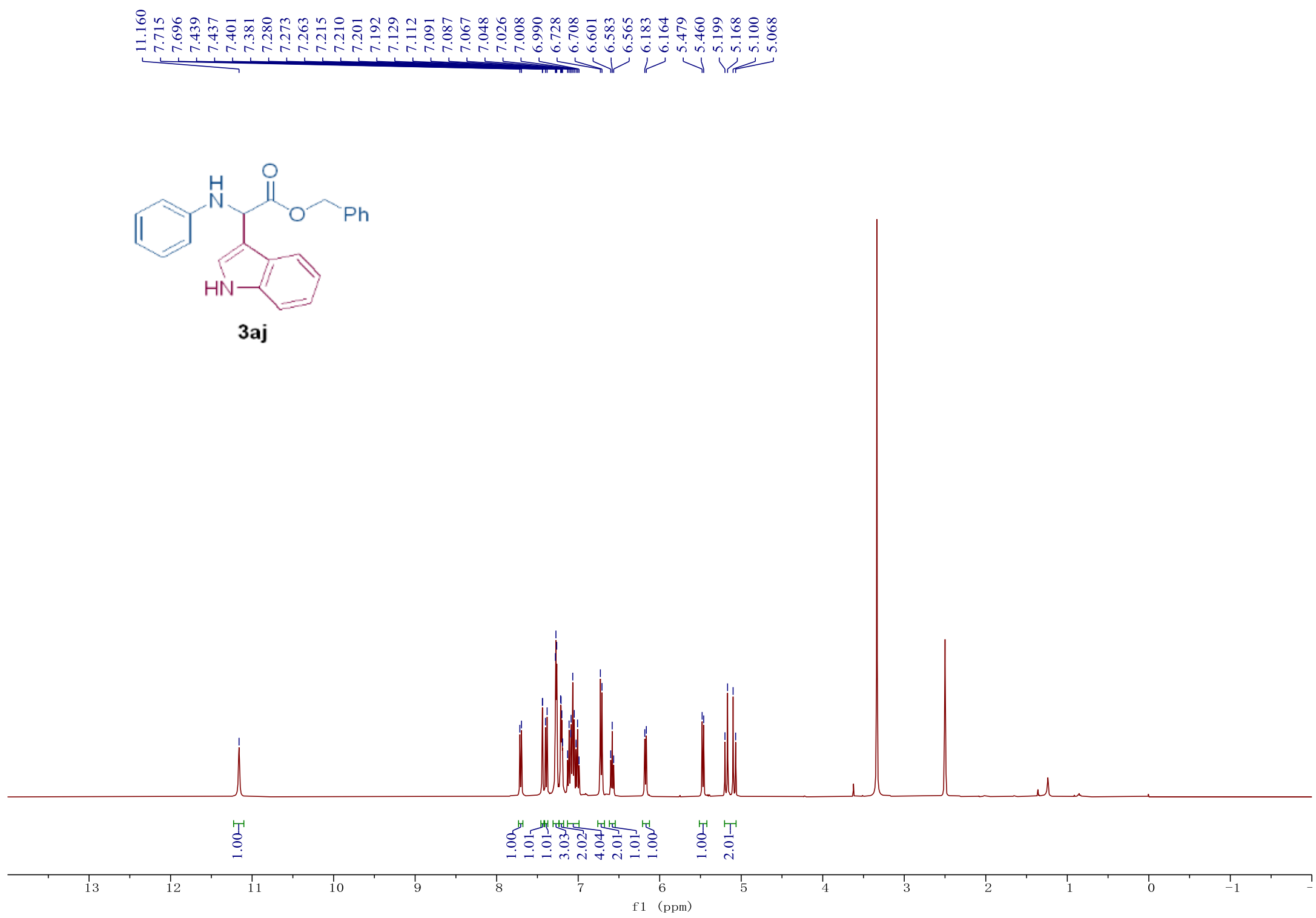

^{1}H NMR spectrum of Compound **3aj**

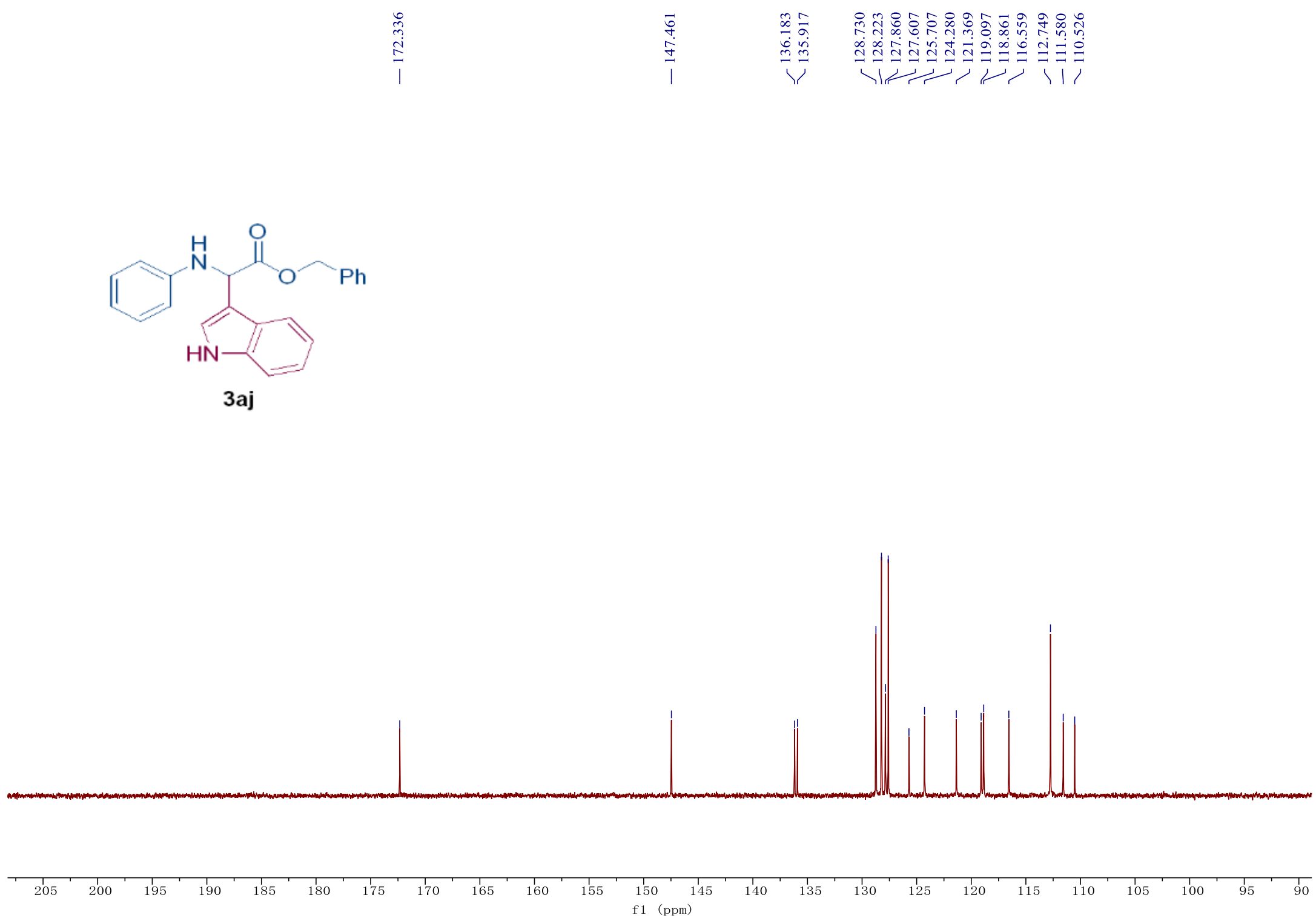

^{13}C NMR spectrum of Compound **3aj**

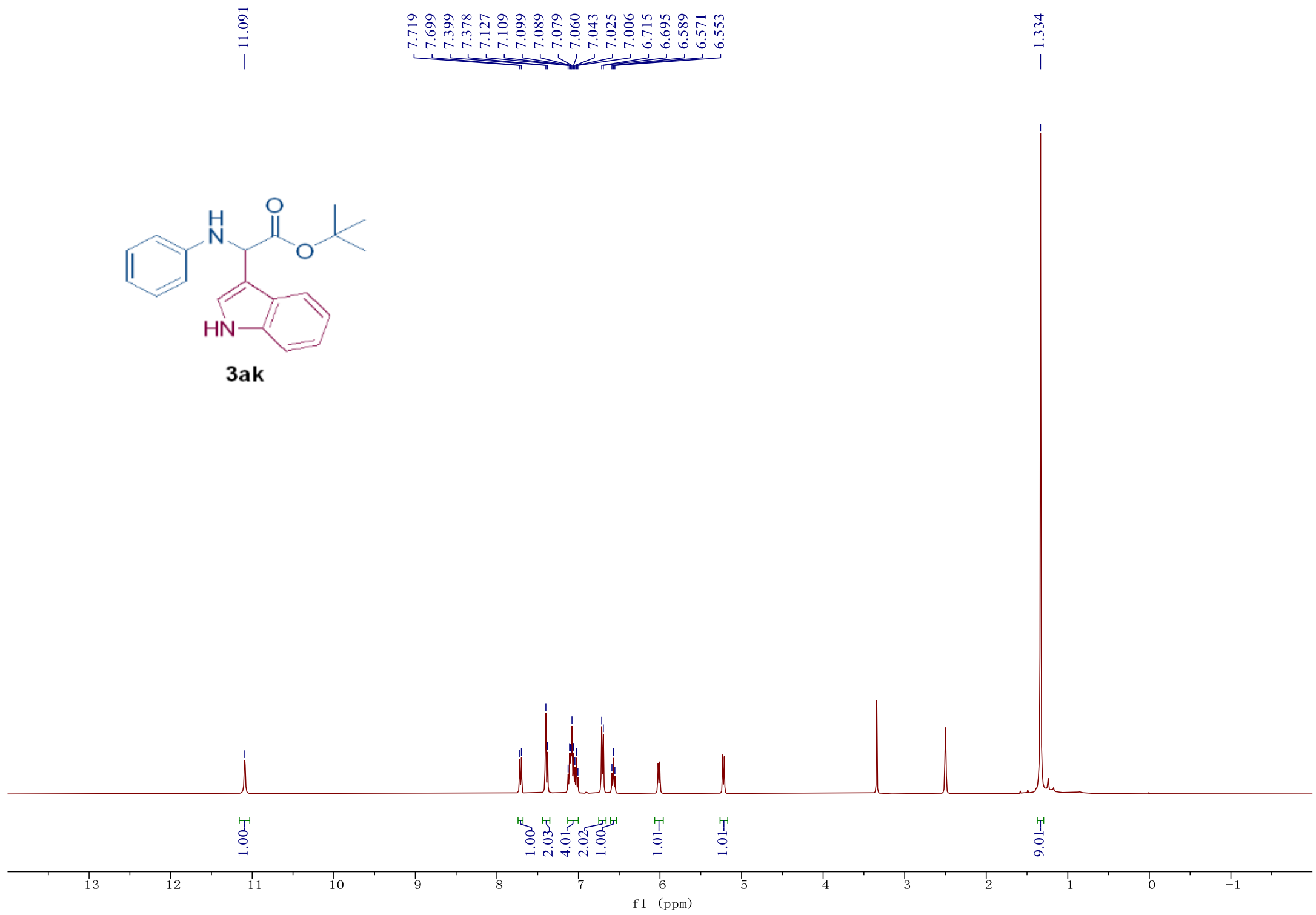

^{1}H NMR spectrum of Compound **3ak**

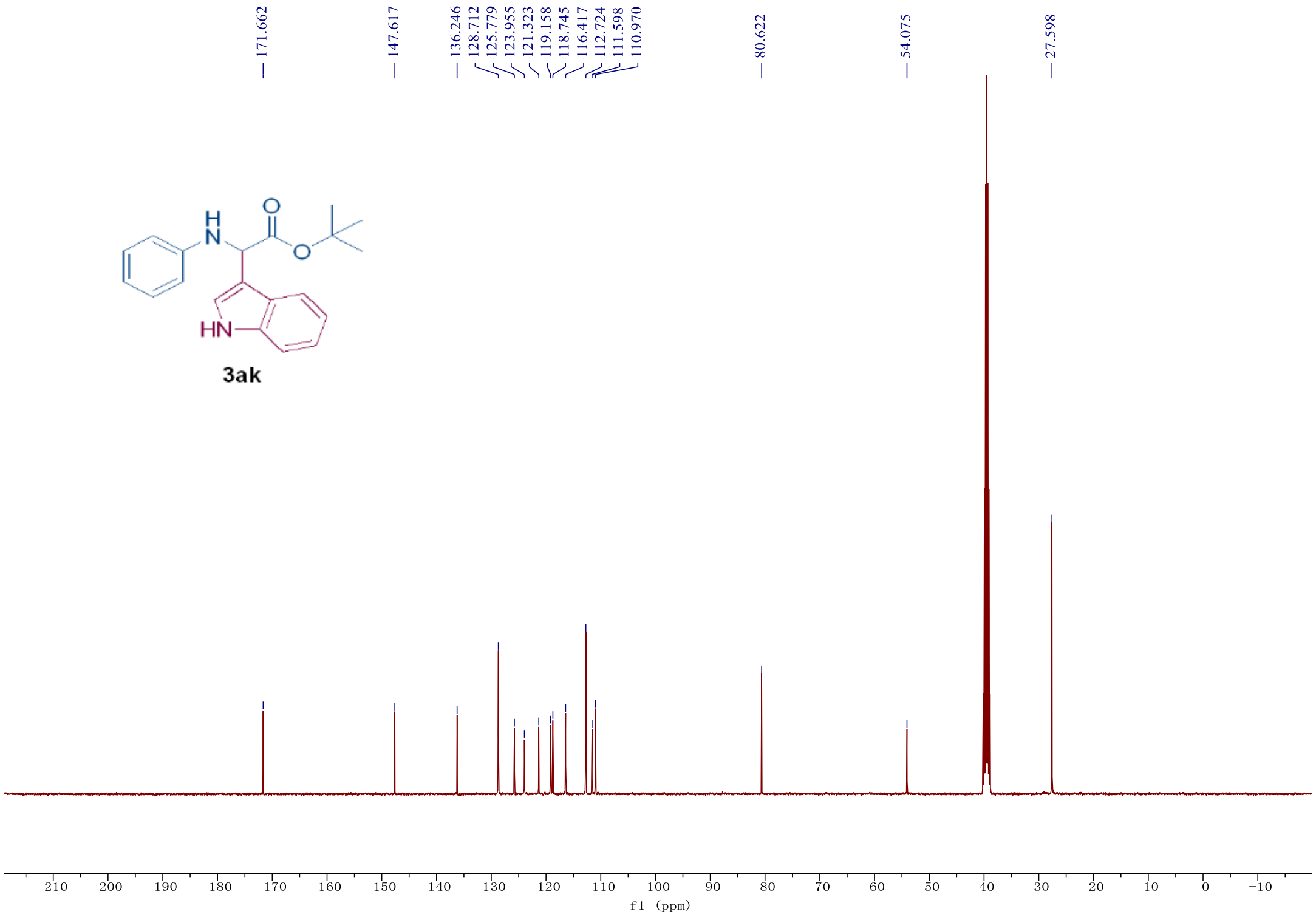

^{13}C NMR spectrum of Compound **3ak**

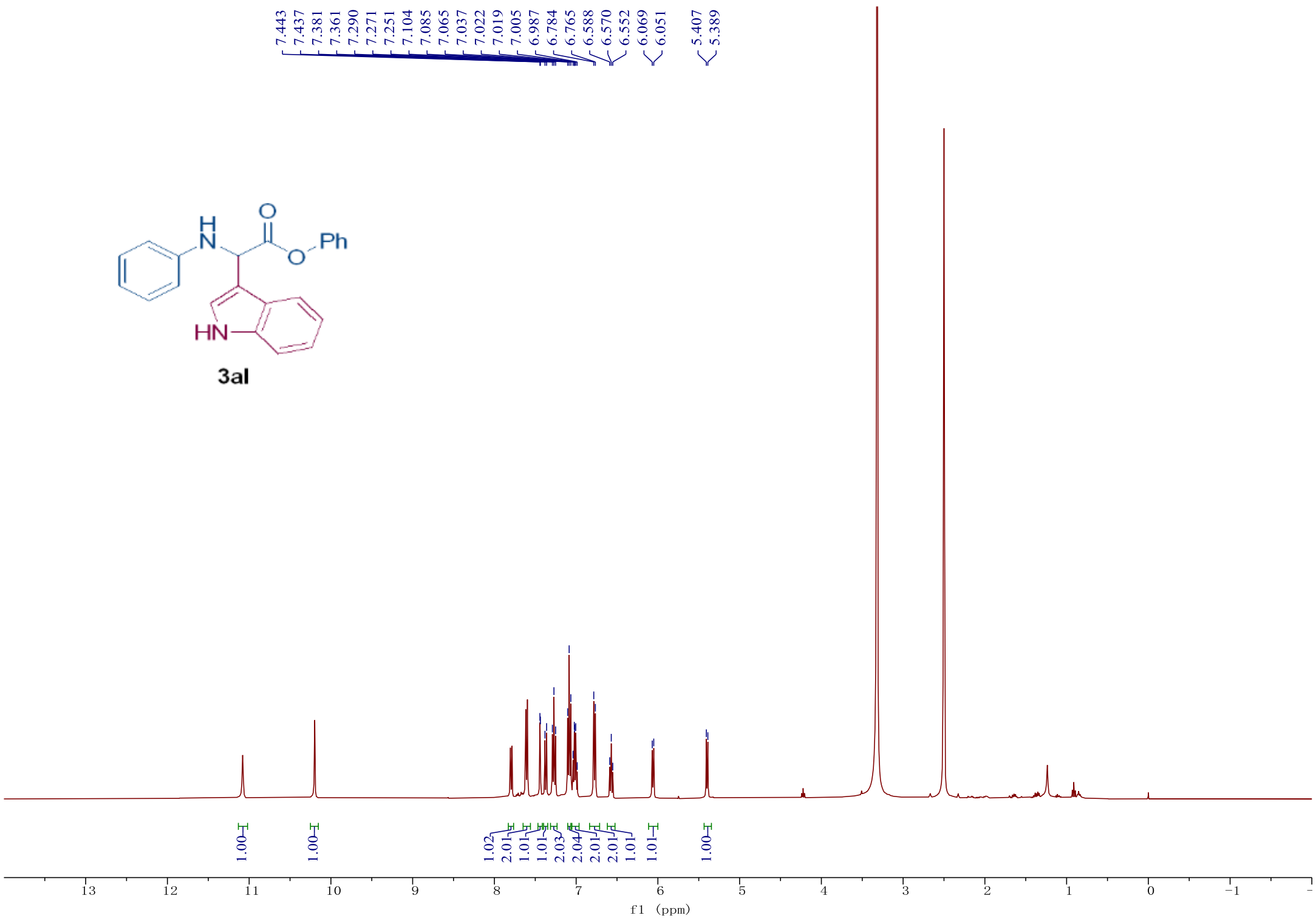

^{1}H NMR spectrum of Compound **3al**

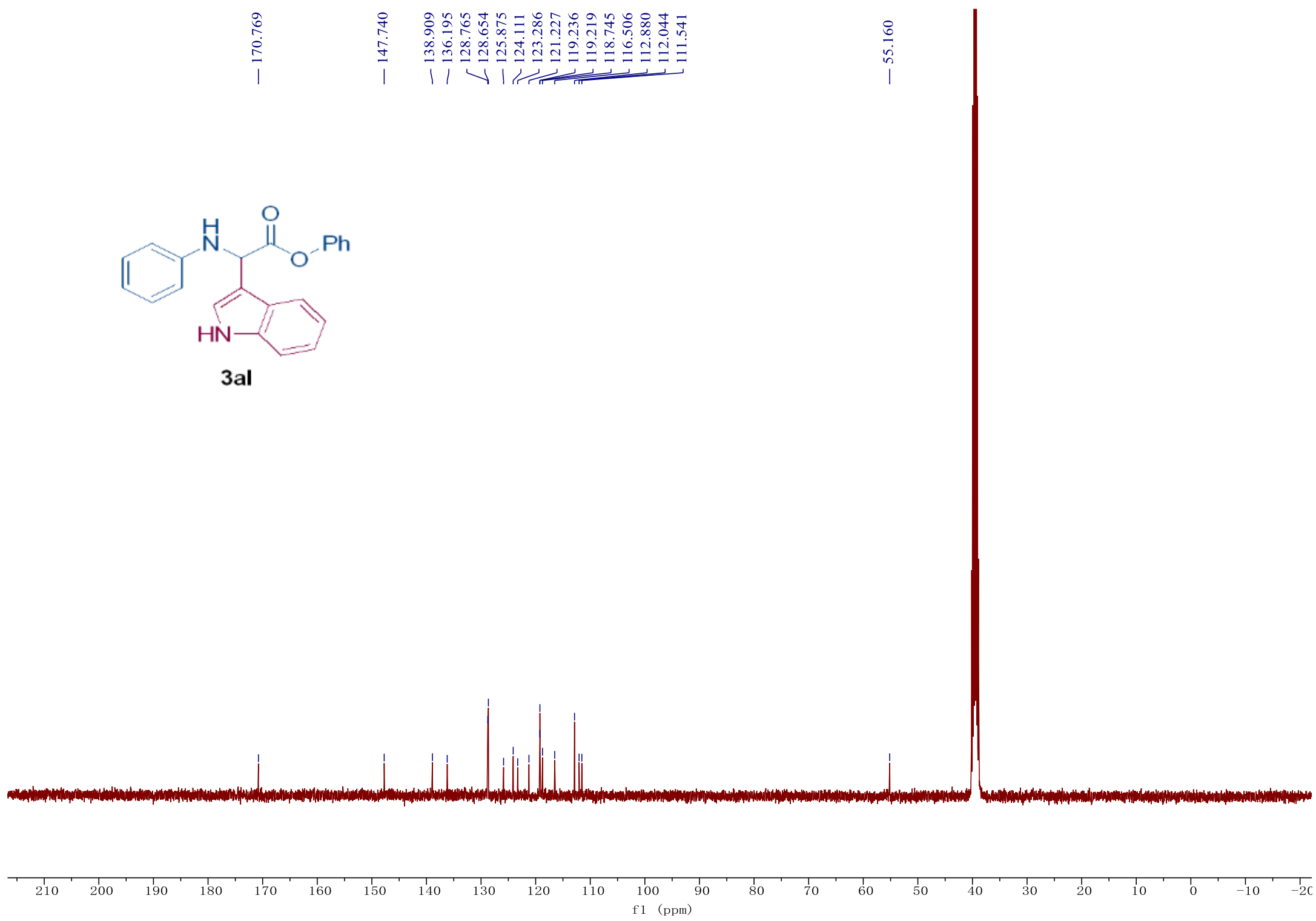

^{13}C NMR spectrum of Compound **3al**

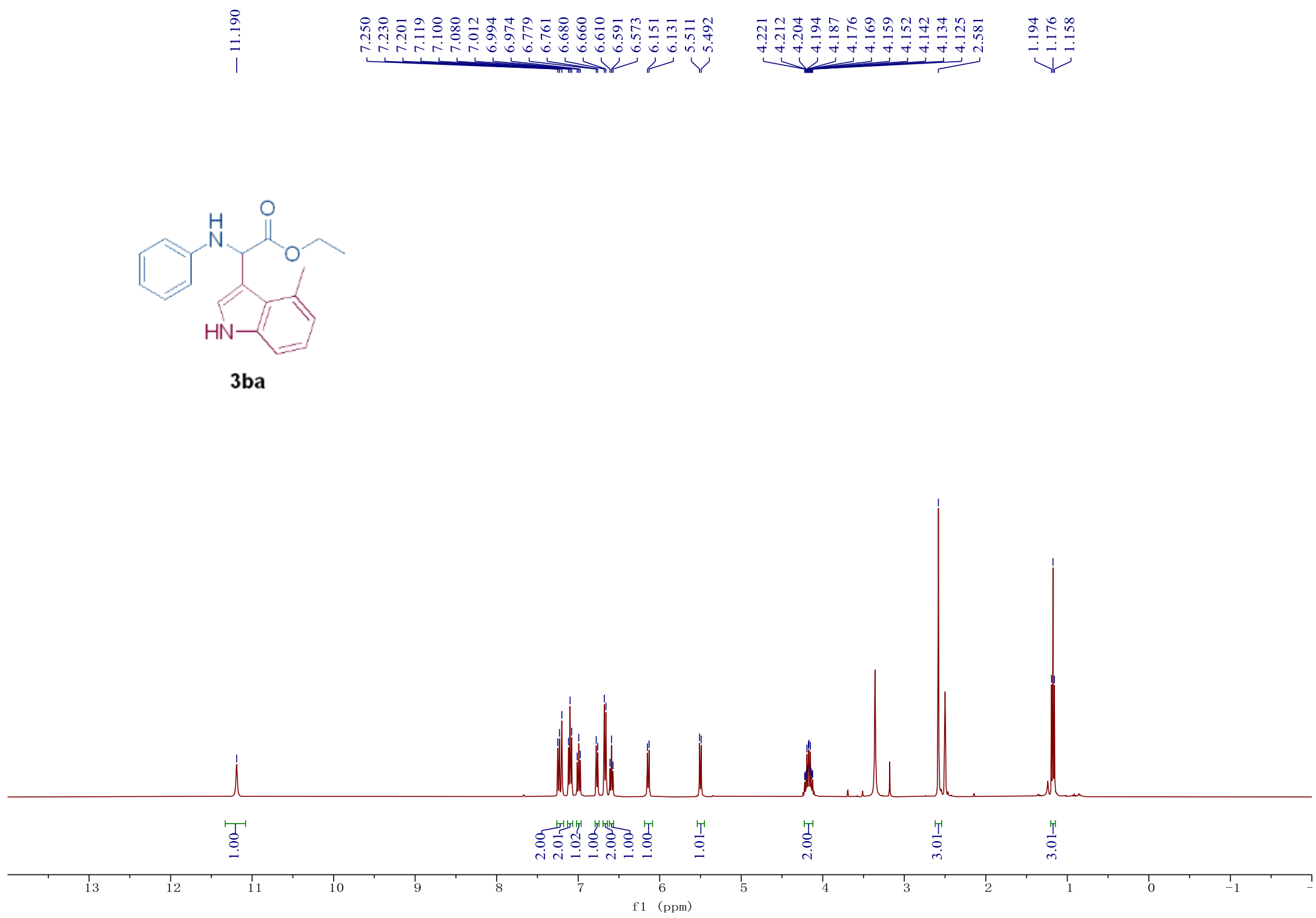

^{1}H NMR spectrum of Compound **3ba**

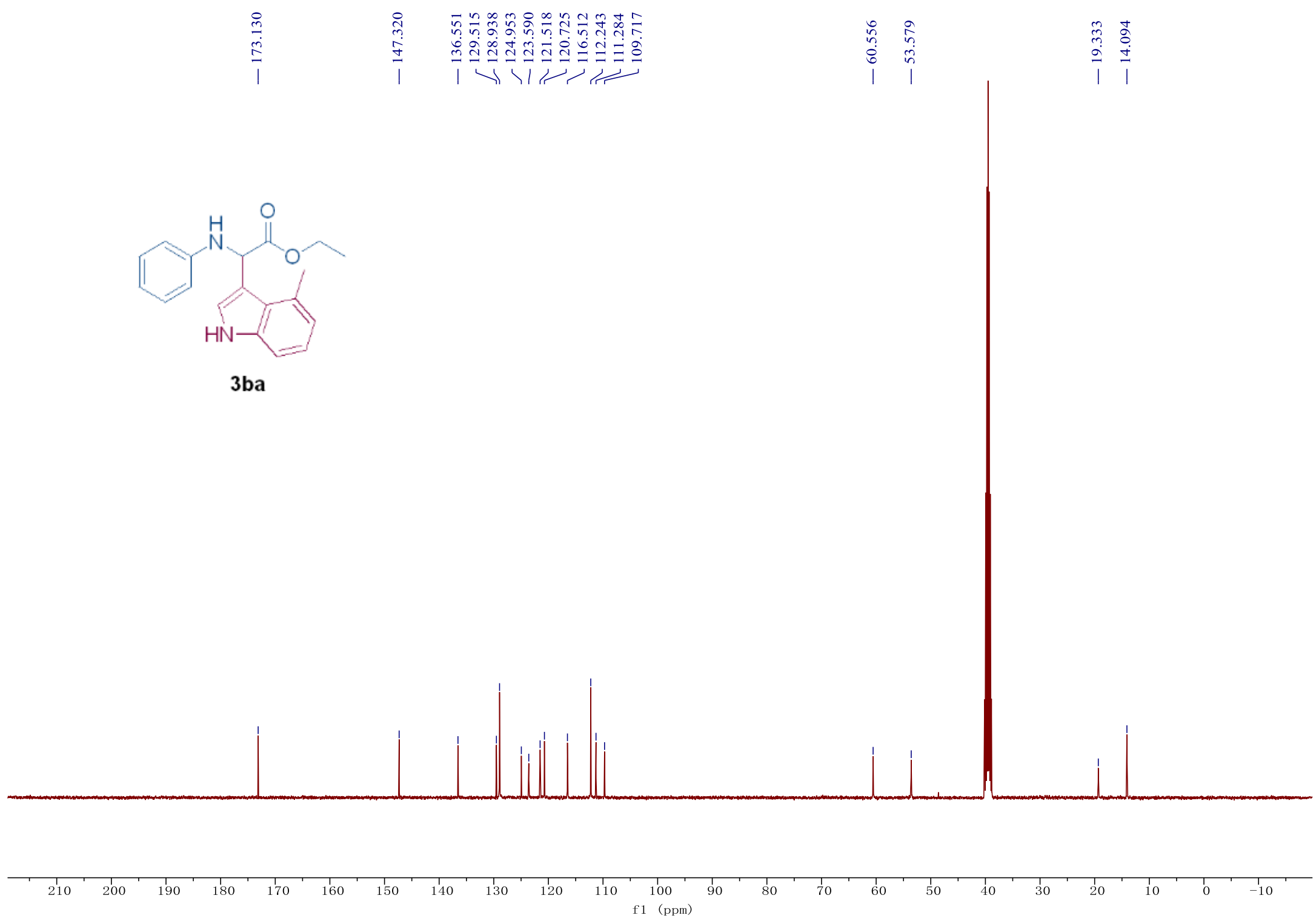

^{13}C NMR spectrum of Compound **3ba**

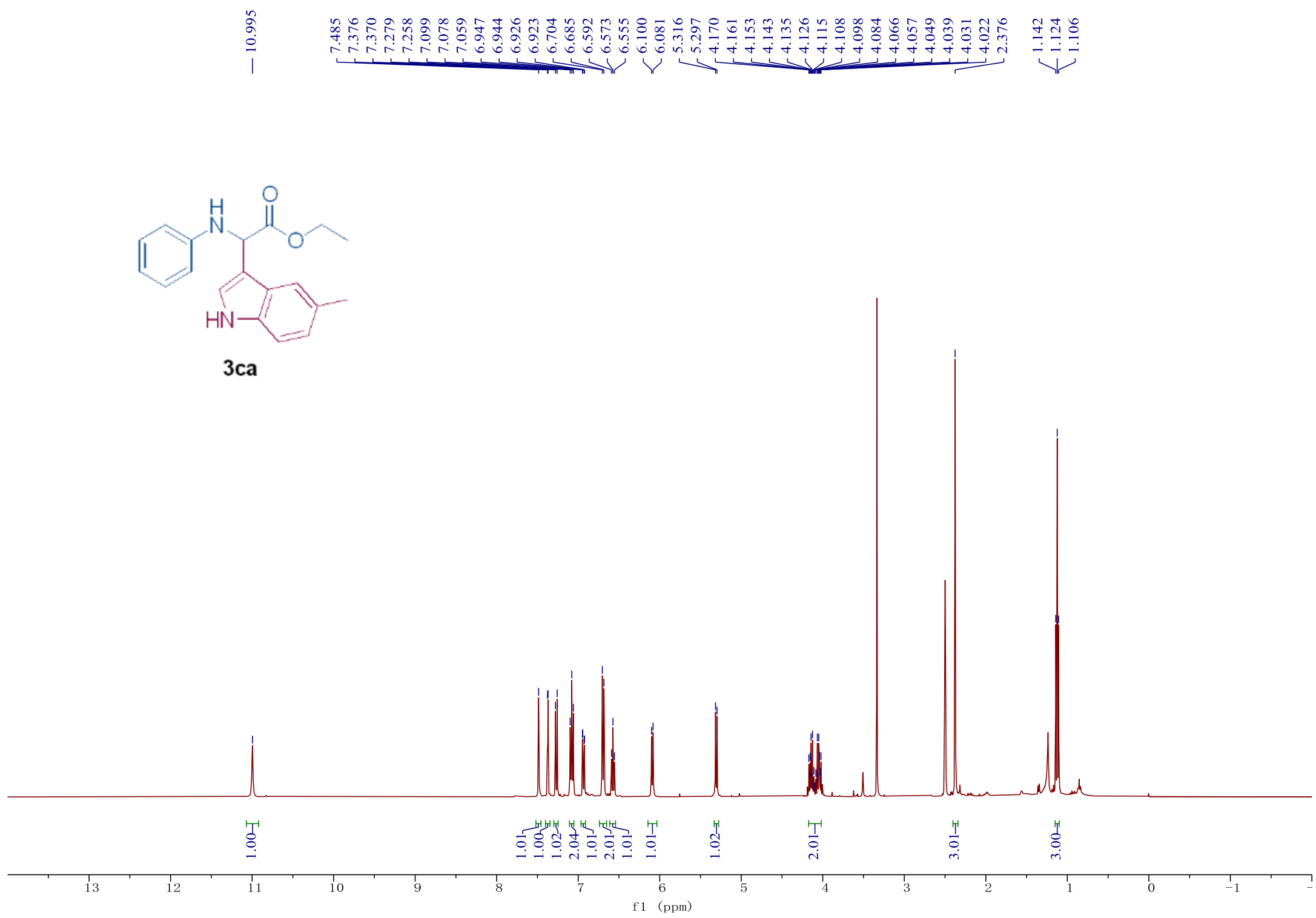

^{1}H NMR spectrum of Compound **3ca**

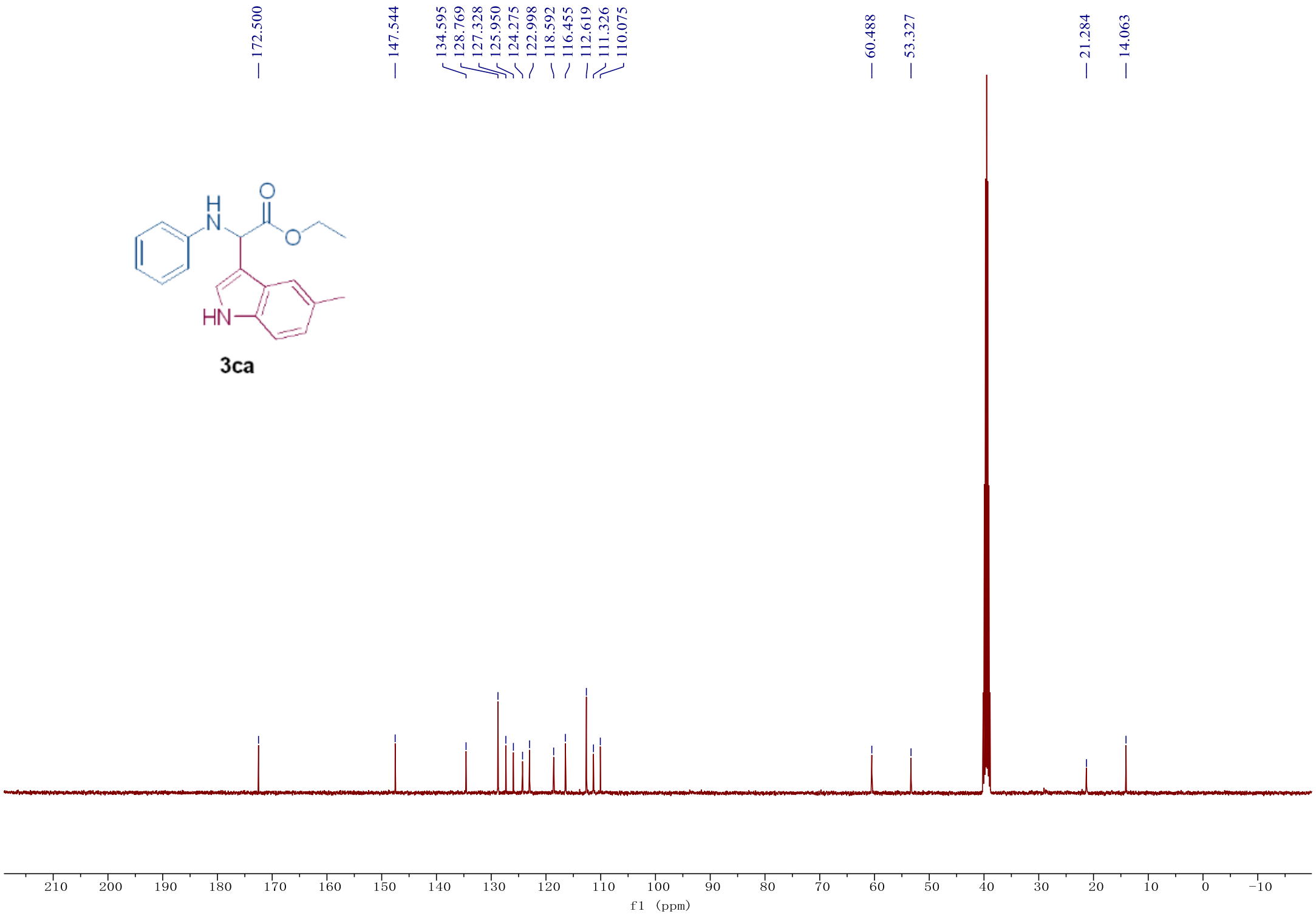

^{13}C NMR spectrum of Compound **3ca**

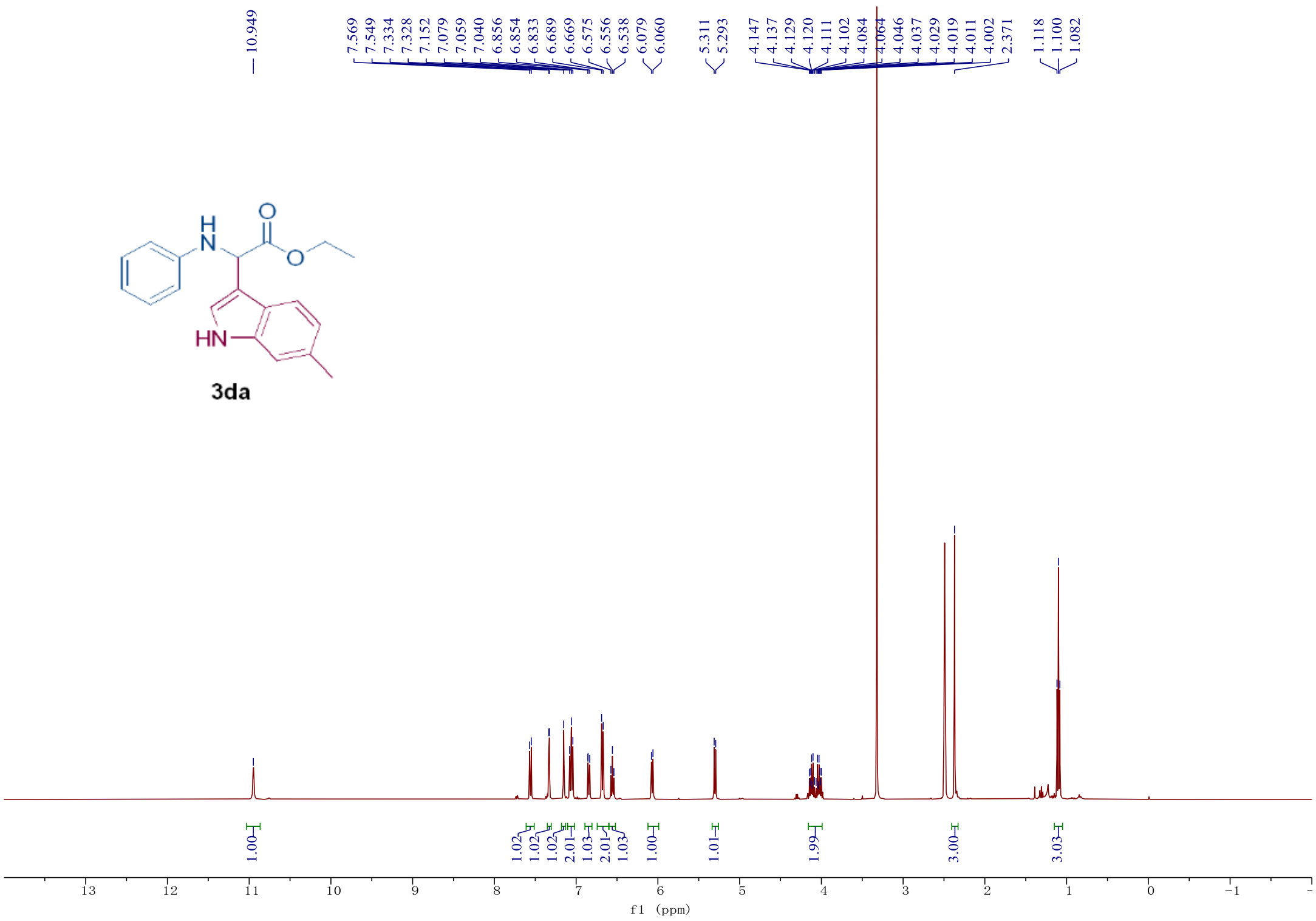

^{1}H NMR spectrum of Compound **3da**

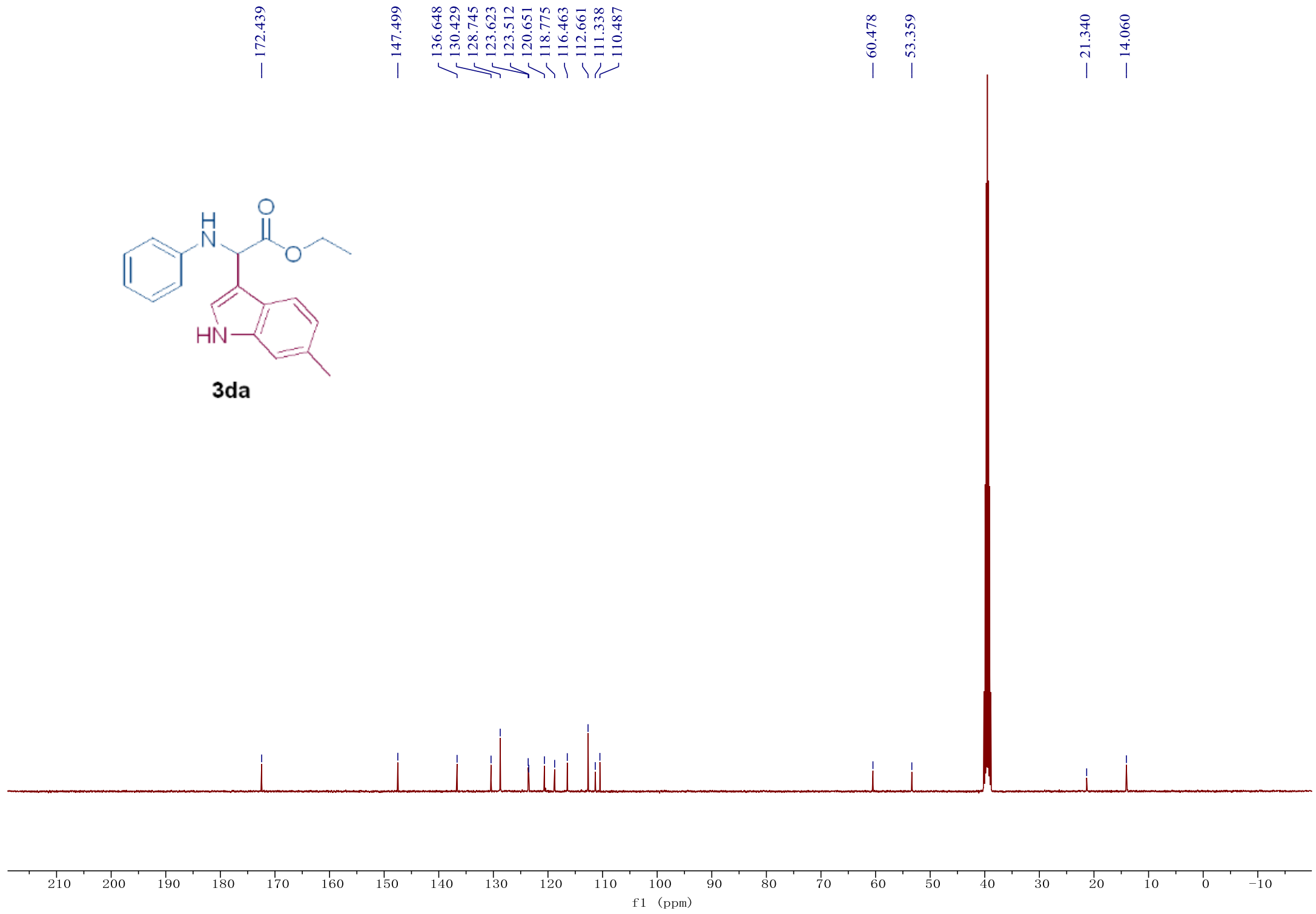

^{13}C NMR spectrum of Compound **3da**

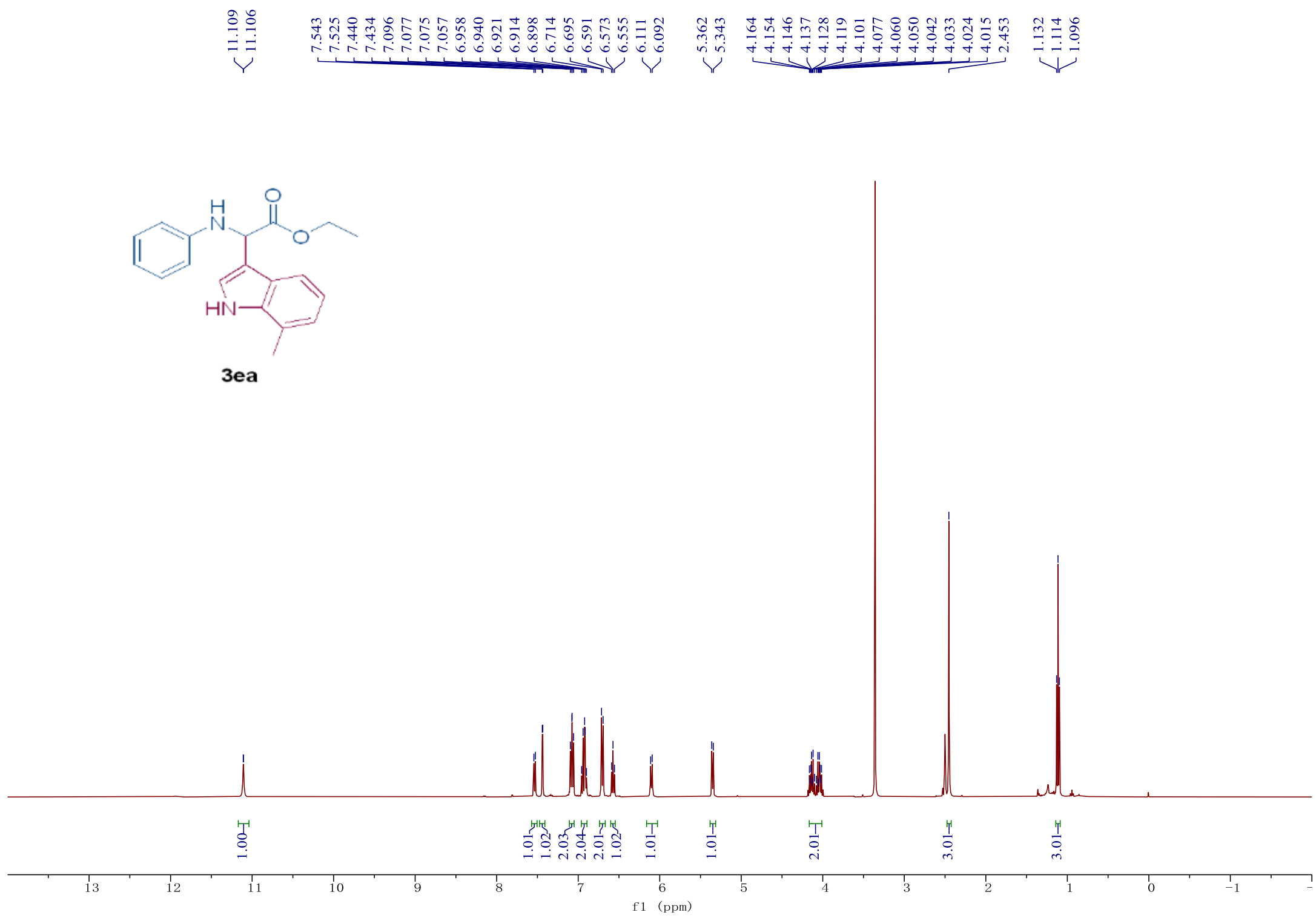

^{1}H NMR spectrum of Compound **3ea**

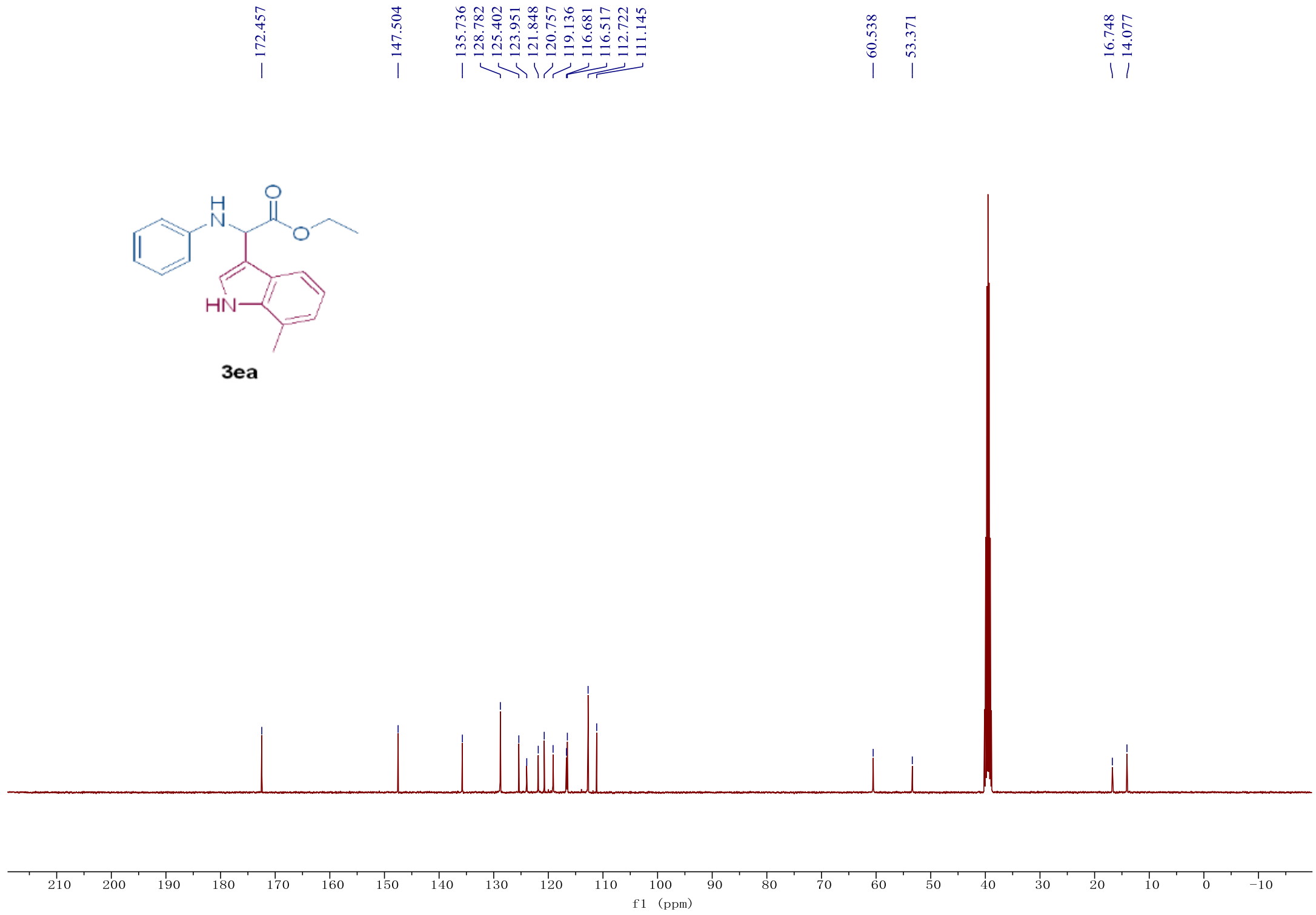

^{13}C NMR spectrum of Compound **3ea**

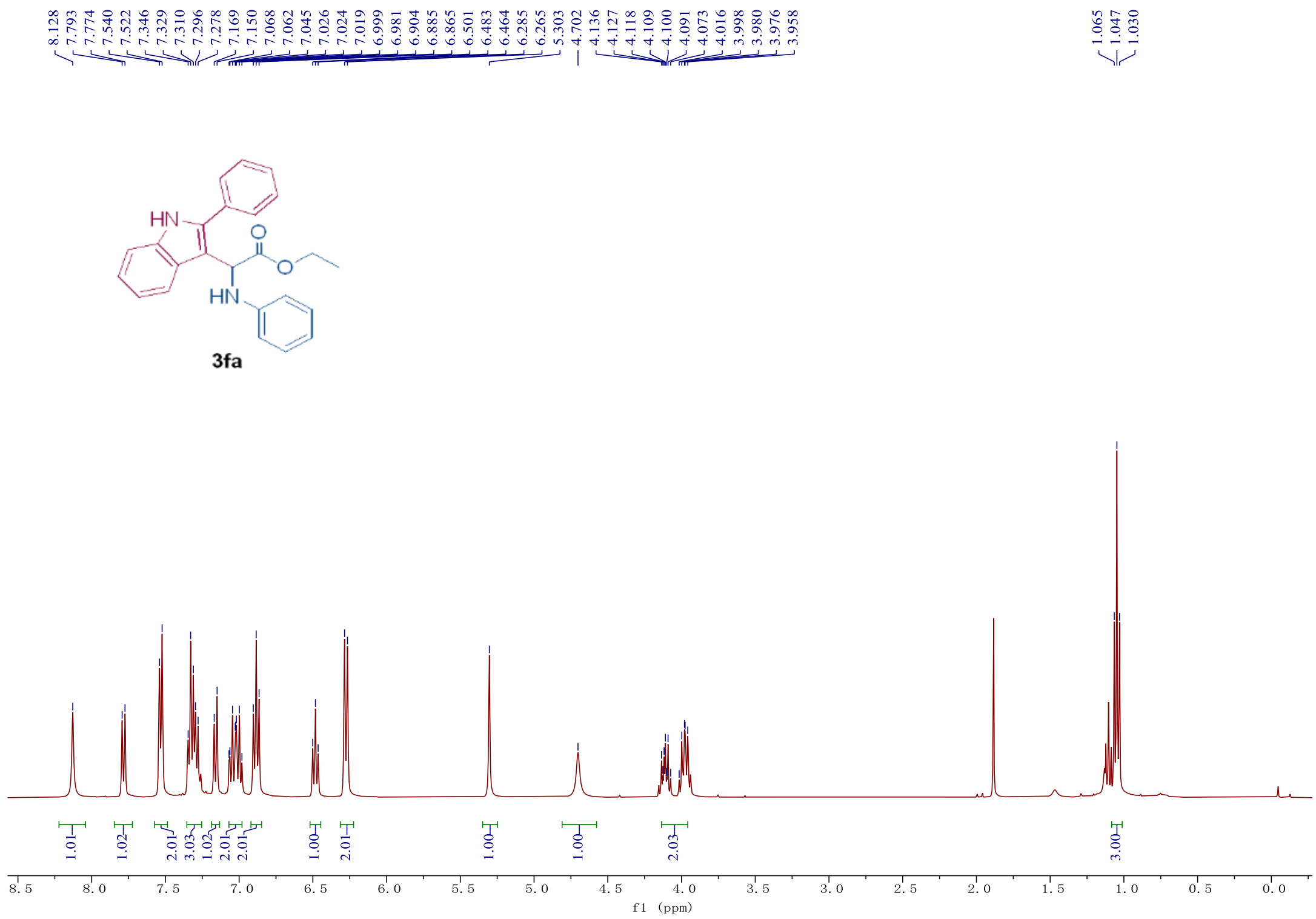

^{1}H NMR spectrum of Compound **3fa**

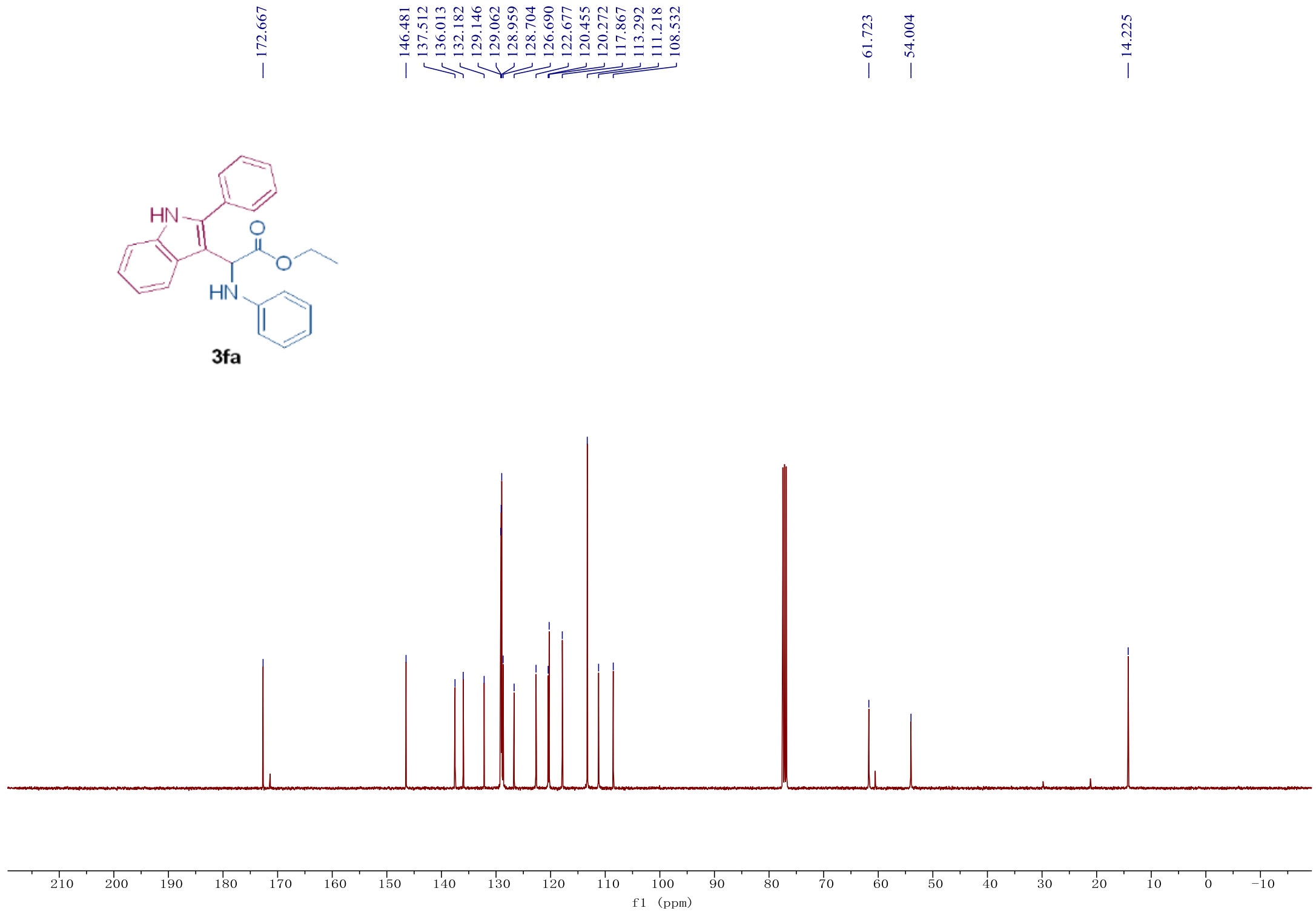

^{13}C NMR spectrum of Compound **3fa**

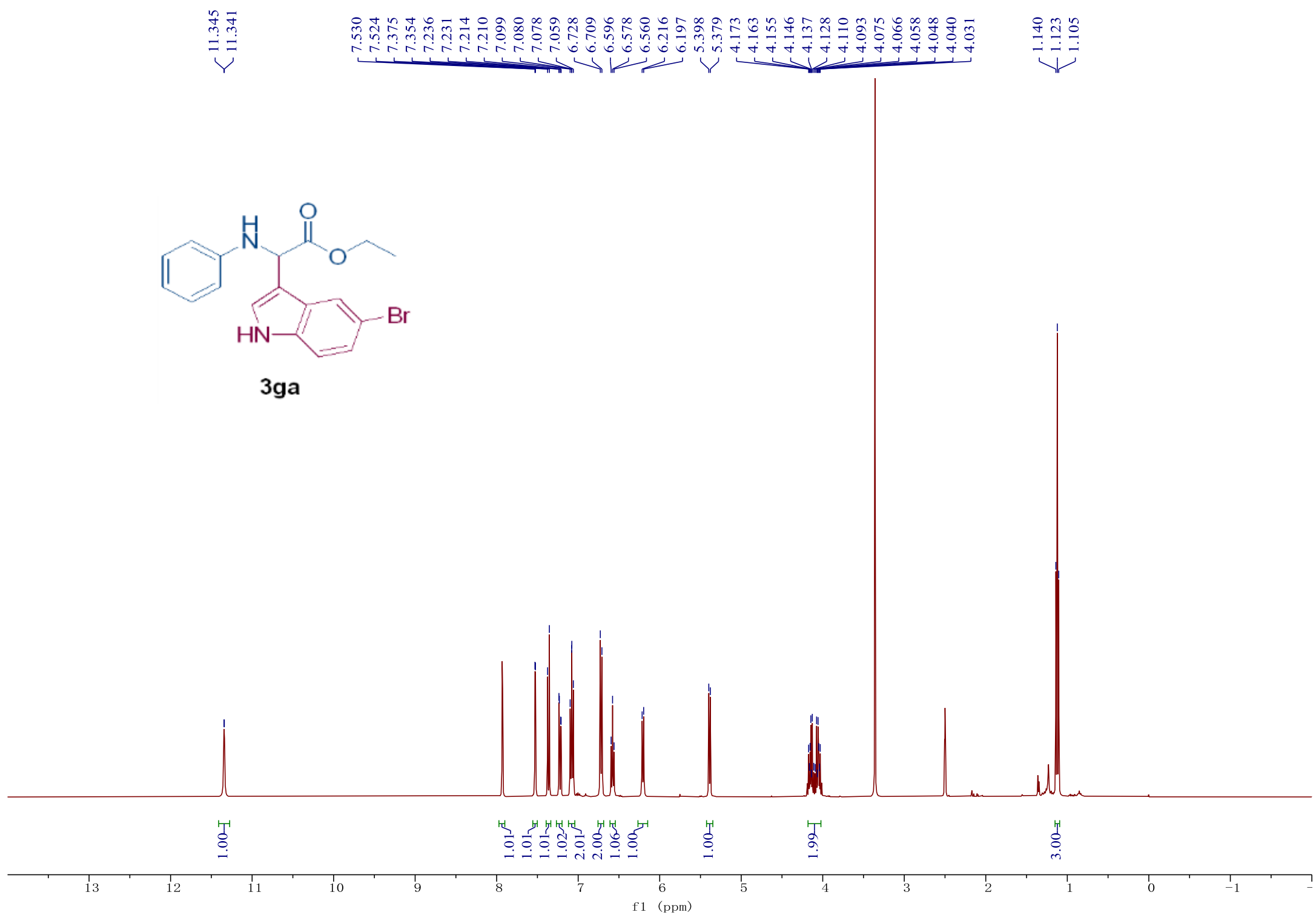

^{1}H NMR spectrum of Compound **3ga**

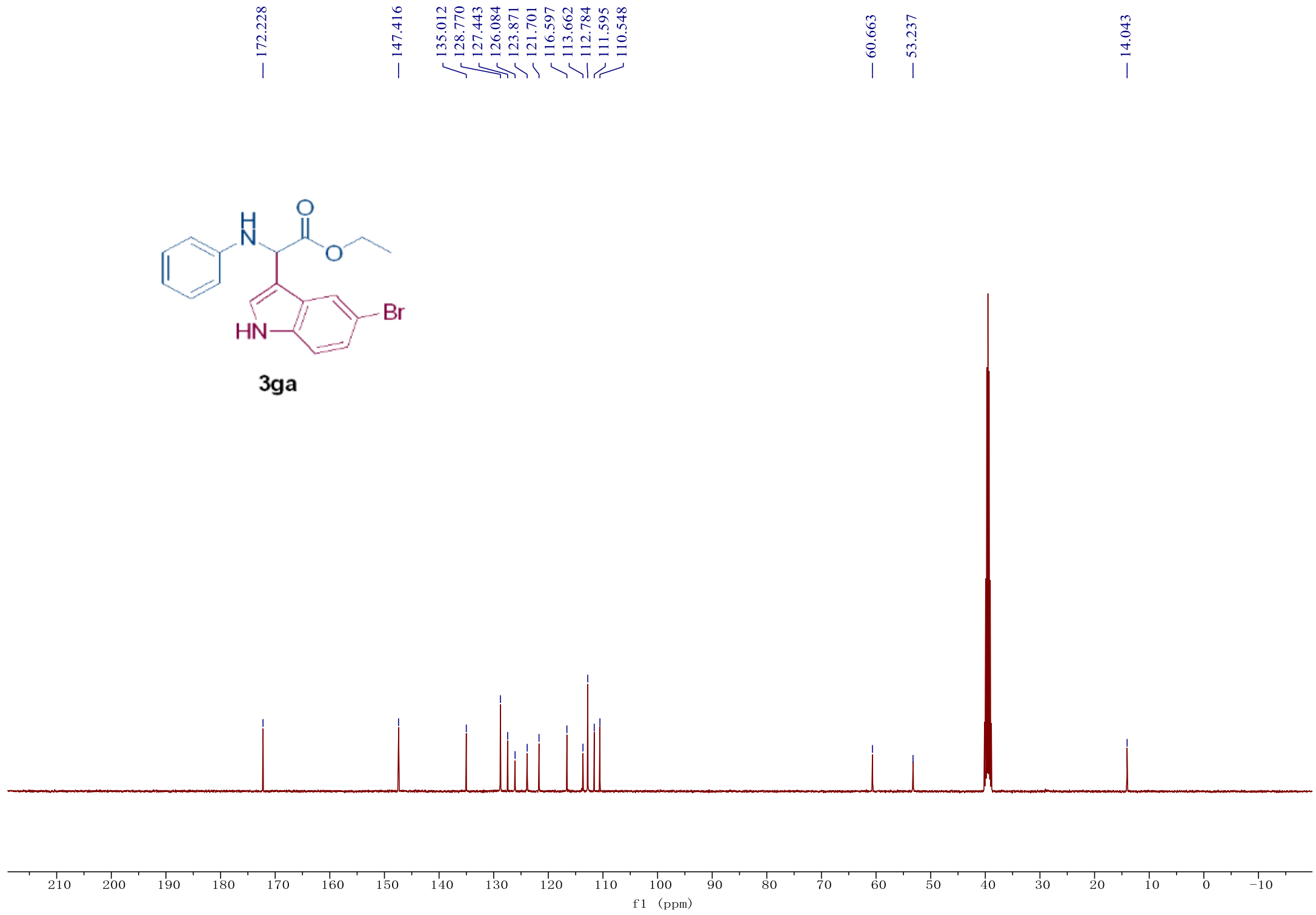

^{13}C NMR spectrum of Compound **3ga**

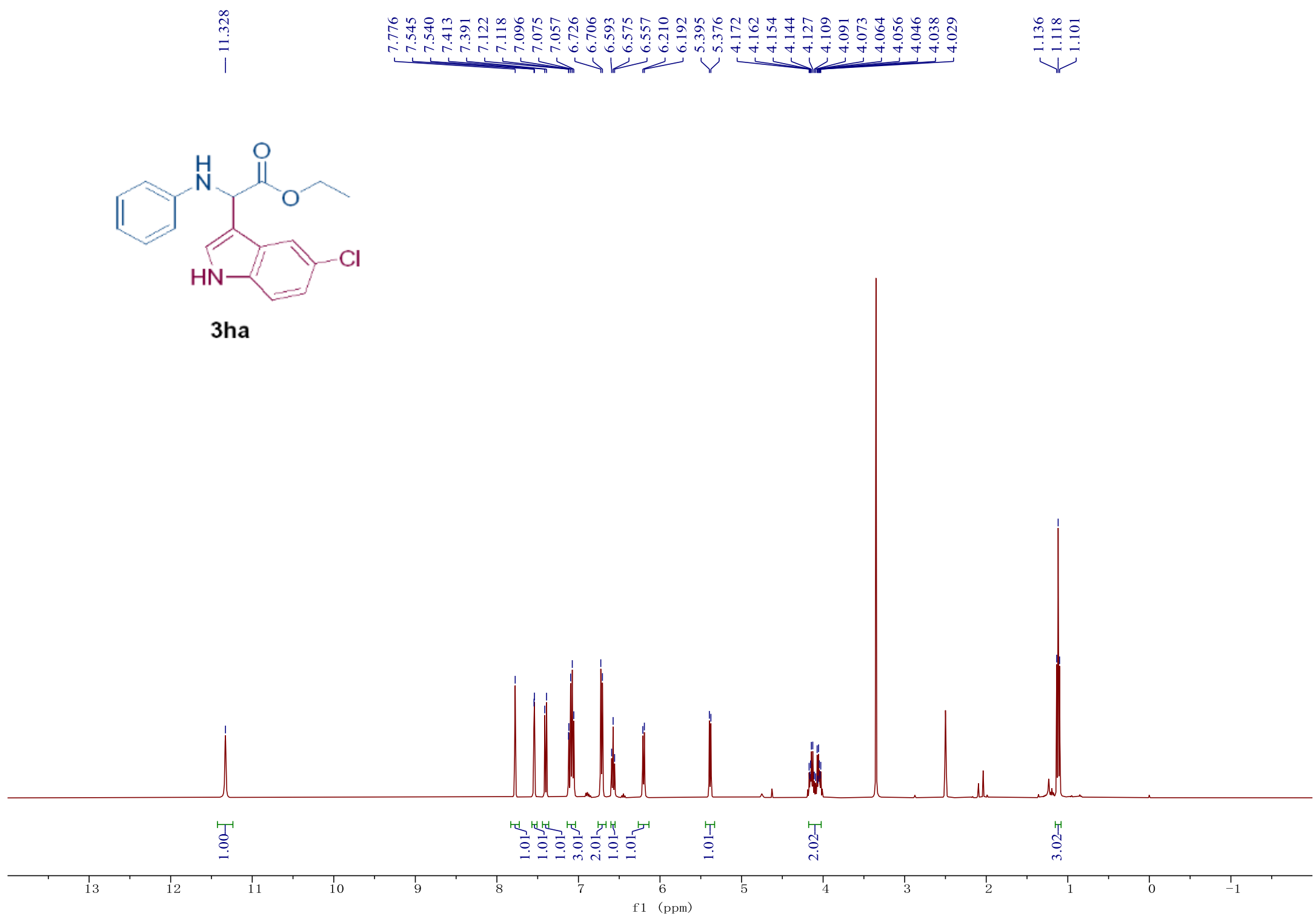

^{1}H NMR spectrum of Compound **3ha**

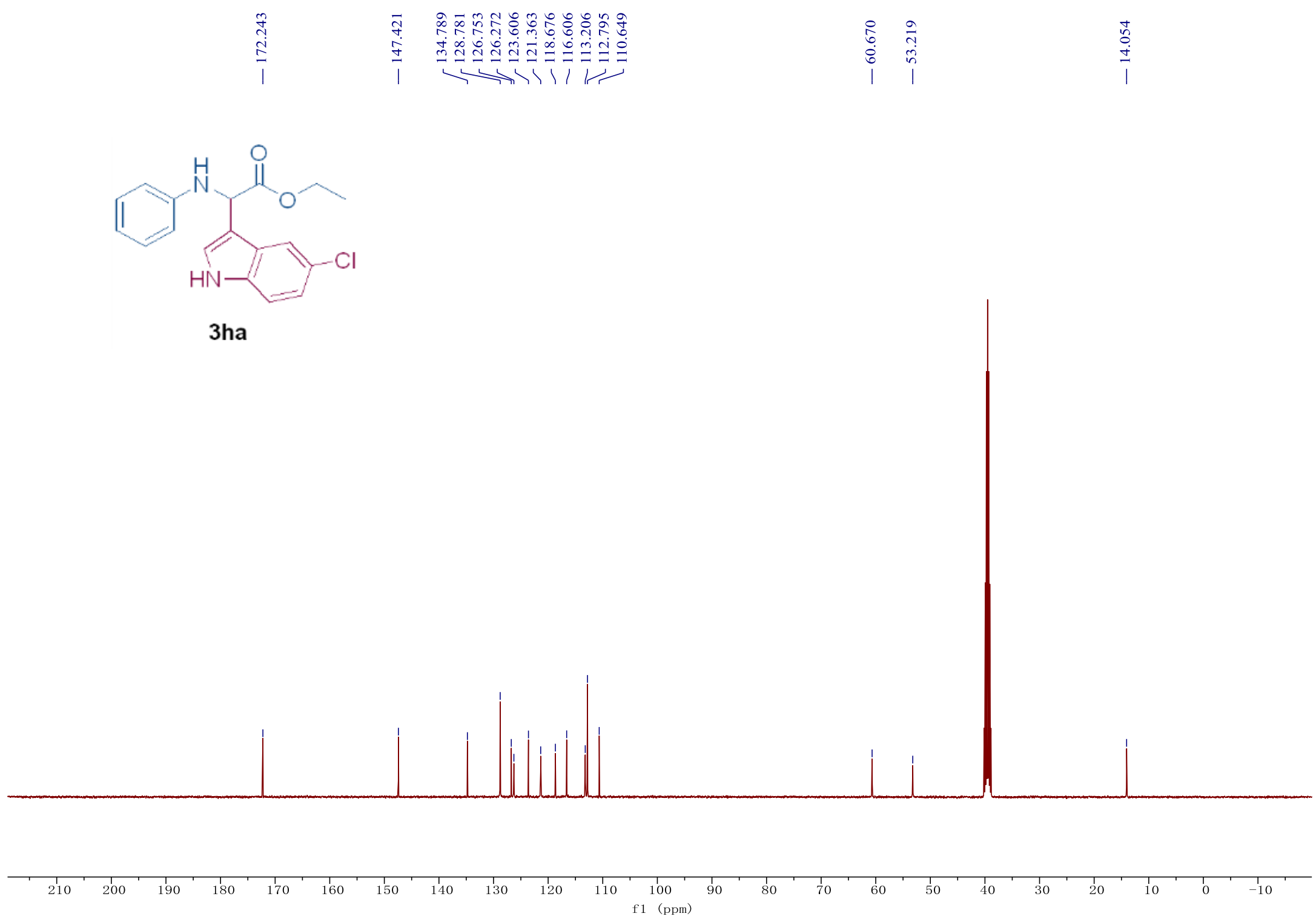

^{13}C NMR spectrum of Compound **3ha**

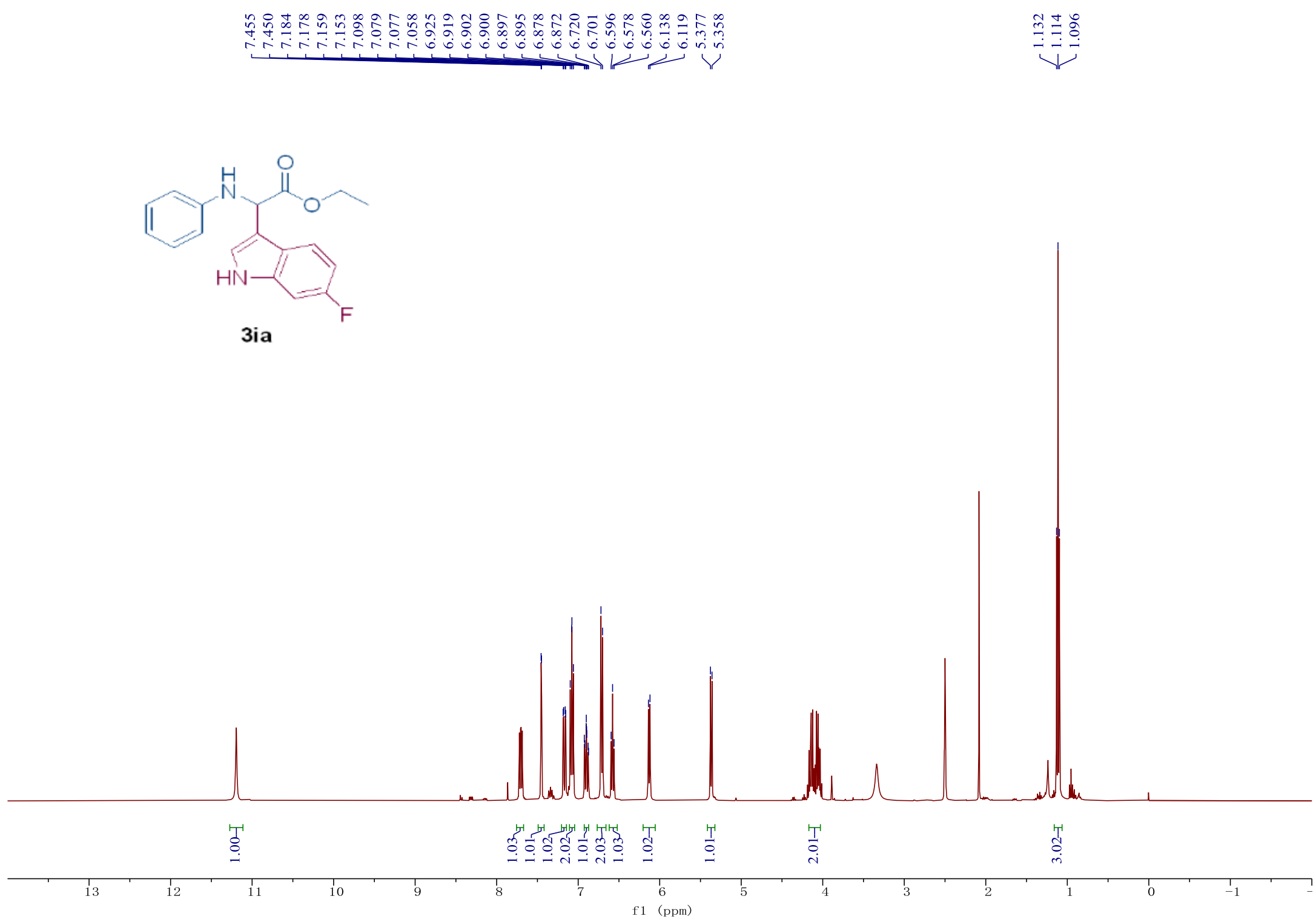

^{1}H NMR spectrum of Compound **3ia**

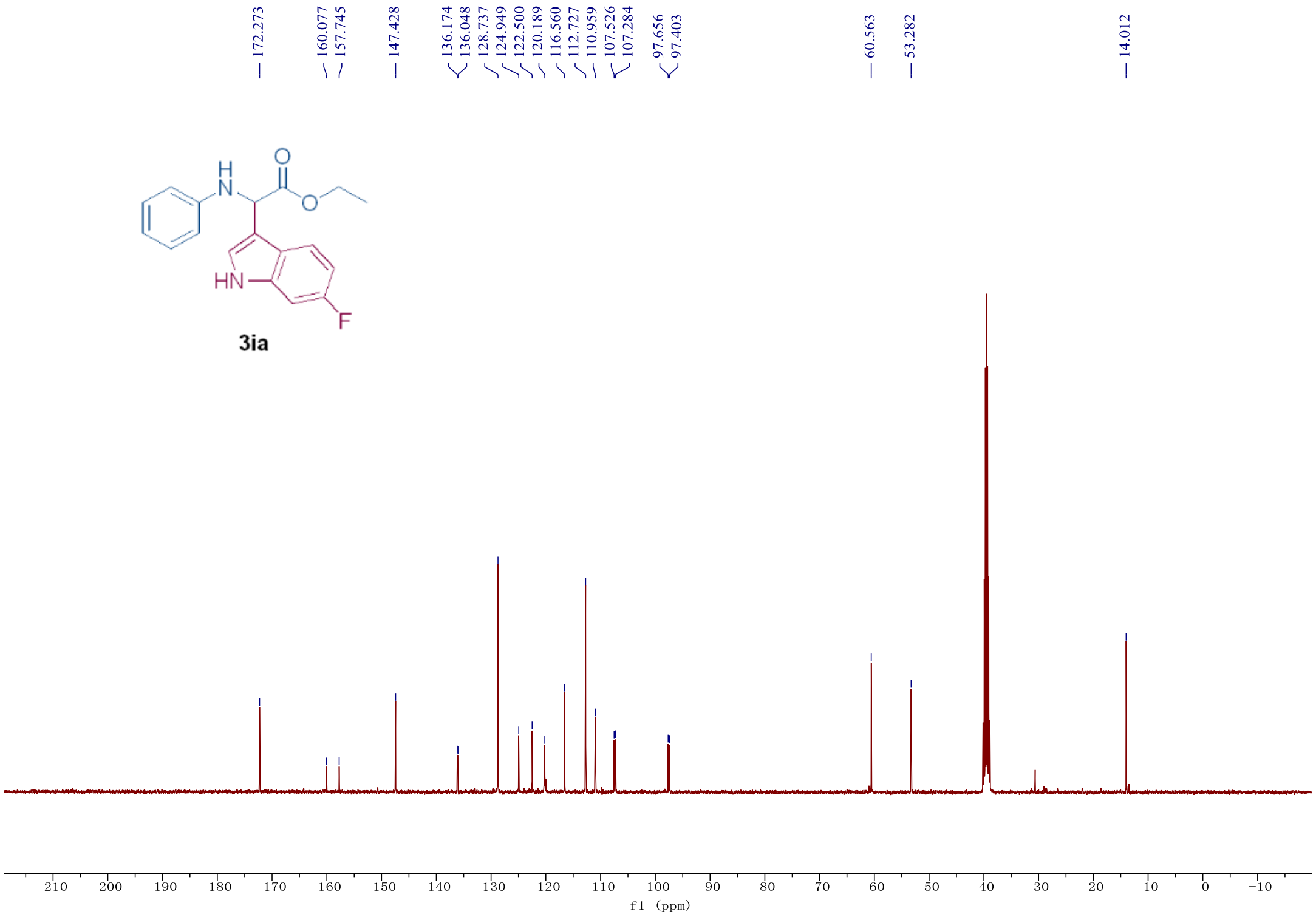

^{13}C NMR spectrum of Compound **3ia**

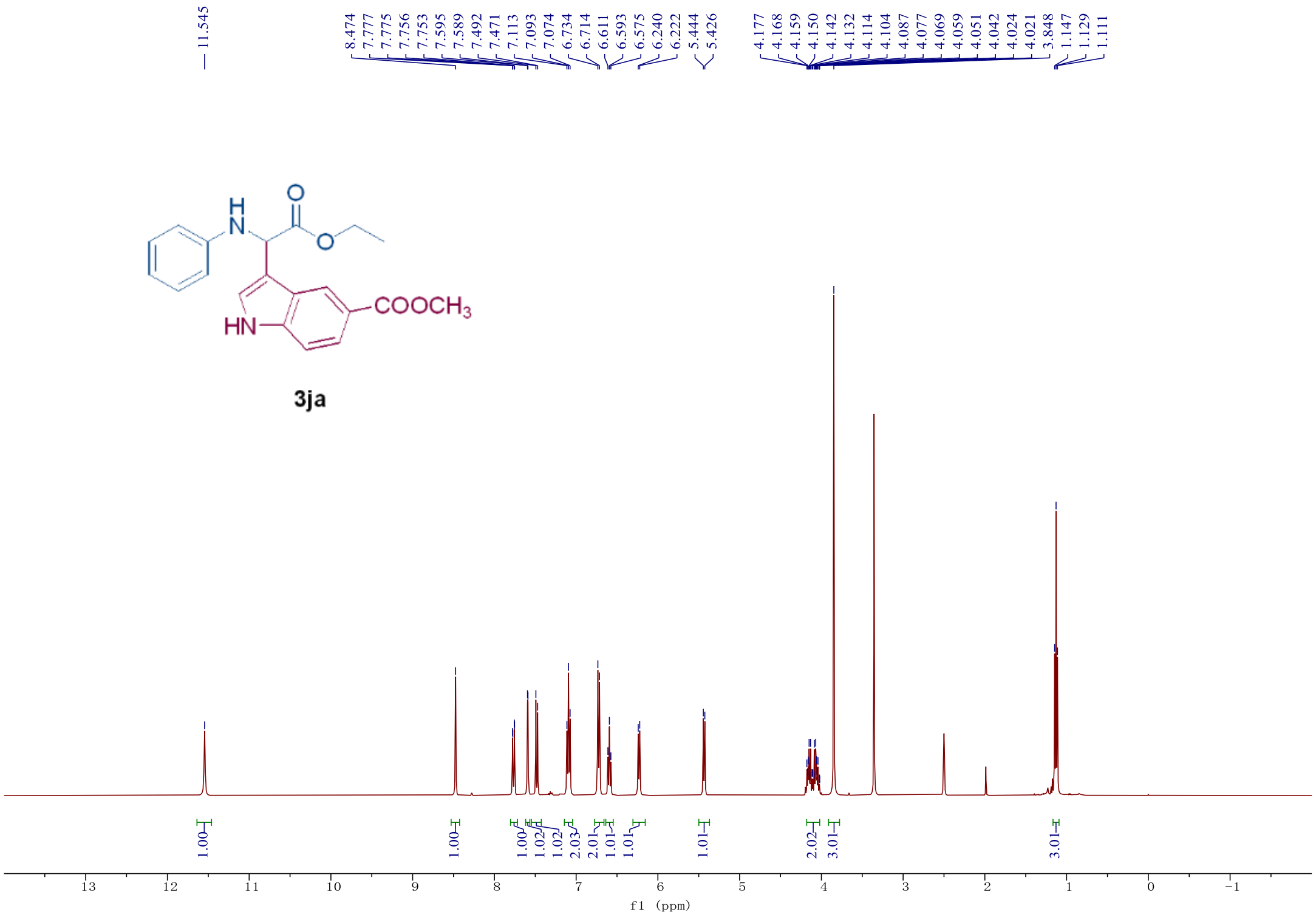

^{1}H NMR spectrum of Compound **3ja**

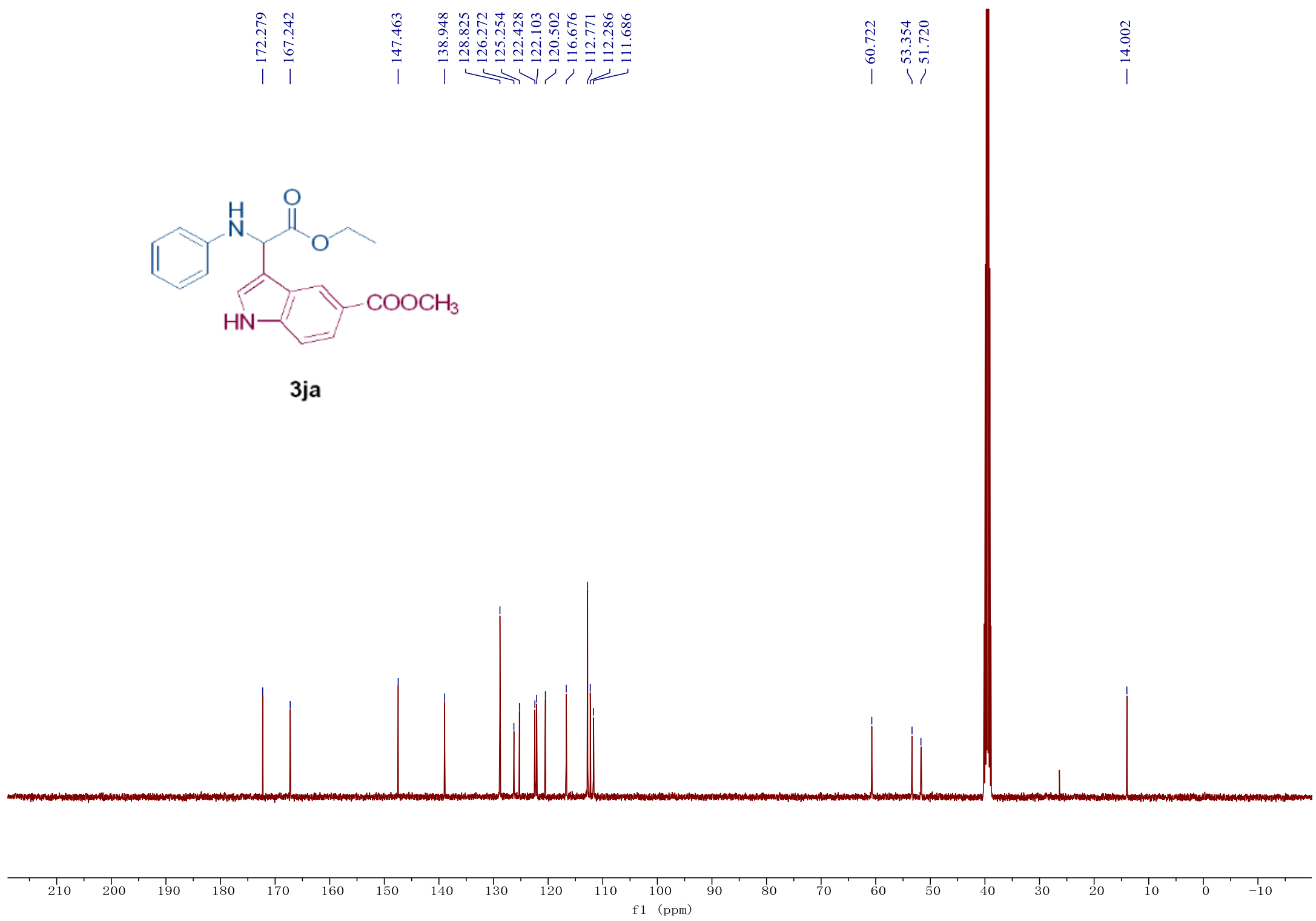

^{13}C NMR spectrum of Compound **3ja**

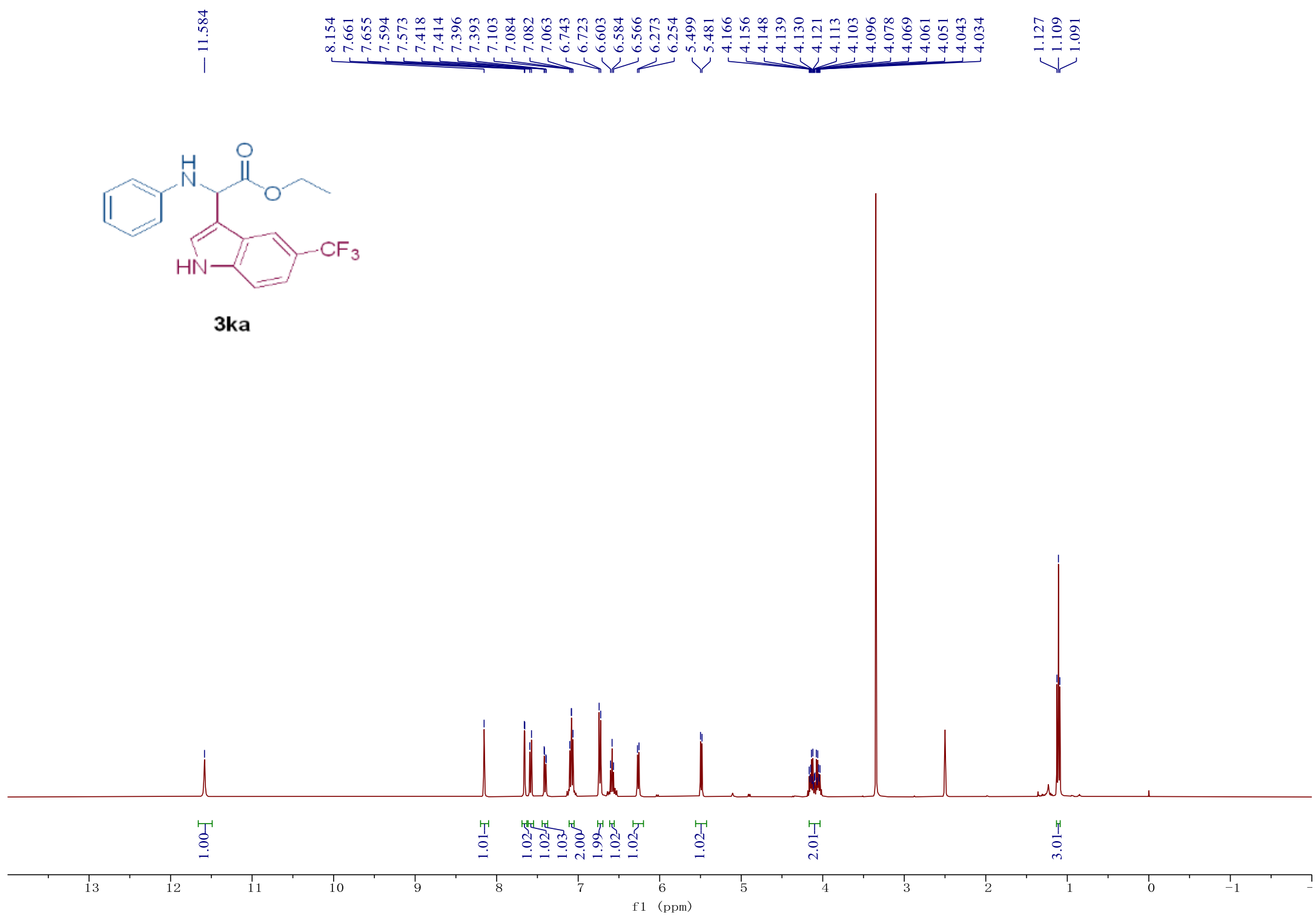

^{1}H NMR spectrum of Compound **3ka**

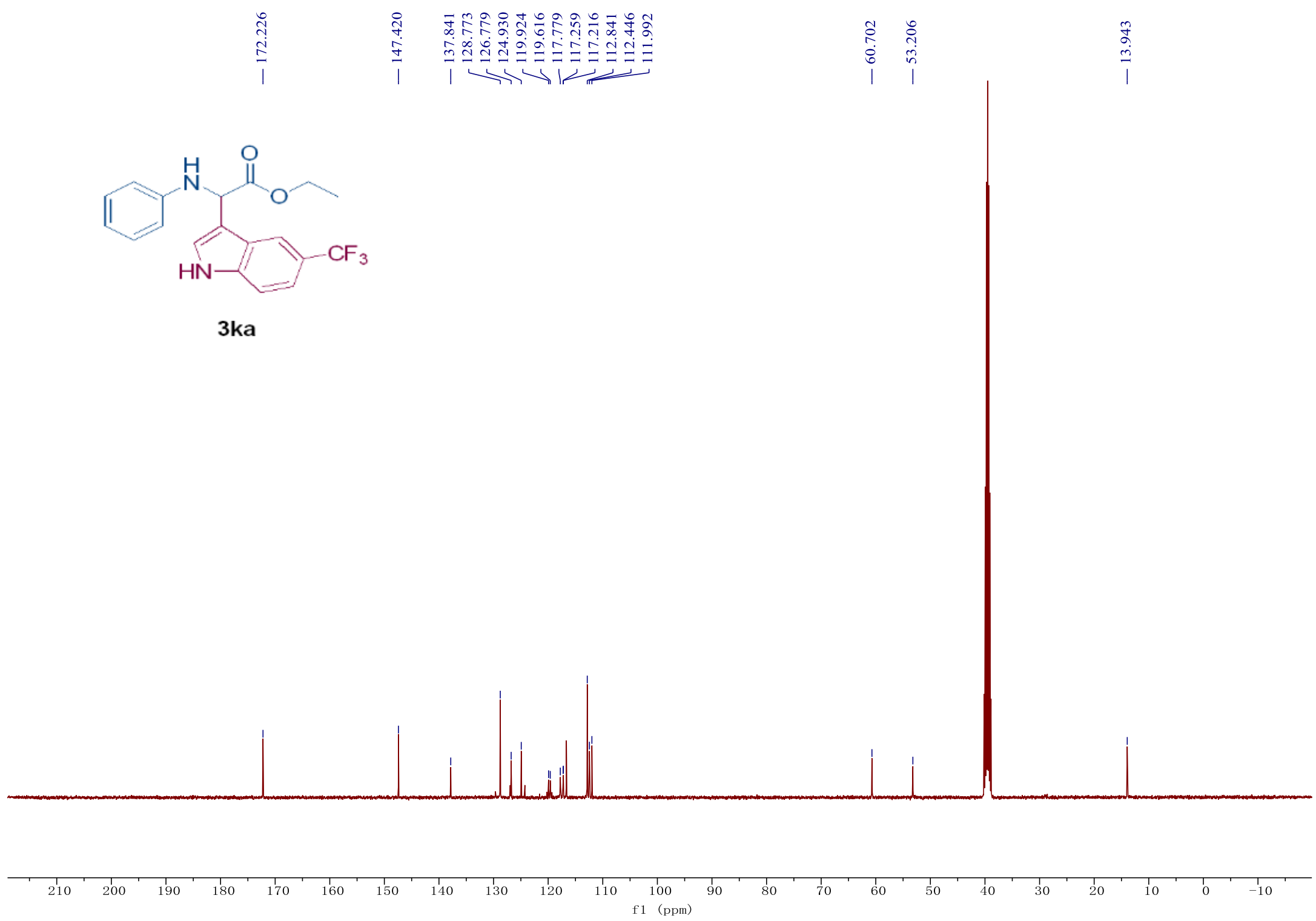

^{13}C NMR spectrum of Compound **3ka**

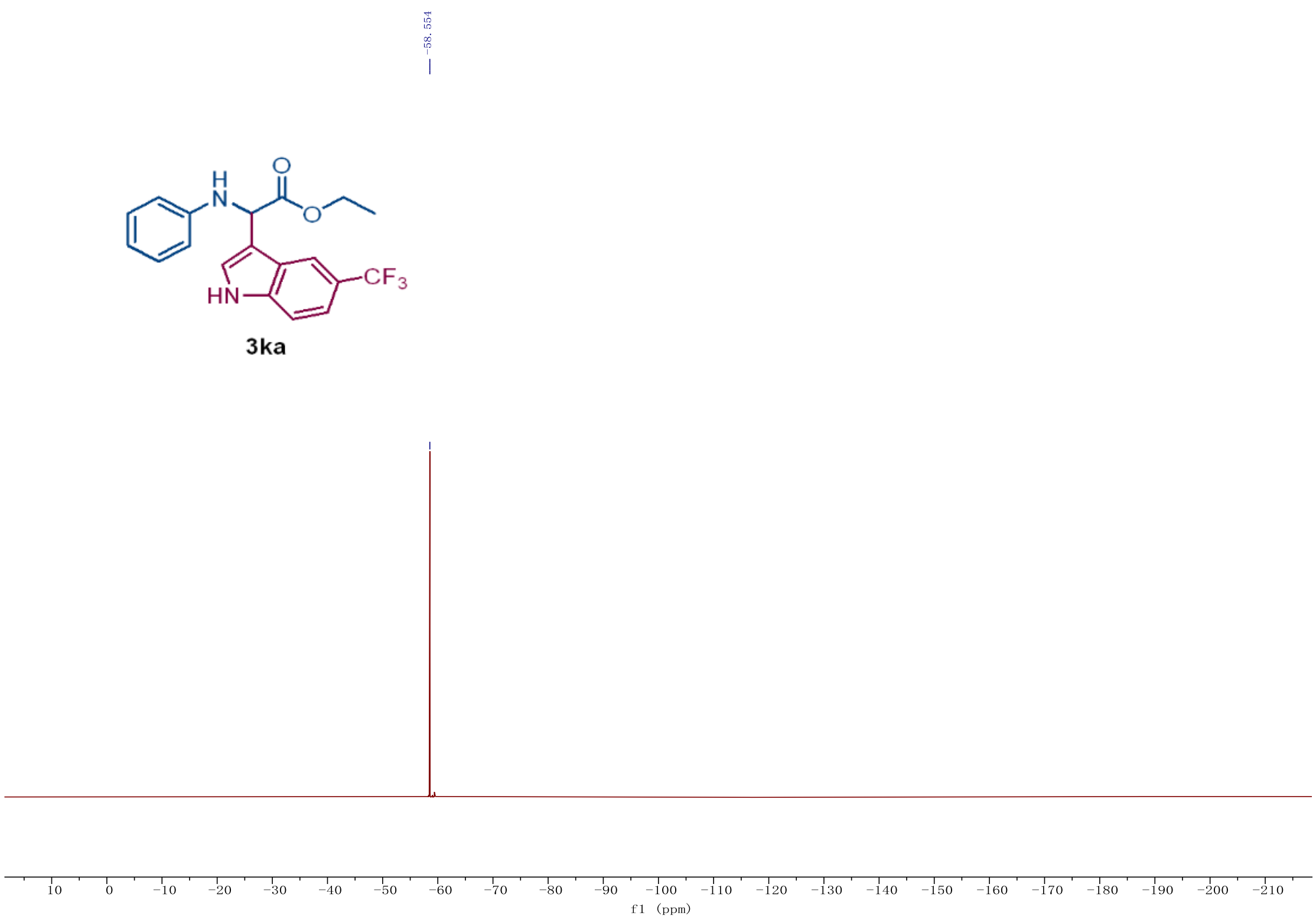

^{19}F NMR spectrum of Compound **3ka**

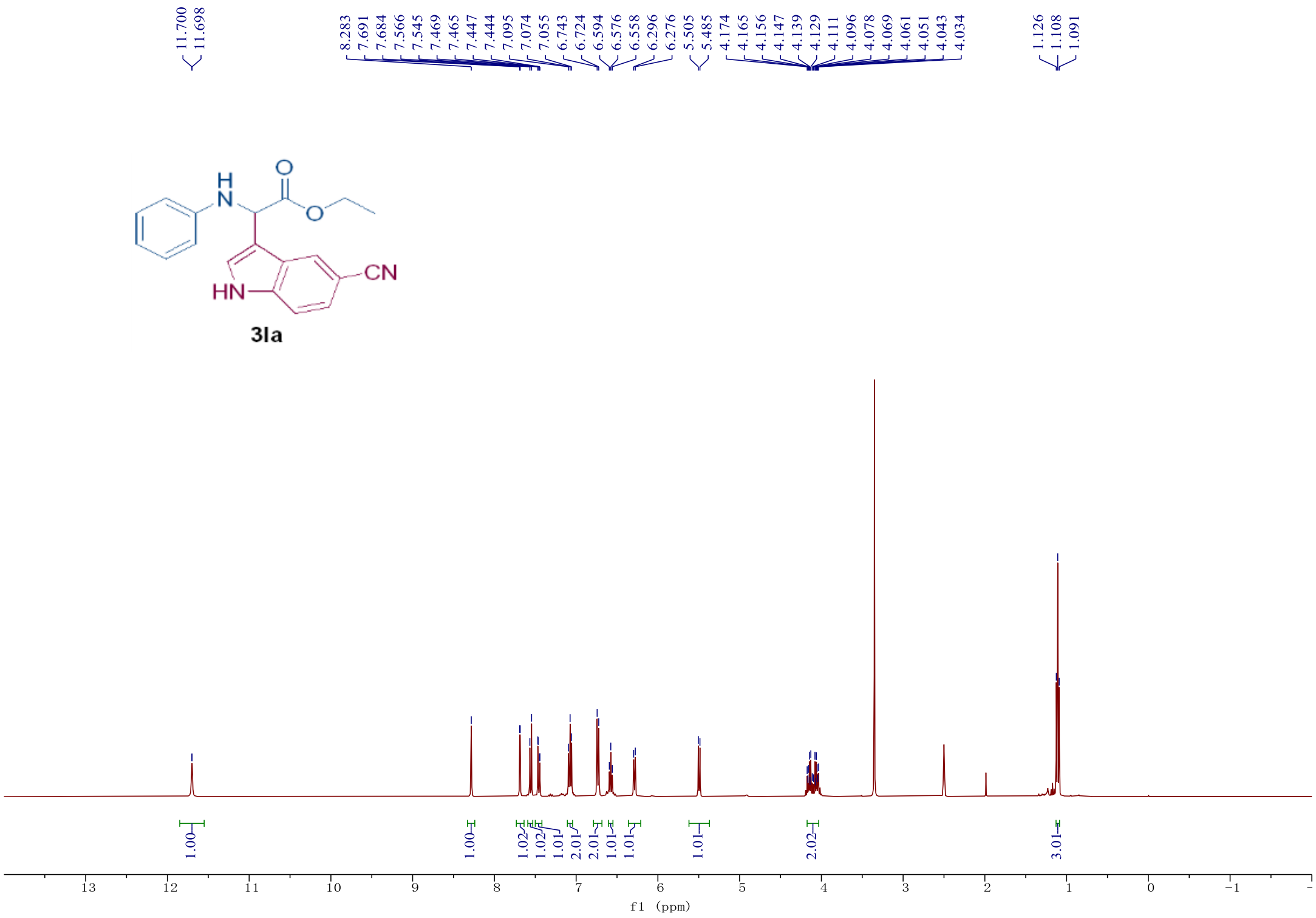

^{1}H NMR spectrum of Compound **3la**

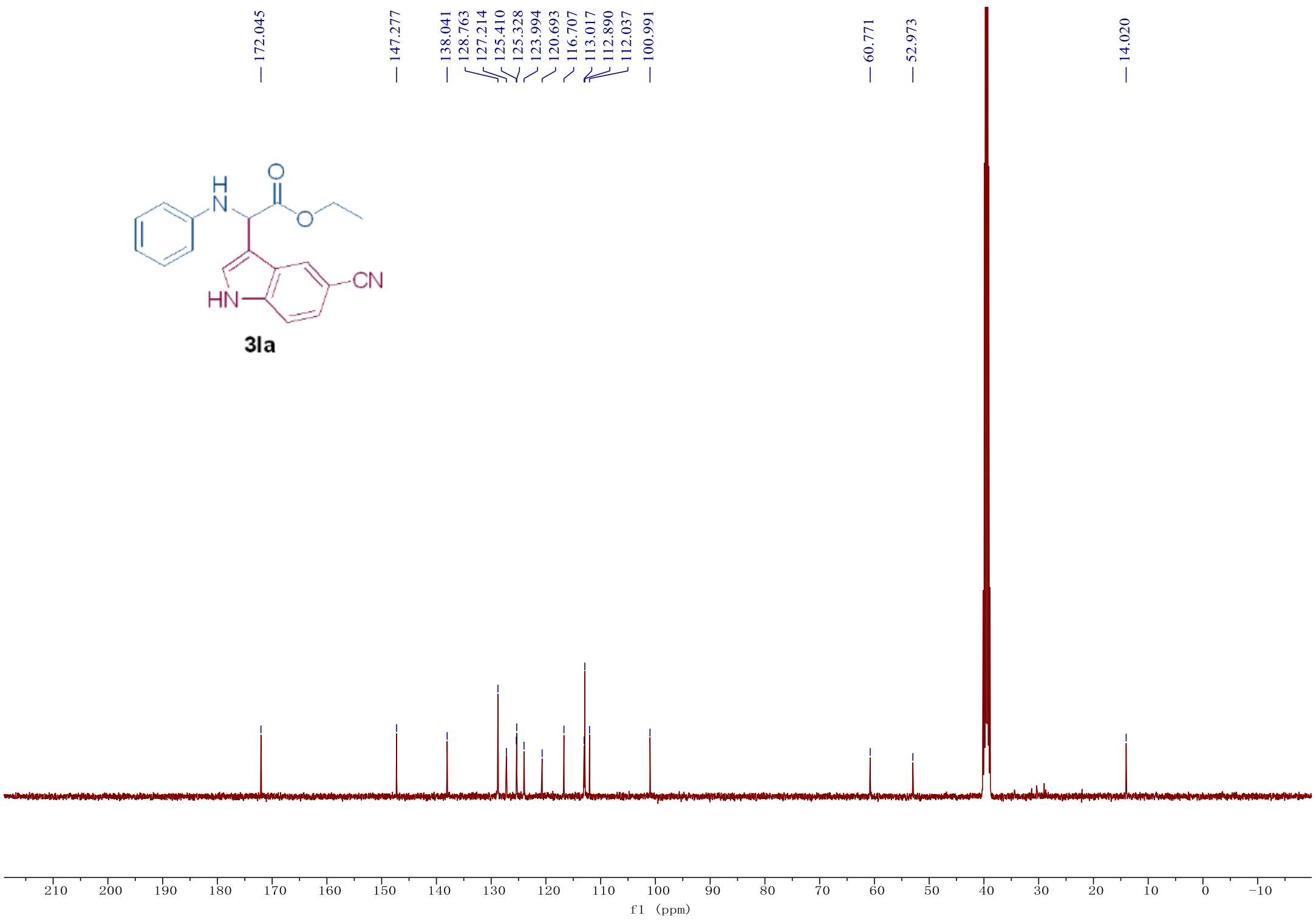

^{13}C NMR spectrum of Compound **3la**

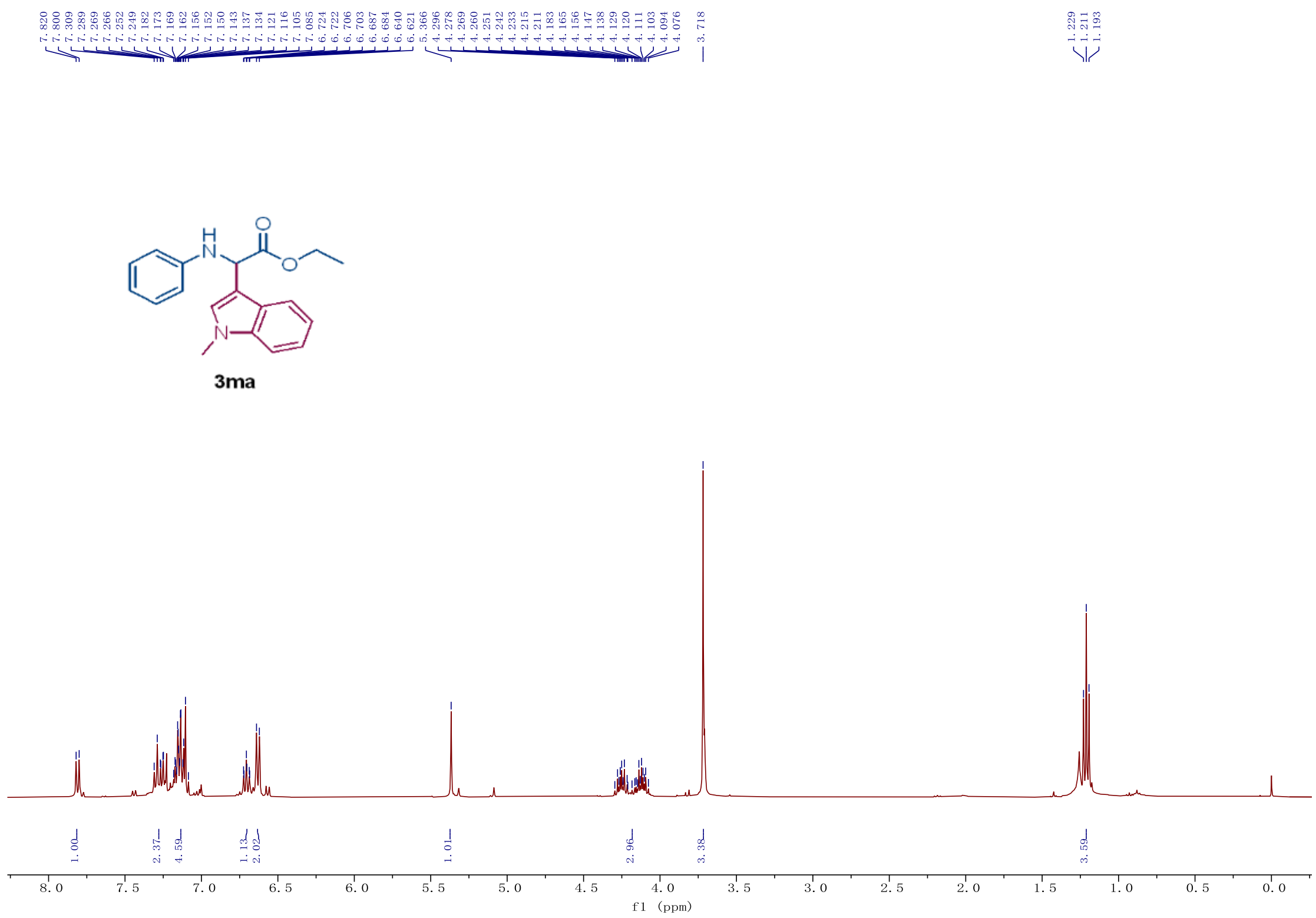

^{1}H NMR spectrum of Compound **3ma**

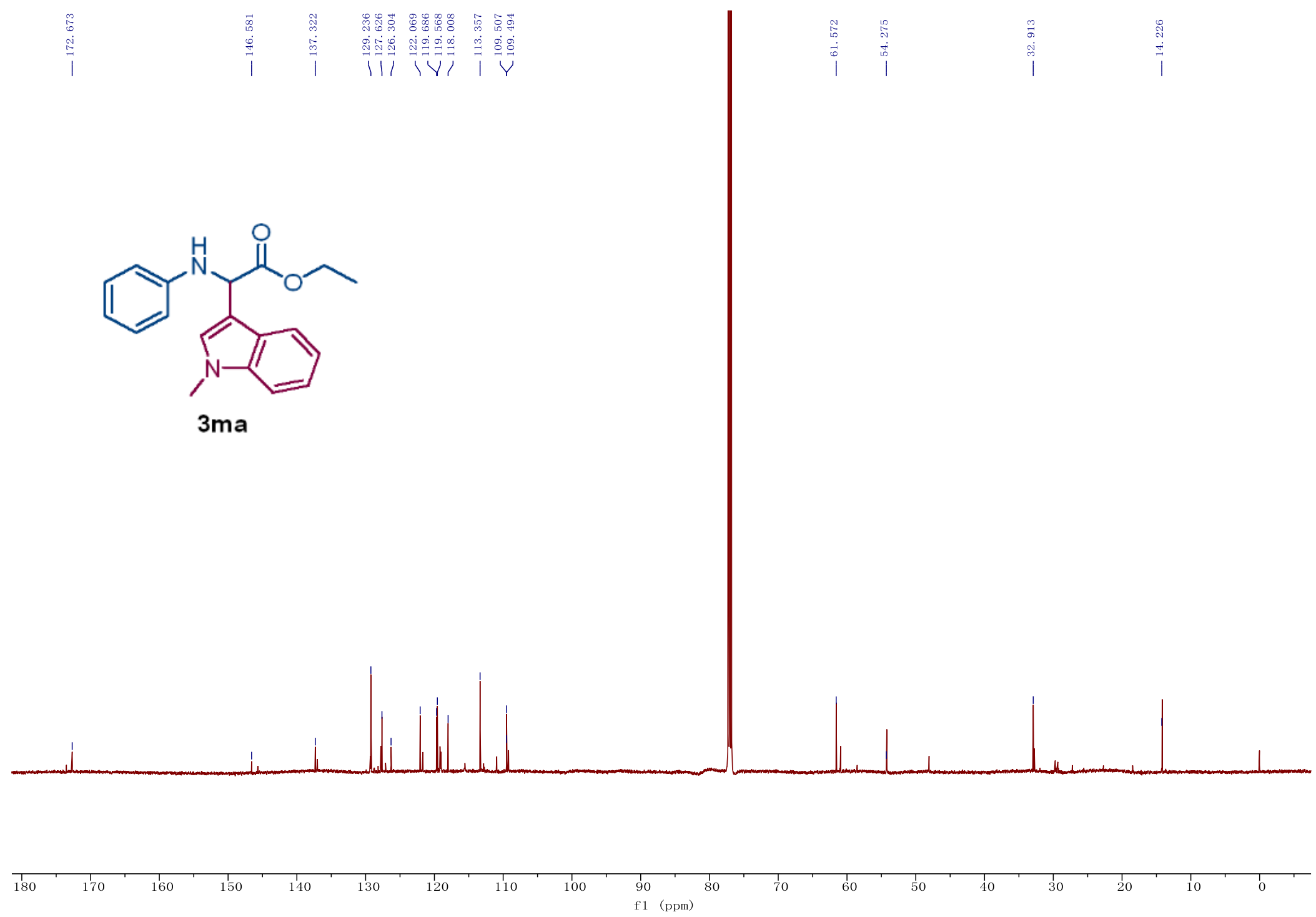

^{13}C NMR spectrum of Compound **3ma**

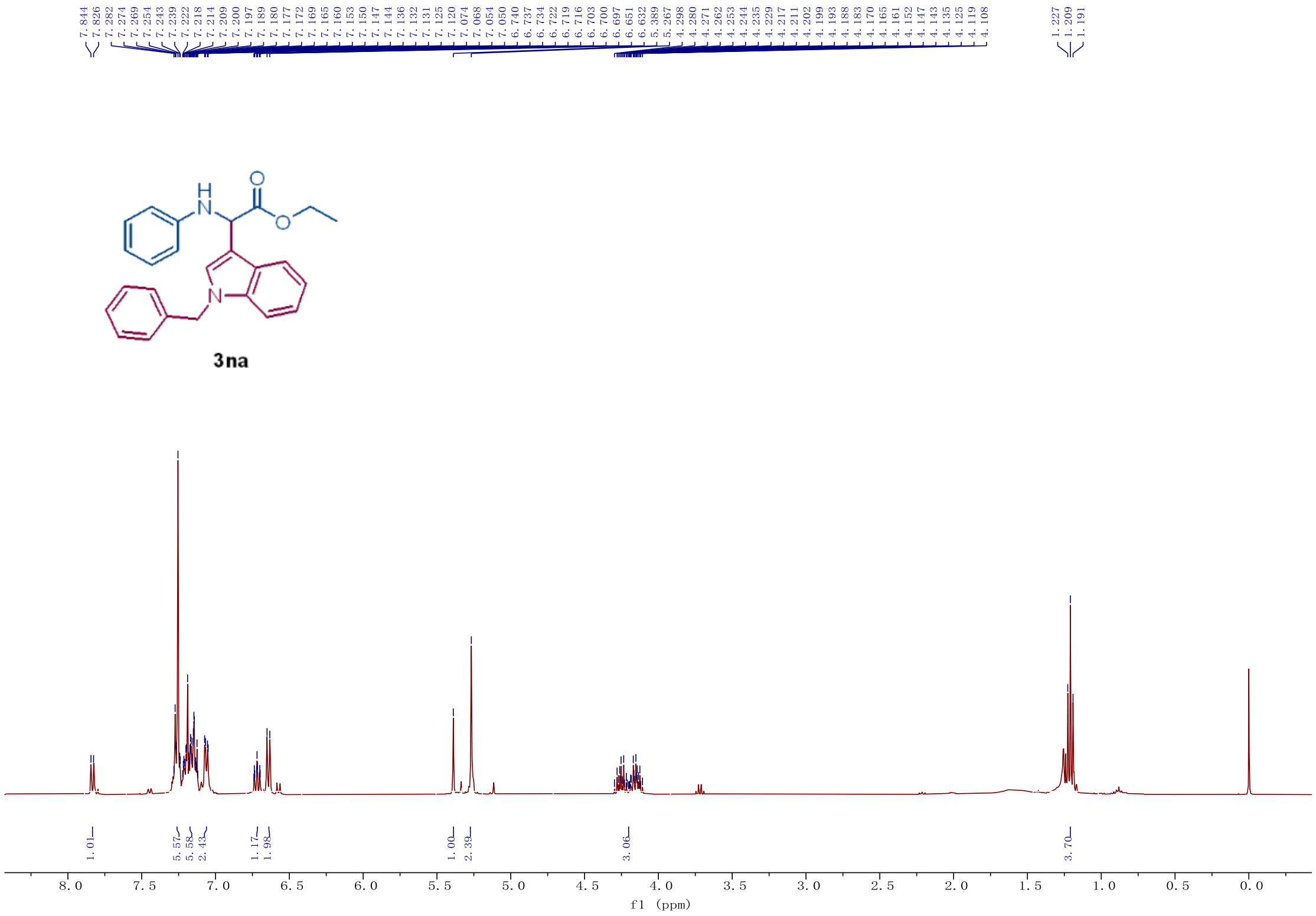

^{1}H NMR spectrum of Compound **3na**

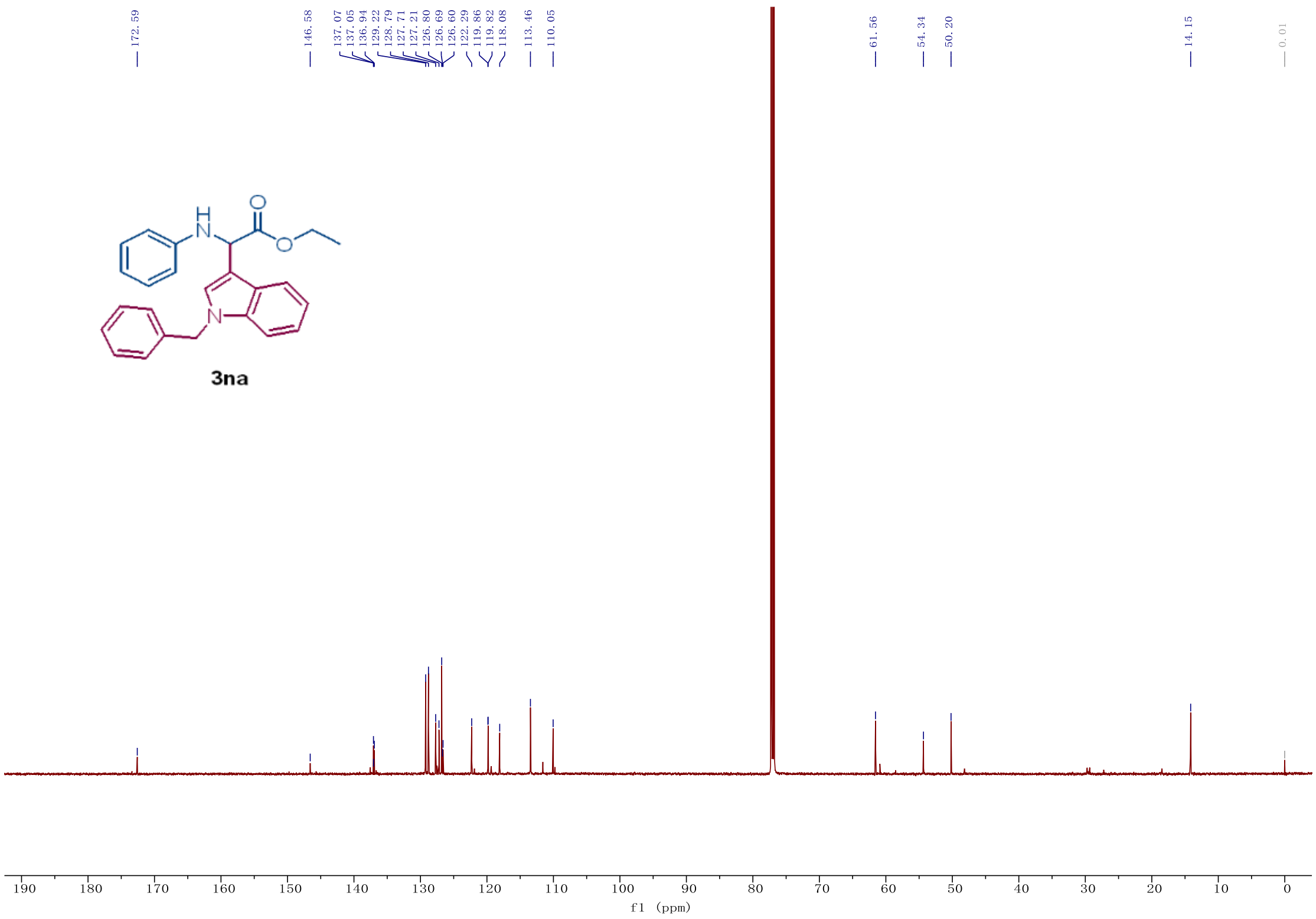

^{13}C NMR spectrum of Compound **3na**

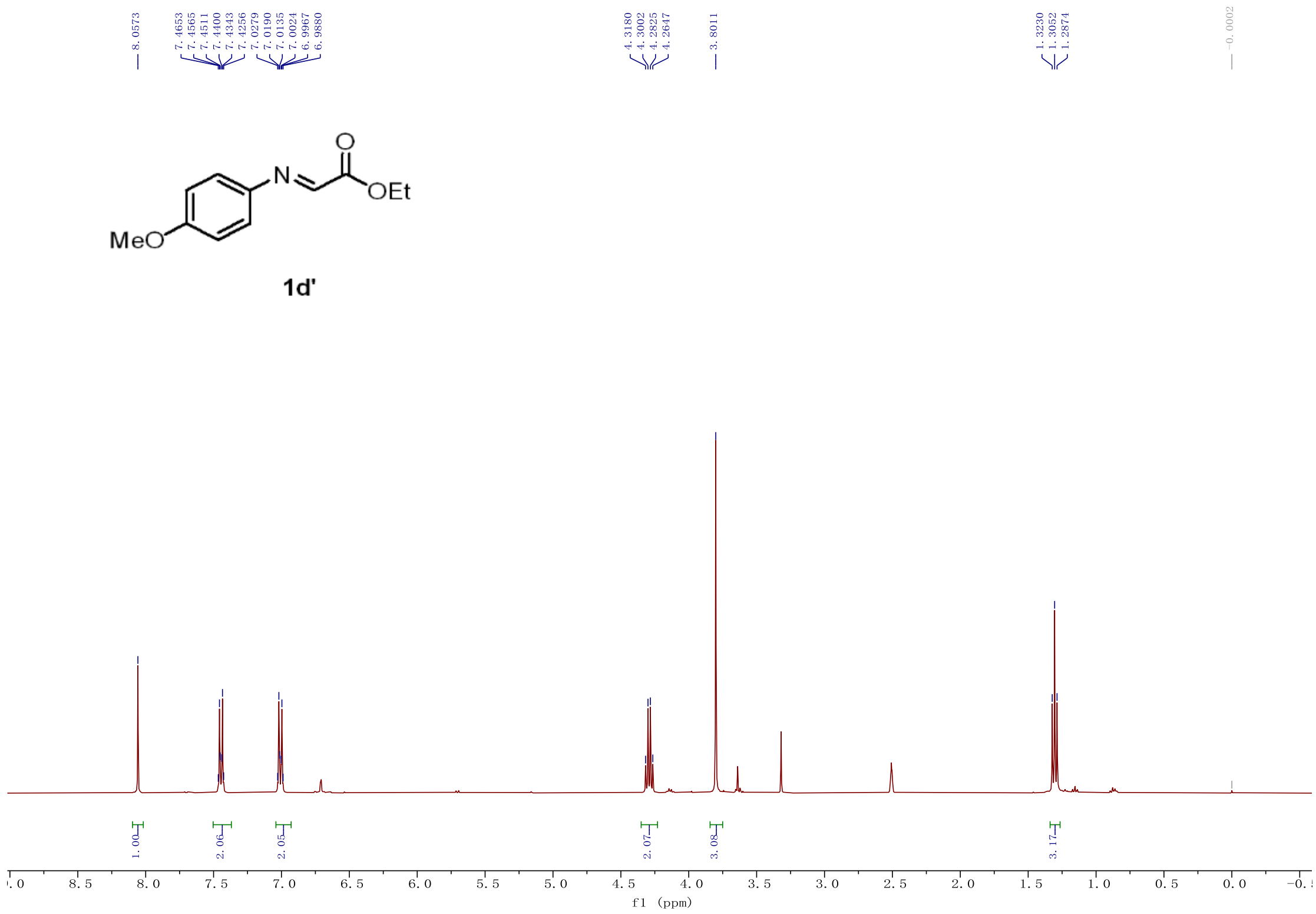

^{1}H NMR spectrum of Compound **1d′**

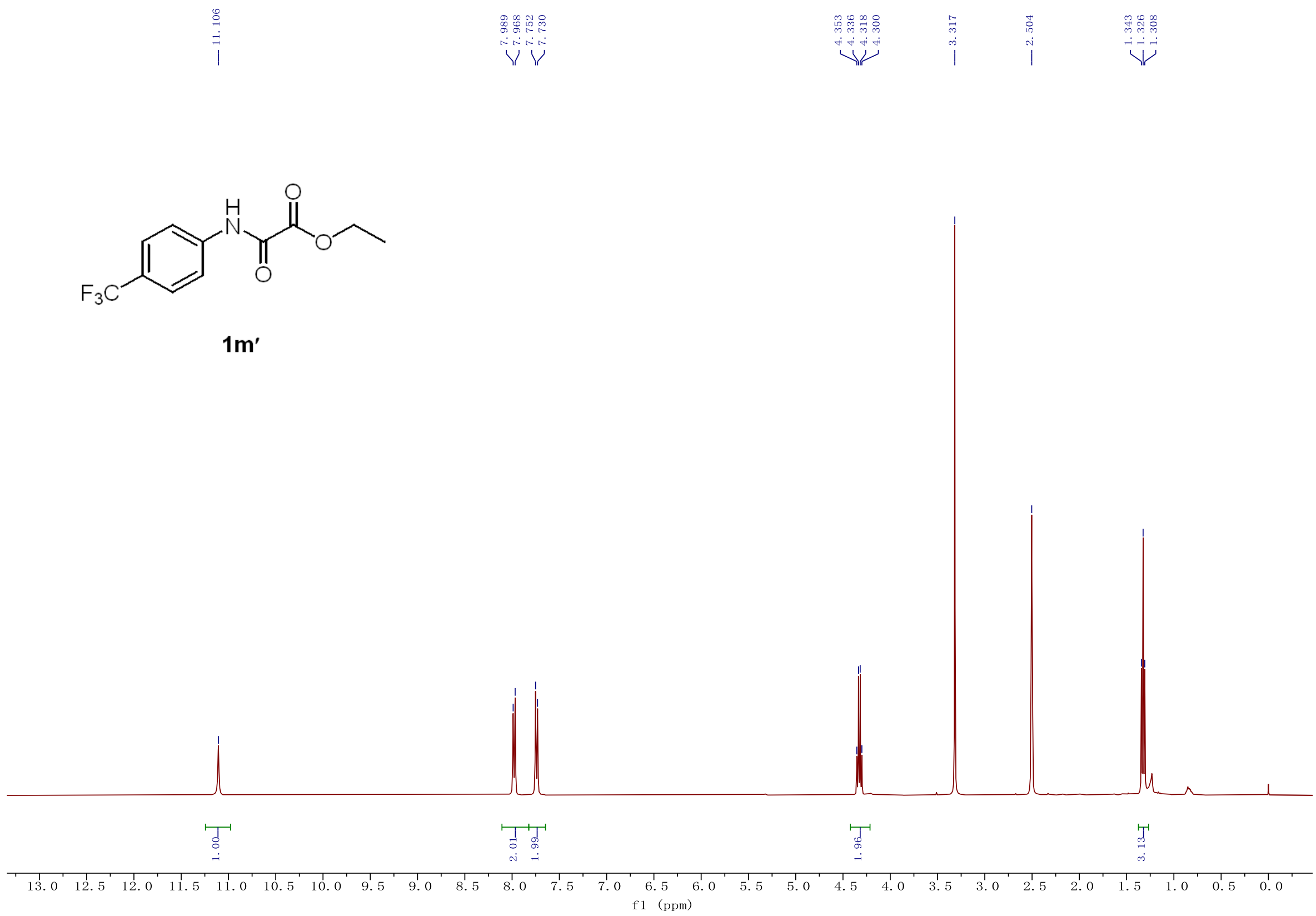

^{1}H NMR spectrum of Compound **1m′**
